# Supplementary material for: Prevalence and risk factors for wheeze, decreased forced expiratory volume in 1 s and bronchoconstriction in young children living in Havana, Cuba: a population-based cohort study
Source: BMJ Open. 2020 Apr 22;10(4):e034192. doi: 10.1136/bmjopen-2019-034192 (PMC7204937; doi:10.1136/bmjopen-2019-034192)
Supplement: Author's manuscript [file bmjopen-2019-034192.draft_revisions.pdf]

BMJ Open is committed to open peer review. As part of this commitment we make the peer review history of every article we publish publicly available.

When an article is published we post the peer reviewers' comments and the authors' responses online. We also post the versions of the paper that were used during peer review. These are the versions that the peer review comments apply to.

The versions of the paper that follow are the versions that were submitted during the peer review process. They are not the versions of record or the final published versions. They should not be cited or distributed as the published version of this manuscript.

BMJ Open is an open access journal and the full, final, typeset and author-corrected version of record of the manuscript is available on our site with no access controls, subscription charges or pay-per-view fees (http://bmjopen.bmj.com).

If you have any questions on BMJ Open's open peer review process please email info.bmjopen@bmj.com

BMJ Open

Prevalence and risk factors for wheeze, decreased forced expiratory volume in one second and bronchoconstriction in young children living in Havana, Cuba: A prospective population-based study

Journal:	*BMJ Open*
Manuscript ID	bmjopen-2019-034192
Article Type:	Original research
Date Submitted by the Author:	10-Sep-2019

Complete List of Authors:	Suarez-Medina, Ramon; INHEM<br>Venero-Fernández, Silvia; INHEM<br>Corona-Tamayo, Barbara; Dirección Municipal de Salud Pública municipios Cerro y Arroyo Naranjo<br>Betancourt-López, Maria; INHEM<br>Alvarez-Valdés, Vilma; Dirección Municipal de Salud Pública municipios Cerro y Arroyo Naranjo<br>Sardiñas-Baez, Nieves; Dirección Municipal de Salud Pública municipios Cerro y Arroyo Naranjo<br>Cristina, Carmona; Dirección Municipal de Salud Pública municipios Cerro y Arroyo Naranjo<br>Loinaz-Gonzalez, Maria; Dirección Municipal de Salud Pública municipios Cerro y Arroyo Naranjo<br>Verdecia-Pérez, Zunilda; Dirección Municipal de Salud Pública municipios Cerro y Arroyo Naranjo<br>Britton, John; UK Center for Tobacco and Alcohol Studies, Division of Epidemiology and Public Health<br>Fogarty, Andrew; University of Nottingham,

Keywords:	PAEDIATRICS, Asthma < THORACIC MEDICINE, Cuba

SCHOLARONE™
Manuscripts

I, the Submitting Author has the right to grant and does grant on behalf of all authors of the Work (as defined in the below author licence), an exclusive licence and/or a non-exclusive licence for contributions from authors who are: i) UK Crown employees; ii) where BMJ has agreed a CC-BY licence shall apply, and/or iii) in accordance with the terms applicable for US Federal Government officers or employees acting as part of their official duties; on a worldwide, perpetual, irrevocable, royalty-free basis to BMJ Publishing Group Ltd ("BMJ") its licensees and where the relevant Journal is co-owned by BMJ to the co-owners of the Journal, to publish the Work in this journal and any other BMJ products and to exploit all rights, as set out in our licence.

The Submitting Author accepts and understands that any supply made under these terms is made by BMJ to the Submitting Author unless you are acting as an employee on behalf of your employer or a postgraduate student of an affiliated institution which is paying any applicable article publishing charge ("APC") for Open Access articles. Where the Submitting Author wishes to make the Work available on an Open Access basis (and intends to pay the relevant APC), the terms of reuse of such Open Access shall be governed by a Creative Commons licence – details of these licences and which Creative Commons licence will apply to this Work are set out in our licence referred to above.

Other than as permitted in any relevant BMJ Author's Self Archiving Policies, I confirm this Work has not been accepted for publication elsewhere, is not being considered for publication elsewhere and does not duplicate material already published. I confirm all authors consent to publication of this Work and authorise the granting of this licence.

For peer review only

Prevalence and risk factors for wheeze, decreased forced expiratory volume in one second and bronchoconstriction in young children living in Havana, Cuba: A prospective population-based study

* Ramon Suarez-Medina[1]
Silvia Josefina Venero-Fernández[1]
Vilma Alvarez-Valdés[2]
Nieves B Sardiñas-Baez[2]
Cristina Carmona[2]
María Luisa Loinaz-Gonzalez[2]
Zunilda Verdecia-Pérez[2]
Barbara Corona-Tamayo[2]
María Antonia Betancourt-López[1]
John Britton[3],
Andrew W Fogarty[3]
and the HINASIC (Historia Natural de la Sibilancia en Cuba/Natural History of Wheezing in Cuba) Study Group[++]

[1] Instituto Nacional de Higiene, Epidemiología y Microbiología, Infanta No 1158 e/ Llinás y Clavel, Código Postal 10300, La Habana, Cuba.
[2] Dirección Municipal de Salud Pública municipios Cerro y Arroyo Naranjo, La Habana, Cuba.
[3] Nottingham Biomedical Research Unit, Division of Epidemiology and Public Health, University of Nottingham, Clinical Sciences Building, City Hospital, Nottingham NG5 1PB, UK
[++]full list of collaborating researchers at the end of the manuscript

The corresponding author for the manuscript submission process is Dr Andrew Fogarty (Andrew.fogarty@nottingham.ac.uk) due to poor internet connections with Cuba.

(If accepted for publication) *Correspondence and requests for reprints to Dr. Ramón Suárez Medina:
Address: Infanta No 1158 e/ Llinás y Clavel, Código Postal 10300, La Habana, Cuba.
Office phone: (537) 878 8479
Email: ramonsm@inhem.sld.cu

Key words: Cuba, asthma, children, wheeze

Word count: 3138 words

For peer review only

Abstract (298 words)

Objectives; Asthma has not been extensively studied in low and middle-income countries, where risk factors and access to treatment may differ from more affluent countries. we aimed to identify the prevalence of asthma and local risk factors in Havana, Cuba.

Setting: Four municipalities in Havana, Cuba.

Participants: A population-based cohort study design of young children living in Havana, Cuba. Children were recruited from primary care centres at age 12-15 months.

Primary and secondary outcome measures: Data on wheeze in the past 12 months, asthma treatment and environmental exposures collected regularly until the age of six years, when Forced Expiratory Volume in one second (FEV_1) and reversibility to aerosolised salbutamol were also measured.

Results: 1106 children provided data at the age of six years old. The prevalence of wheeze in the previous 12 months was 422 (38%), and 331 (37%) of the study population had bronchodilatation of 10% or more in FEV_1 after administration of inhaled salbutamol. In the previous 12 months, 182 (16%) of the children had received inhaled corticosteroids, 416 (38%) salbutamol inhalers, and 283 (26%) a course of systemic steroids.

Wheeze in the first year and a family history of asthma were both positively associated with bronchodilatation to inhaled salbutamol (+1.94%; 95% confidence intervals CI: +0.81 to +3.08, and +1.85%; CI: +0.14 to +3.57 respectively), while paracetamol use in the first year was associated with wheeze at six years (OR 1.64, 95% CI: 1.14 to 2.35). There were large differences in FEV_1, bronchodilatation and risk of wheeze across different geographical areas.

Conclusions: Asthma is common in young children living in Havana, and the high prevalence of systemic steroids administrated is likely to reflect the underuse of regular inhaled corticosteroids. If replicated in other comparable low and middle-income countries, this represents an important global public health issue.

Keywords: infection, asthma, children, Cuba, lung function

For peer review only - http://bmjopen.bmj.com/site/about/guidelines.xhtml

STRENGTHS AND LIMITATIONS OF THIS STUDY

- There have been many epidemiological studies of asthma in children who live in developed countries, but few population-based studies that have used objective measures of asthma in developing countries.
- The prevalence of bronchoconstriction in young children living in Havana is high using both subjective and objective measures of asthma.
- These data are from one developing country and thus not generalisable to other nations with different economies and health-care systems.
- The high prevalence of administration of systemic steroids in this population-based sample is public health concern, as it is likely to be avoidable, and may increase the risk of side-effects in later life.

For peer review only

Introduction

Asthma is a global disease that affects approximately 11% of six year-old children [1], but with marked regional differences in prevalence [1]. The aetiology of asthma is complex, and involves a range of environmental exposures that are likely to have differential impacts at different ages over the human lifetime [2-7]. The early years of childhood is a particularly important period as this is when the lungs and immune system are developing rapidly, and lung function in children is a key determinant of health in adulthood [8].

This study was established as a consequence of concerns among public health specialists and clinicians that asthma was becoming a large problem in Cuba. It aimed to determine the prevalence of wheeze in young children living in Cuba, and to identify modifiable risk factors for wheezing, reduced lung function and reversible bronchoconstriction. The role of infection in early life in the development of allergic disease remains unclear [9]. The main hypothesis of interest was that infection with parasites [10], *Helicobacter pylori* [11], dengue [12], or systemic inflammation [13] may be associated with wheeze or bronchoconstriction. Exposure to environmental tobacco smoke and paracetamol had previously been observed to be positively associated with wheeze [14] or atopic dermatitis [15] symptoms respectively, and so the association of these exposures with wheeze and bronchoconstriction were also studied. Finally, as growth from *in utero* onwards may also be related to development of asthma and growth of the lungs [16], anthropometric measures from birth onwards were also considered. The study design is a prospective population-based study of an existing cohort of children followed from approximately one year of age for five years.

Methods

Study population and sample collection

The study population is a cohort of 1956 children aged 12 to 15 months who were randomly selected from four municipalities across Havana in 2010 and 2011 [14 15 17]. Data was collected by a standardised questionnaire that was administered by a member of the study team at baseline and subsequently at 2 years, 3 years and 5 years later. This included a number of health and lifestyle questions that were answered by the parent or guardian and particular attention was paid to exposure to environmental tobacco smoke. The child's weight, height and mid-arm circumference in both arms were collected at each study visit. Historical baseline data including birth weight and height were collected from the primary care centre records. At each annual follow-up study the participants' guardians were asked if the child had received a medical diagnosis of dengue infection in the past year (in Cuba all positive diagnoses of dengue infection are clinically confirmed using a serological IgM assay) and a blood sample was collected from children to

For peer review only - http://bmjopen.bmj.com/site/about/guidelines.xhtml

For peer review only

measure circulating eosinophil levels. This sample was stored at -20 degrees Centigrade and subsequently defrosted and analysed for dengue immunoglobulin G (IgG) serology to generate an antibody index [18], serum immunoglobulin E (IgE) [17], highly sensitive C- Reactive Protein (hsCRP, SpinReact, Spain [19]), toxoplasmosis immunoglobulin G antibodies (IgG) [20] and toxocariasis IgG antibodies (DRG Instruments, Germany). A faecal sample was also collected at each review and stored at minus 20°C, and later examined for *Helicobacter pylori* using the faecal antigen test (SpinReact, Spain) and intestinal parasites using the Kato-Katz test (Campiñas Medical COMI, Brazil).

Lung function

Forced expiratory volume in one second (FEV_1) and Forced Vital Capacity were measured in accordance with ATS/ERS criteria [21] using spirometers (CareFusion Micro I) calibrated each day to allow for local climatic change. The best value of FEV_1 within a threshold of repeatability of 200ml was used as the final value. Aerosolised salbutamol (300 μg) was then administered via a spacer and after 15 minutes lung function was measured again to quantify airway reversibility. In children who provided a post-bronchodilator FEV_1 that was less than the baseline value, they were considered as having no reversibility to bronchodilator as this was likely to be due to fatigue.

Allergen skin prick testing

Skin prick testing was used to determine allergy to mite, cat, grass, cockroach, fungus, mosquitos, wheat and soy (allergens from Diater, Argentina except mite allergen from Biocen, Cuba). For each test a drop of allergen solution was placed on the skin and a lancet used to break the skin. After 15 minutes, the skin wheal was measured at its maximum diameter, and also perpendicularly, and a mean value generated. The final skin prick test result was calculated by subtracting the saline result from the allergen. A value of ≥3mm was used to define a positive atopic result for each allergen, and atopy was defined as any positive skin prick test.

Statistical analysis

The main outcome variables were FEV_1, percent increase in FEV_1 after to inhaled salbutamol and wheeze in the past 12 months. The main exposure variables were grouped into three categories:

1. Prior exposures: wheeze in the first year of life, family history of asthma, nursery attendance, birth weight, birth height, duration of breastfeeding, blood IgE and eosinophils at one year old; faecal *Helicobacter pylori* antigen at two and three years old; blood hsCRP, dengue IgG serology, eosinophils, toxoplasmosis serology, IgE at three years old, and any prior medical diagnosis of dengue infection.

For peer review only - http://bmjopen.bmj.com/site/about/guidelines.xhtml

2. Cross-section exposures: number of smokers living in the home, current weight, current height, mean arm circumference, municipality of residence.
3. Biomarkers of current infection and inflammation: Helicobacter pylori stool antigen, Toxoplasmosis IgG serology, dengue IgG serology, blood hsCRP, eosinophils, IgE, toxocariasis serology and atopy. Less than 2% of children has current gastro-intestinal parasite infection and these data were not analysed further.

Statistical analysis used linear and logistic regression adjusting for sex and age in months as *a priori* confounding factors, and also adjusted for clustering by municipality of residence. All analyses used Stata 14 statistical software (Texas, USA).

Patient and Public Involvement

The study was designed as a consequence of concerns from the Cuban public health and clinical communities about asthma morbidity. The patients were not involved in the design of the study and patients did not receive a copy of the results. We thank the patients and their families for their participation.

Ethics approval

The study was approved by Ethics Committees in the Instituto Nacional de Higiene, Epidemiología y Microbiología, Cuba and at the University of Nottingham, UK.

Results

Data were available for 1106 children, of whom 422 (38%) had reported wheeze in the past year (Figure 1). Wheeze in the first year of life was reported in 514 (46%) current participants and a prevalence of 42% (358 children) for those who did not participate in the study at the age of six years (p=0.055). Nine hundred and thirteen (83%) children provided lung function data, and of these 903 (99%) children had their reversibility to salbutamol measured. The mean FEV_1 was 1.13L (standard deviation 0.31), and 331 (37%) had an increase in FEV_1 of more than 10% after administration of aerosolised salbutamol (Figure 2). Two hundred and eighty-three (26%) of the current study population were reported to have received systemic steroids in the previous 12 months (Table 1).

For peer review only - http://bmjopen.bmj.com/site/about/guidelines.xhtml

Risk factors for wheeze in the past 12 months at six years old

The analysis of the association of exposures with wheeze in the past 12 months is presented in Table 2. Both wheeze (odds ratio OR 1.89; 95%CI: 1.65 to 2.16) and paracetamol use (OR 1.64, 95% CI: 1.14 to 2.35) in the first year of life along with a family history of asthma (OR 1.66; 95%CI: 1.40 to 1.97) were associated with wheeze at six years. A positive *Helicobacter pylori* faecal antigen test at age 2 years was negatively associated with wheeze in the past 12 months (OR 0.57; 95%CI: 0.40 to 0.82), but this association was not observed for *Helicobacter pylori* at the age of three years or six years. The number of smokers in the household was a strong risk factor for wheeze in the past 12 months ($p<0.001$ for trend), with homes with two or more smokers having an odds ratio of the child having wheeze in the past 12 months of 2.08 (95% CI: 1.71 to 2.54) compared to those homes with no smokers. The municipality of residence was associated with wheeze in the past 12 months ($p=0.04$, chi^2 test), with children living in Cerro municipality having the highest risk of wheeze (OR 1.72 compared to Arroyo Naranjo; 95%CI: +1.61 to 1.84). These differences were not substantially modified by adjusting for the number of smokers in the home.

Risk factors for decreased FEV_1 at six years old

The analysis of the association of a number of exposures with FEV_1 is presented in Table 3. A number of measures of somatic growth were positively associated with FEV_1 at six years. These were birth height (+14ml per cm height at birth; 95% confidence intervals CI: +6 to +23), current height (+8ml per cm; 95%CI: +2 to +14), current weight (+13ml per Kg; 95%CI: +8 to +17), and current mean arm circumference (+20ml per cm; 95%CI: +14 to +25). The number of smokers living in the home was not associated with lung function, but the municipality of residence was strongly associated with current FEV_1 ($p<0.001$, ANOVA test), with children living in La Lisa having the lowest lung function (-100ml compared to Arroyo Naranjo, 95%CI: -127 to -73). These differences were not substantially modified by adjusting for the number of smokers in the home. No measures of infection or inflammation were associated with lung function.

Risk factors for bronchodilatation after inhaled salbutamol at six years old

The association of exposures with change in FEV_1 from baseline after aerosolised salbutamol was administered is presented in Table 4. Any wheeze in the first year of life was positively associated with bronchodilatation (+1.94%; 95%CI: +0.81 to +3.08) as was a family history of asthma (+1.85; 95%CI: +0.14 to +3.57), but there was no relation with wheeze in the past 12 months (+3.61; 95%CI: -5.80 to 13.02). Children with a higher birth weight had a lower risk of bronchodilatation (–2.67%, 95% CI: -4.49 to -0.84). Higher IgE at the age of one year was associated with a higher risk of bronchodilatation (+1.68%; 95%CI: +0.54 to +2.82), but not IgE at age of three years or six years.

For peer review only - http://bmjopen.bmj.com/site/about/guidelines.xhtml

The numbers of smokers living in the child's home was not associated with bronchodilation, but the municipality of residence was again a strong determinant of current bronchodilatation (p=0.002, ANOVA test), with children living in La Lisa having the highest increase in FEV_1 after administration of inhaled salbutamol at six years old (+6.24% compared to Arroyo Naranjo; 95%CI: +5.56 to +6.91). These differences were not substantially modified by adjusting for the number of smokers in the home.

Discussion

The most striking observation from our cohort study of young children living in Havana is that the prevalence of wheeze in these children was high, with 38% reporting wheeze in the past 12 months. Similarly, there was a high prevalence of bronchoconstriction using the objective measure of lung function reversibility to inhaled salbutamol, with 37% of the study population having an increase in FEV_1 of 10% or more. Over a quarter of these six-year old children have received systemic steroids in the previous 12 months, suggesting that asthma control was suboptimal. The main consistent association is that municipality of residence is a strong risk factor for wheeze, low lung function and bronchoconstriction. A history of wheeze in early life, exposure to paracetamol in the first year of life and current environmental tobacco smoke were risk factors for wheeze, anthropometric measures were positively associated with higher FEV_1, and wheeze in the first year of life and lower birth weight were associated with untreated bronchconstriction.

Strengths and limitations of the data

This is the first cohort study of young children living in Cuba that has collected extensive life course data to evaluate risk factors for asthma using objective measures of lung function and reversibility along with laboratory measures of exposure to infection. The strengths of our data include the prospective nature of the data collected with exposures and outcome measures that were selected to permit exploration of risk factors for asthma specifically within an urban Cuban environment, where the lifestyle, environment and microbial exposures are very different to more temperate, developed countries. The measurement of lung function is a challenge in young children, yet measurements were obtained in 82% of eligible children. The availability of lung function measurements along with reversibility to inhaled salbutamol is a particular strength of these data, as it provides an objective measure that may reflect different aspects of lung health compared to self-reported symptoms.

For peer review only

For peer review only - http://bmjopen.bmj.com/site/about/guidelines.xhtml

Our dataset does have some limitations. We initially recruited 1956 children to the cohort at the age of one year, and approximately five years later were able to collect data on 1106 (57%) of these. This was mainly a consequence of the recent changes in Cuba that have made it easier for the population more mobile, and hence many children left the policlinic from where they were initially recruited when their family moved their residence. There was only a small difference in the prevalence of wheeze in the first year of life between those who provided data at the age of 6 years (46%) and those who did not (42%), so loss to follow-up is unlikely to constitute a major source of bias.

Risk factors for wheeze in the previous 12 months at six years old

Wheeze in the first year of life is associated with wheeze in later childhood [22], and may reflect the establishment of the asthmatic phenotype in susceptible children. The observation that paracetamol consumption in early life is associated with wheeze in later life [23] has been noted before, and our data support these observations. However, they do not demonstrate a causality, as an alternative explanation is reverse causality with the administration of more paracetamol to children who are more susceptible to infections and wheeze [24].

The observation that *Helicobacter pylori* at the age of two years, but not three years old or six years old is associated with lower risk of wheeze at six years old is an interesting observation, that would be consistent with the hypothesis that age of exposure to infection is important. Data from other countries has demonstrated the *Helicobacter* is associated with lower rates of allergic disease but not wheeze in children [11 25], but not in adults [26]. It is striking however that our prevalence of Helicobacter pylori infection was very low at less than 7% in the first six years of life, suggesting that this may be less important in Cuba compared to other countries [11].

From a public health perspective, exposure to cigarette smoking and the municipality of residence are the most important exposures for wheeze at the age of six years. Second-hand cigarette smoke exposure is well recognised as a risk factor for wheeze in children [5], and it is a concern that 51% of the homes in our study population contained at least one smoker. The risk of wheeze was higher in the municipalities of Cerro and La Lisa, which suggest that there may be an environmental factor that predisposes to asthma symptoms such as higher levels of air pollution [27-29].

Risk factors for modified FEV_1 at six years old

The fact that children living in La Lisa municipality already have lower lung function at the age of six years is a major public health concern, and suggests that an exposure associated with living in

For peer review only - http://bmjopen.bmj.com/site/about/guidelines.xhtml

this area may negatively impact on lung growth. La Lisa had by far the highest increase in FEV_1 after administration of aerosolised salbutamol, suggesting that bronchoconstriction is contributing to the low baseline FEV_1 and that a component of the apparent low lung function is reversible. La Lisa is located inland and while there is no obvious sources of excessive industrial pollution [29], and we speculate that this may be associated with higher levels of air pollution as a consequence of the relative absence of sea winds compared to the other municipalities. Weight, height and mean arm circumference were all positively associated with FEV_1 at six years of age which is likely to be simply because they are all measures of somatic growth.

Risk factors for bronchodilatation after inhaled salbutamol at six years old

Wheeze in the first year of life was associated with bronchodilatation after the administration of inhaled salbutamol at six years. This suggests that symptoms of asthma in early life are predictive of untreated bronchoconstriction five years later, and is consistent with the hypothesis that the asthmatic phenotype can be observed to persist from early life onwards [22]. The observation that birth weight is inversely associated with bronchoconstriction provides objective evidence to support the earlier epidemiological evidence that adults with higher birth weights have a lower risk of having a diagnosis of asthma [16]. The observation that a family history asthma is associated with bronchoconstriction also wheeze is consistent with the knowledge that there is a genetic component to asthma [30].

Self-reported wheeze and bronchoconstriction

One interesting observation from our data is that the wheeze in the past 12 months is not associated with either FEV_1, or the change in FEV_1 after administration of inhaled bronchodilator. Although it is possible that children who have had wheeze subsequently received asthma treatment, this lack of association between asthma symptoms and objective measures of asthma has been described previously [31]. This supports that concept that wheeze and reversibility of FEV_1 after administration of aerosolised salbutamol may measure different aspects of lung development and health.

Public health implications of these data

The current study was designed in response to clinical concerns that asthma was becoming a public health problem in Havana. These data support that hypothesis, and in particular identify that living in certain municipalities may result in higher levels of currently unknown exposures that require public health intervention. It is possible that environmental air pollution may drive some of these geographical differences, and that studies of air pollution are urgently required in Havana. The prevalence of exposure to second-hand tobacco smoke in children living in Havana remains

For peer review only - http://bmjopen.bmj.com/site/about/guidelines.xhtml

high, and this is an area where a combination of public health interventions including taxation, legislation that enforces bans of smoking in the workplace and public places, restrictions on tobacco product advertising, and helping individual smokers quit smoking are known to be effective.

Summary

Asthma is common in young children living in Havana, and the high prevalence of the use of systemic steroids probably reflects the underuse of regular inhaled corticosteroid prophylaxis treatments leading to the requirement for rescue treatment for wheezing. As societies urbanise, environmental air pollution may increase from a variety of sources. Cuba's economy has struggled as a consequence of a historical and political events [32], and it remains under an economic embargo from the USA that has negatively impacted on healthcare [33]. This makes providing regular inhaled corticosteroids to all children who need them challenging. However, other low and middle-income countries also have difficult economic and environmental circumstances, and if these observations are replicated elsewhere, then this represents an important global public health issue.

For peer review only - http://bmjopen.bmj.com/site/about/guidelines.xhtml

Acknowledgements

We would like to thank the children and their parents and guardians for participating in this study. We would like to thank all of the staff at the municipalities and policlinics who helped in many ways with data collection and sample analysis.

Contributors: The study was conceived and designed by RSM, SJVF, AWF and JB. Data were collected by RSM, SJVF, VAV, NBSB, CC, MLLG, ZVP, BCT and MABL. Data analysis was conducted by RSM and AWF. RSM, SVF and AWF wrote the first draft of the manuscript. All authors critically reviewed the manuscript, provided intellectual content and approved the final version prior to submission for publication.

Funding: This study was funded by The Wellcome Trust, The University of Nottingham, Nottingham University Hospitals Charity, NIHR Nottingham Biomedical Research Centre and a BMA Award (James Trust).

Competing interests: None declared.

Data availability statement: Cuban regulations do not allow dissemination of national datasets. However, statistical analyses can be performed upon reasonable requests to the corresponding author.

For peer review only

For peer review only - http://bmjopen.bmj.com/site/about/guidelines.xhtml

References

1. Pearce N, Ait-Khaled N, Beasley R, et al. Worldwide trends in the prevalence of asthma symptoms: phase III of the international study of asthma and allergies in childhood (ISAAC). *Thorax* 2007;62:758-66.

2. Jartti T, Gern J. Role of viral infections in the development and exacerbation of asthma in children. *J Allergy Clin Immunology* 2017;140:895-906.

3. Edwards M, Strong K, Cameron A, et al. Viral infections in allergy and immunology: How allergic inflammation influences viral infections and illness. *J Allergy Clin Immunology* 2017;140:909-20.

4. Prescott S. Effects of early cigarette smoke exposure on early immune development and respiratory disease. *Paediatric respiratory reviews* 2008;9:3-10.

5. Lewis S, Antoniak M, Venn A, et al. Secondhand smoke, dietary fruit intake, road traffic exposures, and the prevalence of asthma: A cross-sectional study in young children. *Am J Epidem* 2005;161:406-11.

6. Litonjua A, Gold D. Early-life exposures and later lung function. *Am J Resp Crit Care Med* 2016;193:110-11.

7. Schultz E, Hallberg J, Bellander T, et al. Early-life exposure to traffic-related air pollution and lung function in adolescence. *Am J Resp Crit Care Med* 2016;193:171-77.

8. Sly P, Bush A. From the cradle to the grave: The early-life origins of chronic obstructive pulmonary disease. *Am J Resp Crit Care Med* 2016;193

9. Postma D, Weiss S, den Berge M, et al. Revisiting the dutch hypothesis. *J Allergy Clin Immunology* 2015;136:521-29.

10. Feary J, Britton J, Leonardi-Bee J. Atopy and current intestinal parasite infection: a systematic review and meta-analysis. *Allergy* 2010;66:569-77.

11. Amberbir A, Medhin G, Abegaz W, et al. Exposure to Helicobacter pylori infection in early childhood and the risk of allergic disease and atopic sensitisation: a longitudinal birth cohort study. *Clin Exp Allergy* 2014;44:563-71.

12. Mabalirajan U, Kadhiravan T, Sharma S, et al. TH2 immune response in patients with dengue during defervescence: preliminary evidence. *Am J Trop Med Hygiene* 2005;72:783-85.

13. Fogarty A, Jones S, Britton J, et al. Systemic inflammation and decline in lung function in a general population: a prospective study. *Thorax* 2007;62:515-20.

14. Venero-Fernandez S, Suarez Medina R, Mora Faife E, et al. Risk factors for wheezing in infants born in Cuba. *Quarterly Journal Medicine* 2013;106:1023-29.

15. Suarez-Medina R, Venero-Fernandez S, Mora Faife E, et al. Risk factors for eczema in infants born in Cuba. *BMC Dermatology* 2014;14:6.

For peer review only - http://bmjopen.bmj.com/site/about/guidelines.xhtml

For peer review only

16. Shaheen S, Sterne J, Montgomery S, et al. Birth weight, body mass index and asthma in young adults. *Thorax* 1999;54:396-402.

17. Fundora Hernández H, Suarez-Medina R, Venero Fernandez S, et al. What are the main environmental exposures associated with elevated IgE in Cuban infants? A population-based study. *Tropical Medicine and International Health* 2014;19:545-54.

18. Vircell dengue ELISA IgG. http://wwwperamedcom/peramed/docs/G1018_ENpdf *(accessed 14/4/2016)*

19. Venero-Fernandez S, Fundora-Hernández H, Batista-Gutierrez L, et al. The association of low birth weight with serum C reactive protein in three year old children living in Cuba: A population-based prospective study. *Am J Human Biol* 2016:DOI 10.1002/ajhb.22936.

20. Garcia F, Venero Fernandez S, Fundora Hernández H, et al. Seroprevalencia de anticuerpos IgG anti-Toxoplasma Gondii en infantes de La Habana. *Parasitaria* 2015;73:(in press).

21. Miller M, Hankinson J, Brusasco V, et al. Standardisation of spirometry. *Eur Resp J* 2005;26:319-38.

22. Martinez F, Wright A, Taussig L, et al. Asthma and wheezing in the first six years of life. *New Eng J Med* 1995;332:133-38.

23. Beasley R, Clayton T, Crane J, et al. Association between paracetamol use in infancy and childhood, and risk of asthma, rhinoconjunctivitis, and eczema in children aged 6-7 years: analysis from Phase Three of the ISAAC programnme. *Lancet* 2008;372:1039-48.

24. Rusconi F; Gagliardi L; Galassi C; Forastiere F; Brunetti L; La Grutta S; Piffer S; Talassi F; SIDRIA-2 Collaborative Group. Paracetamol and antibiotics in childhood and subsequent development of wheezing/asthma: association or causation? *Int J Epidemiol* 2011;40:662-67.

25. Amberbir A, Medhin G, Erku W, et al. Effects of Helicobacter pylori, geohelminth infection and selected commensal bacteria on the risk of allergic disease and sensitization in 3 year old Ethiopian children. *Clinical Exp Allergy* 2011;41:1422-30.

26. Fullerton D, Britton J, Lewis S, et al. Helicobacter pylori and lung function, asthma, atopy and allergic disease - A population-based cross-sectional study. *Int J Epidemiol* 2009;38:419-26.

27. Wu W, Jin Y, Carlsten C. Inflammatory health effects of indoor and outdoor particular matter. *J Allergy Clin Immunology* 2018;141:833-44.

28. Gordon S, Bruce N, Grigg J, et al. Respiratory risks from household air pollution in low and middle income countries. *Lancet Respiratory Medicine* 2014;2:823-60.

29. Cuellar-Luna L, Puerto-Rodriguez A, Maldonado-Cantillo G, et al. Distribucion espacial de fuents fijas contaminantes y su impact en salud, provincia La Habana (Cuba). *Hig Sanid Ambient* 2013;13:968-74.

For peer review only - http://bmjopen.bmj.com/site/about/guidelines.xhtml

30. Shrine N, Portelli MA, John C, et al. Moderate-to-severe asthma in individuals of European ancestry: a genome-wide association study. *The Lancet Respiratory medicine* 2019;7(1):20-34. doi: 10.1016/S2213-2600(18)30389-8 [published Online First: 2018/12/16]

31. MacKenney J, Oyarzun M, Diaz P, et al. Prevalence of asthma, atopy and bronchial hyperresponsiveness and their interrelation in a semi-rural area of Chile. *Int J Tuberculosis & Lung Dis* 2005;9:1288-93.

32. Gott R. Cuba. New Haven, USA: Yale University Press 2010.

33. Barry M. Effect of the US embargo and economic decline on health in Cuba. *Ann Intern Med* 2000;132:151-54.

For peer review only

Figure 1. Flow diagram of participants and data collection

Figure 1 goes here

Figure 2. Histogram of percent increase in Forced Expiratory Volume in one second in study population (N=903 children)

Figure 2 goes here

For peer review only

Table 1. Description of study population

	Total (N=1106)	Provided FEV_1 (N=913)
Male sex (%)	575 (52)	473 (52)
Mean age, months (range)	74 (63 to 83)	74 (64 to 83)
Mean FEV_1, L, (sd)	-	1.13 (0.31)
Mean bronchodilation after salbutamol, % (sd)	-	13.4 (20.1) N=903
Wheeze in the past 12 months (%)	422 (38)	356 (39)
**Received inhaled steroids in the 12 months before (%):**		
Year 1	89 (8)	68 (7)
Year 2	176 (17) N=1051	153 (18) N=869
Year 3	203 (18)	163 (18)
Year 4	-	-
Year 5	182 (16)	150 (16)
**Received salbutamol inhaler in the previous 12 months (%):**		
Year 5	416 (38)	345 (38)
**Received IV or oral steroids in the 12 months before (%):**		
Year 1	295 (27)	244 (27)
Year 2	418 (40) N=1051	345 (40) N=869
Year 3	407 (37)	333 (36)
Year 4	-	-
Year 5	283 (26)	240 (26)

For peer review only

For peer review only - http://bmjopen.bmj.com/site/about/guidelines.xhtml

Table 2. Association of exposures with wheeze in past 12 months.

**Total number = 1106**	No (%, sd)	Odds ratio of wheeze (95% CI)
**Prior exposures**		
**Any wheeze in 1st year of life**	514 (46)	**1.89 (1.65 to 2.16)**
**Family history of asthma**	614 (56)	**1.66 (1.40 to 1.97)**
Attendance at nursery	159 (14)	0.84 (0.57 to 1.24)
**Paracetamol in 1st year of life**	256 (23)	**1.64 (1.14 to 2.35)**
Mean birth weight, N=, Kg (sd) N=1104	3.31 (0.51)	0.87 (0.75 to 1.01)
Mean birth height, cm (sd)	50.2 (2.4)	0.95 (0.89 to 1.00)
Breastfeeding ≥ 4months	608 (55)	0.83 (0.66 to 1.03)
Age = 1 year		
Mean log IgE, N=885	+3.38 (1.47)	1.06 (0.88 to 1.27)
Mean log eosinophils, N=856	-2.11(1.05)	0.93 (0.77 to 1.13)
Age = 2 years		
**Helicobacter stool +ve, N=1067**	40 (4)	0.57 (0.40 to 0.82)
Age = 3 years		
Mean log CRP, sd, N=986	-2.07 (2.70)	1.01 (0.95 to 1.07)
Log dengue IgG, N= 865	-0.44 (2.00)	0.99 (0.96 to 1.02)
Helicobacter stool +ve, N=951	58 (6)	1.01 (0.64 to 1.60)
**Mean log eosinophils, N=1039**	-1.77 (1.23)	0.91 (0.86 to 0.96)
Toxoplasmosis serology +ve, N=966	565 (58)	1.15 (0.82 to 1.64)
Mean log IgE, N=986	3.70 (1.53)	1.14 (0.98 to 1.31)
Medical diagnosis of dengue infection	93 (8)	1.40 (0.91 to 2.14)
**Current exposures**		
Male Sex	575 (52)	**1.35 (1.00 to 1.82)**
Age (months), (range)	74 (63 to 83)	**0.97 (0.96 to 0.99)**
Mean current weight, Kg, N=1062	23.3 (11 to 51)	1.01 (0.97 to 1.06)
Current height, cm, N=1057	119 (7)	1.00 (0.97 to 1.03)
Mean arm circumference, cm N=1062	18.3 (2.4)	1.02 (0.95 to 1.08)
Number of current smokers in house		
0	546 (49)	0
1	322 (29)	1.61 (1.22 to 2.12)
≥ 2	238 (22)	2.08 (1.71 to 2.54)
Municipality of residence		p_{TREND}<0.001
**Arroyo Naranjo**	455 (41)	**0**
**Cerro**	139 (13)	**1.72 (1.61 to 1.84)**
**Habana del Este**	307 (28)	**0.86 (0.84 to 0.88)**
**La Lisa**	205 (19)	**1.24 (1.21 to 1.27)**
		**p=0.04**
**Current infection and blood assays**		
Helicobacter stool antigen +ve, N=756	11 (1)	1.45 (0.63 to 3.33)
Toxoplasmosis +ve, N=759	487 (64)	0.97 (0.70 to 1.34)
Mean log dengue IgG serology, N=758	1.67 (1.53)	0.95 (0.90 to 1.01)
Log CRP, N=757	-0.83 (1.41)	0.99 (0.86 to 1.15)
Mean log eosinophils, N=868	-1.62 (1.45)	1.02 (0.90 to 1.15)
**Mean log IgE, N=759**	4.51 (0.97)	1.27 (1.15 to 1.41)
Toxocariasis +ve, N=673	64 (9)	0.96 (0.70 to 1.33)
**Any atopy*** (N=857)	330 (39)	0.88 (0.64 to 1.21)

For peer review only

Table 3. Association of exposures with Forced Expiratory Volume in one second.

**Total number = 913**	No (%, sd)	FEV_1 ml (95% CI)
**Prior exposures**		
Any wheeze in 1st year of life	423 (46)	-17 (-45 to +12)
Family history of asthma	517 (57)	-18 (-79 to +43)
Attendance at nursery	141 (15)	-49 (-155 to +57)
Paracetamol in 1st year of life	216 (24)	+14 (-25 to +52)
Mean birth weight, N=, Kg (sd) N=911	3.32 (0.53)	+60 (-12 to +131)
**Mean birth height, cm (sd)**	**50 (2)**	**+14 (+6 to +23)**
Breastfeeding ≥ 4months	508 (56)	+14 (-71 to +98)
Age = 1 year		
Mean log IgE, N=460	+3.44 (1.39)	-18 (-50 to +13)
Mean log eosinophils, N=454	-2.14 (1.04)	-14 (-52 to +25)
Age = 2 years		
Helicobacter stool +ve, N=593	29 (5)	-72 (-284 to +140)
Age = 3 years		
Mean log CRP, sd, N=622	-2.13 (2.71)	+1 (-12 to +14)
Log dengue IgG, N= 554	-0.43 (2.01)	+5 (-11 to +21)
Helicobacter stool +ve, N=784	52 (7)	-42 (-110 to +25)
Mean log eosinophils, N=659	-1.78 (1.26)	+21 (-3 to +45)
Toxoplasmosis serology +ve, N=614	366 (60)	+50 (-29 to +128)
Mean log IgE, N=622	+3.68 (1.56)	+6 (-21 to +34)
Medical diagnosis of dengue infection	78 (9)	-24 (-59 to +11)
**Current exposures**		
Male Sex	473 (52)	+47 (-11 to +106)
Age (months), (range)	74 (64 to 83)	+7 (-8 to +23)
Wheeze in past 12 months	356 (39)	-60 (-174 to +55)
**Mean weight, Kg, N=903 (range)**	**23.5 (11 to 51)**	**+13 (+8 to +17)**
**Current height, cm, N=903 (range)**	**119 (90 to 175)**	**+8 (+2 to +14)**
**Mean arm circumference, cm N=903**	**18.4 (2.4)**	**+20 (+14 to +25)**
Number of current smokers in house		
0	452 (50)	0
1	264 (29)	+5 (-11 to +22)
≥ 2	197 (22)	-32 (-109 to +46)
Municipality of residence		
**Arroyo Naranjo**	**375 (41)**	**0**
**Cerro**	**120 (13)**	**+61 (+23 to +99)**
**Habana del Este**	**251 (27)**	**+45 (+38 to +53)**
**La Lisa**	**167 (18)**	**-100 (-127 to -73)**
		**P<0.001**
**Current infection and blood assays**		
Helicobacter stool antigen +ve, N=691	10 (1)	-9 (-231 to +213)
Toxoplasmosis +ve, N=694	441 (64)	+3 (-52 to +58)
Mean log dengue IgG serology, N=693	+1.64 (1.55)	-4 (-21 to +14)
Log CRP, N=692	+1.48 (5.73)	-8 (-32 to +17)
Mean log eosinophils, N=791	-1.60 (1.47)	-7 (-43 to +29)
Mean log IgE, N=694	+4.52 (0.97)	-15 (-98 to +67)
Toxocariasis +ve, N=673	60 (9)	-3 (-128 to +121)
Any atopy* (N=826)	316 (38)	+8 (-59 to +76)

adjusted for sex, age in months and clustering by municipality
*defined as any allergen skin prick test > 3mm larger than saline control
FEV_1 = Forced Expiratory Volume in one second
CRP = C Reactive Protein, sd= standard deviation

For peer review only - http://bmjopen.bmj.com/site/about/guidelines.xhtml

Table 4. Association of exposures with bronchodilatation after inhaled salbutamol

**Total number = 903**	No (%, sd)	% change in FEV_1 (95% CI)
**Prior exposures**		
Any wheeze in 1st year of life	417 (46)	**+1.94 (+0.81 to +3.08)**
Family history of asthma	510 (56)	**+1.85 (+0.14 to +3.57)**
Attendance at nursery	138 (15)	-3.21 (-9.10 to +2.68)
Paracetamol in 1st year of life	213 (24)	-0.67 (-4.68 to +3.35)
Mean birth weight, Kg (sd) N=901	3.32 (0.53)	**-2.67 (-4.49 to -0.84)**
Mean birth height, cm	50 (2)	+0.06 (-0.56 to +0.69)
Breastfeeding ≥ 4months	503 (56)	+0.61 (-2.87 to +4.09)
Age = 1 year		
Mean log IgE, N=455	+3.42 (1.39)	**+1.68 (+0.54 to +2.82)**
Mean log eosinophils, N=449	-2.13 (1.04)	-0.16 (-2.54 to +2.21)
Age = 2 years		
Helicobacter stool +ve, N=586	28 (5)	-6.41 (-14.94 to +2.10)
Age = 3 years		
Mean log CRP, sd, N=615	-2.12 (2.71)	-0.28 (-0.88 to +0.32)
Log dengue IgG, N= 550	-0.43 (2.01)	-0.38 (-1.50 to +0.74)
Helicobacter stool +ve, N=776	51 (6)	-1.40 (-7.30 to +4.51)
Mean log eosinophils, N=653	-1.78 (1.26)	-0.39 (-4.45 to +3.68)
Toxoplasmosis serology +ve, N=607	362 (60)	-0.45 (-7.06 to +6.16)
Mean log IgE, N=615	+3.68 (1.57)	+0.23 (-1.81 to +2.27)
Medical diagnosis of dengue infection	78 (9)	-0.58 (-8.55 to +7.38)
**Current exposures**		
Male Sex	466 (52)	+2.22 (-0.28 to +4.71)
Age (months), (range)	74 (64 to 83)	-0.13 (-0.47 to +0.20)
Wheeze in past 12 months	353 (39)	+3.61 (-5.80 to +13.02)
Mean current weight, Kg, N=893 (range)	24 (11 to 51)	-0.23 (-0.54 to +0.08)
Current height, cm, N=893	119 (90 to 175)	-0.08 (-0.50 to +0.34)
Mean arm circumference, cm N=893	18.4 (2.4)	-0.14 (-0.81 to +0.54)
Number of current smokers in house		
0	446 (49)	0
1	263 (29)	-0.87 (-5.89 to +4.14)
≥ 2	194 (21)	+2.93 (-3.80 to +9.65)
Municipality of residence		
Arroyo Naranjo	368 (41)	0
Cerro	119 (13)	**-1.34 (-2.56 to -0.11)**
Habana del Este	249 (28)	**-0.07 (-0.46 to +0.31)**
La Lisa	167 (18)	**+6.24 (+5.56 to +6.91)**
		p=0.002
**Current infection and blood assays**		
Helicobacter stool antigen +ve, N=684	10 (1)	+8.89 (-9.17 to +26.96)
Toxoplasmosis +ve, N=687	438 (64)	-0.47 (-3.34 to +2.39)
Mean log dengue IgG serology, N=686	+1.64 (1.56)	+0.30 (-1.44 to +2.03)
Log CRP, N=685	-0.87 (1.40)	+0.37 (-1.64 to +2.38)
Mean log eosinophils, N=782	-161 1.47)	+0.99 (-0.48 to+2.46)
Mean log IgE, N=686	+4.52 (0.98)	+0.64 (-0.18 to +1.45)
Toxocariasis +ve, N=666	58 (9)	-1.03 (-5.29 to +3.23)
Any atopy* (N=816)	314 (38)	-0.47 (-8.61 to +7.68)

adjusted for sex, age in months and clustering by municipality
*defined as any allergen skin prick test > 3mm larger than saline control
FEV_1 = Forced Expiratory Volume in one second, CRP = C Reactive Protein
sd= standard deviation

For peer review only - http://bmjopen.bmj.com/site/about/guidelines.xhtml

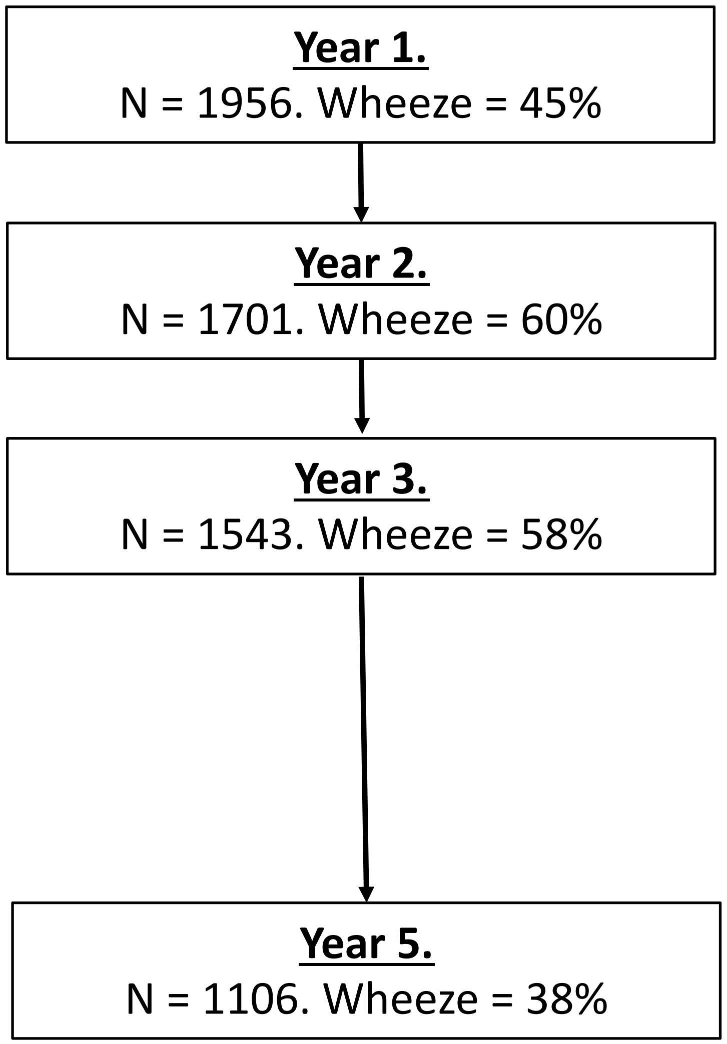
Year 1.
N = 1956. Wheeze = 45%
Year 2.
N = 1701. Wheeze = 60%
Year 3.
N = 1543. Wheeze = 58%
Year 5.
N = 1106. Wheeze = 38%

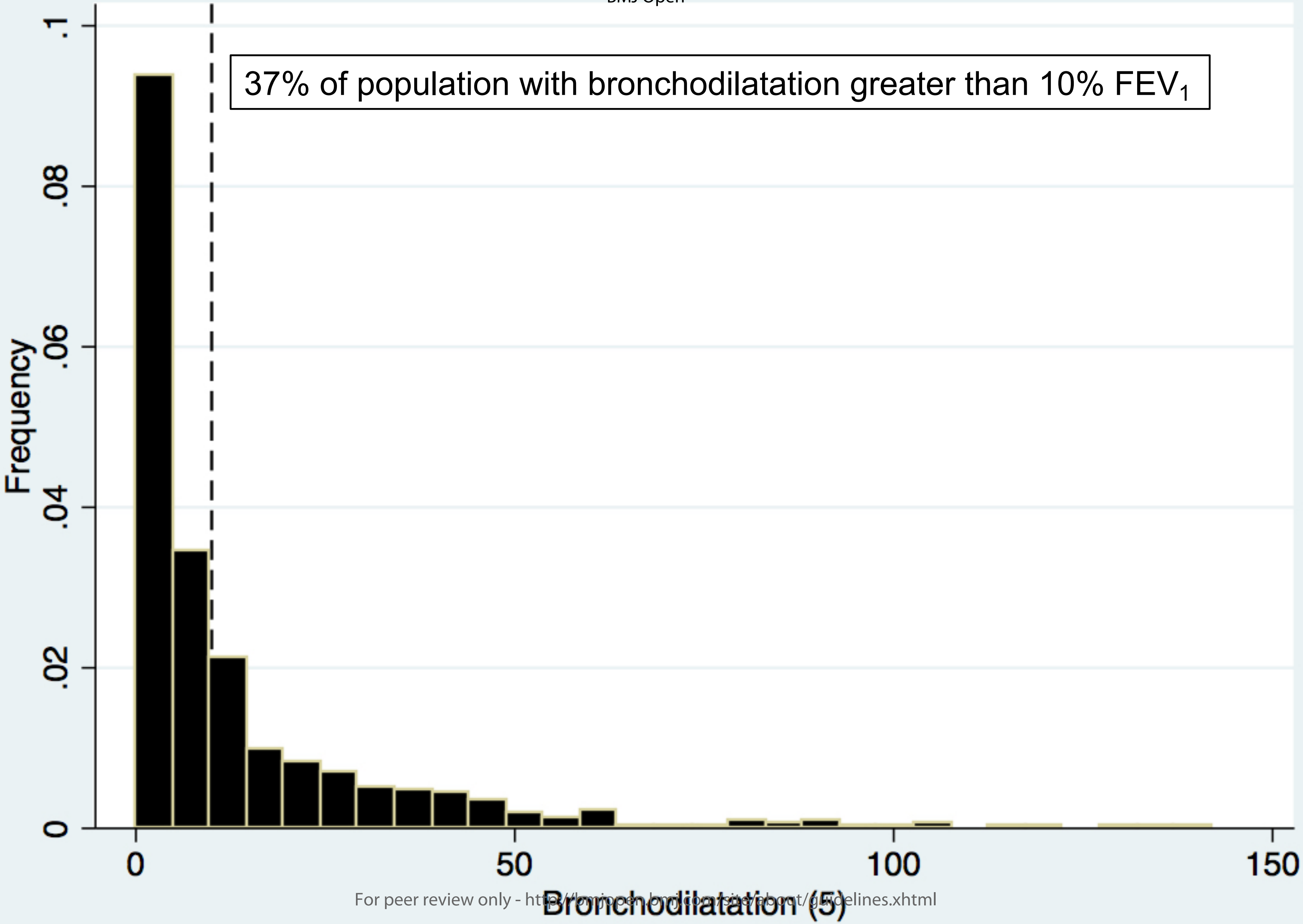
37% of population with bronchodilatation greater than 10% FEV1
Frequency
0
.02
.04
.06
.08
.1
0
50
100
150
Bronchodilatation (5)

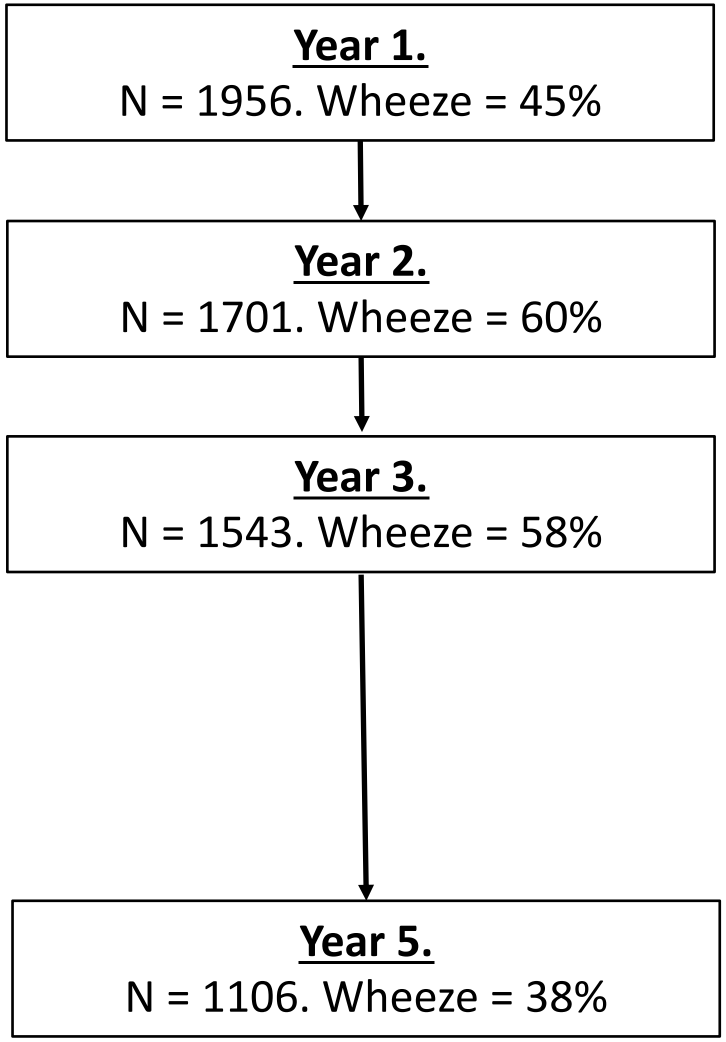
Year 1.
N = 1956. Wheeze = 45%
Year 2.
N = 1701. Wheeze = 60%
Year 3.
N = 1543. Wheeze = 58%
Year 5.
N = 1106. Wheeze = 38%

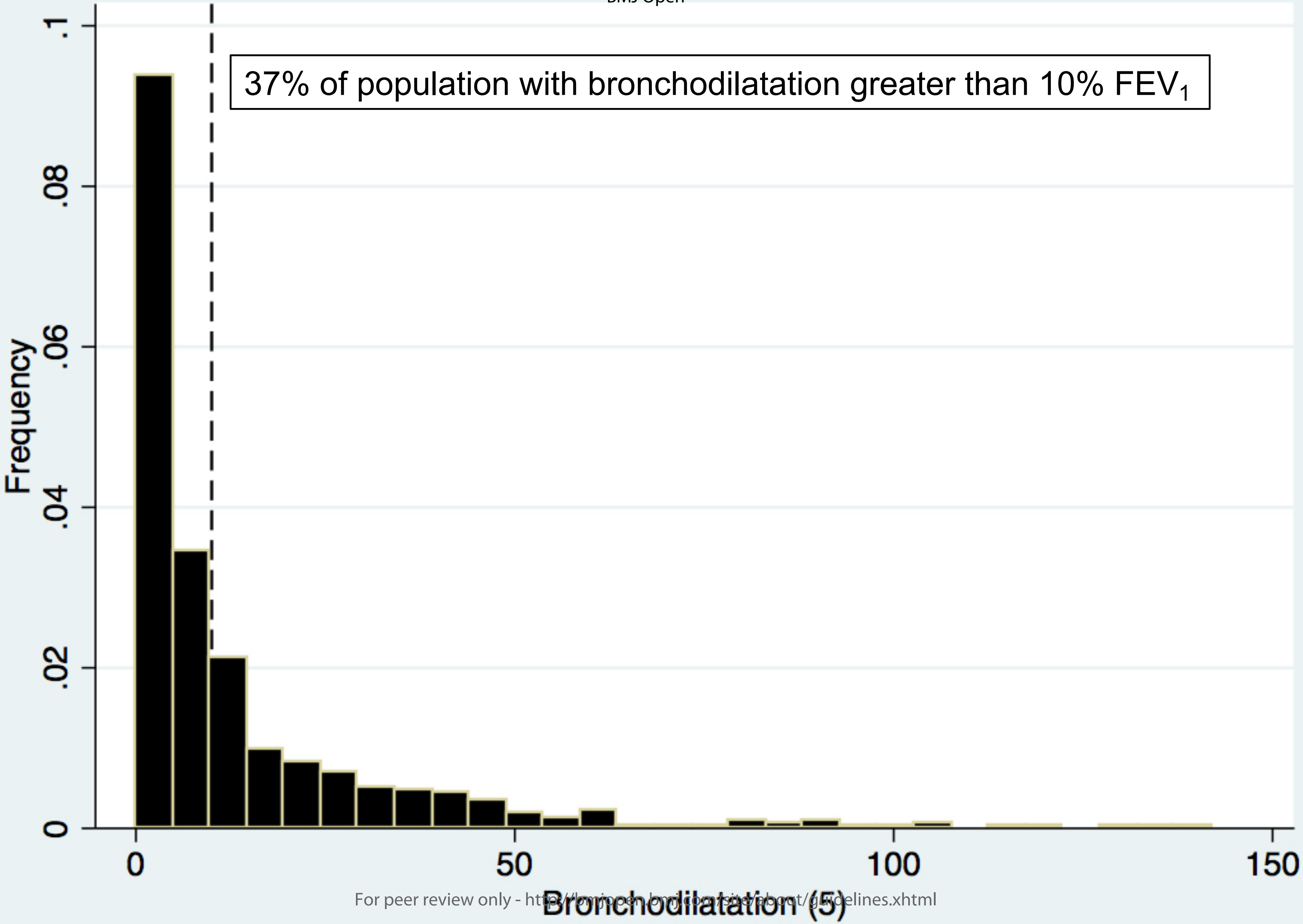
37% of population with bronchodilatation greater than 10% FEV1
Frequency
0
.02
.04
.06
.08
.1
0
50
100
150
Bronchodilatation (5)

STROBE Statement

	Item No	Recommendation
**P1/2 Title and abstract**	1	(*a*) Indicate the study's design with a commonly used term in the title or the abstract
		(*b*) Provide in the abstract an informative and balanced summary of what was done and what was found
**Introduction**		
P4 Background/rationale	2	Explain the scientific background and rationale for the investigation being reported
P4 Objectives	3	State specific objectives, including any prespecified hypotheses
**Methods**		
P4 Study design	4	Present key elements of study design early in the paper
P4 Setting	5	Describe the setting, locations, and relevant dates, including periods of recruitment, exposure, follow-up, and data collection
P4 Participants	6	(*a*) Give the eligibility criteria, and the sources and methods of selection of participants
P5 Variables	7	Clearly define all outcomes, exposures, predictors, potential confounders, and effect modifiers. Give diagnostic criteria, if applicable
P5 Data sources/ measurement	8*	For each variable of interest, give sources of data and details of methods of assessment (measurement). Describe comparability of assessment methods if there is more than one group
P6 Bias	9	Describe any efforts to address potential sources of bias
P4 Study size	10	Explain how the study size was arrived at
P5 Quantitative variables	11	Explain how quantitative variables were handled in the analyses. If applicable, describe which groupings were chosen and why
P5 Statistical methods	12	(*a*) Describe all statistical methods, including those used to control for confounding
		(*b*) Describe any methods used to examine subgroups and interactions
		(*c*) Explain how missing data were addressed
		(*d*) If applicable, describe analytical methods taking account of sampling strategy
		(*e*) Describe any sensitivity analyses
**Results**		
P16 Participants	13*	(a) Report numbers of individuals at each stage of study—eg numbers potentially eligible, examined for eligibility, confirmed eligible, included in the study, completing follow-up, and analysed
		(b) Give reasons for non-participation at each stage
		(c) Consider use of a flow diagram
P18 Descriptive data	14*	(a) Give characteristics of study participants (eg demographic, clinical, social) and information on exposures and potential confounders
		(b) Indicate number of participants with missing data for each variable of interest
P18-20 Outcome data	15*	Report numbers of outcome events or summary measures
P18-20 Main results	16	(*a*) Give unadjusted estimates and, if applicable, confounder-adjusted estimates and their precision (eg, 95% confidence interval). Make clear which confounders were adjusted for and why they were included
		(*b*) Report category boundaries when continuous variables were categorized
		(*c*) If relevant, consider translating estimates of relative risk into absolute risk for a meaningful time period

For peer review only - http://bmjopen.bmj.com/site/about/guidelines.xhtml

P18-20 Other analyses	17	Report other analyses done—eg analyses of subgroups and interactions, and sensitivity analyses
**Discussion**		
P8 Key results	18	Summarise key results with reference to study objectives
P8 Limitations	19	Discuss limitations of the study, taking into account sources of potential bias or imprecision. Discuss both direction and magnitude of any potential bias
P9 Interpretation	20	Give a cautious overall interpretation of results considering objectives, limitations, multiplicity of analyses, results from similar studies, and other relevant evidence
P10 Generalisability	21	Discuss the generalisability (external validity) of the study results
**Other information**		
P12 Funding	22	Give the source of funding and the role of the funders for the present study and, if applicable, for the original study on which the present article is based

*Give information separately for exposed and unexposed groups.

Note: An Explanation and Elaboration article discusses each checklist item and gives methodological background and published examples of transparent reporting. The STROBE checklist is best used in conjunction with this article (freely available on the Web sites of PLoS Medicine at http://www.plosmedicine.org/, Annals of Internal Medicine at http://www.annals.org/, and Epidemiology at http://www.epidem.com/). Information on the STROBE Initiative is available at www.strobe-statement.org.

For peer review only

For peer review only - http://bmjopen.bmj.com/site/about/guidelines.xhtml

BMJ Open

Prevalence and risk factors for wheeze, decreased forced expiratory volume in one second and bronchoconstriction in young children living in Havana, Cuba: A population-based cohort study

Journal:	*BMJ Open*
Manuscript ID	bmjopen-2019-034192.R1
Article Type:	Original research
Date Submitted by the Author:	30-Dec-2019
Complete List of Authors:	Suarez-Medina, Ramon; INHEM<br>Venero-Fernández, Silvia; INHEM<br>Alvarez-Valdés, Vilma; Dirección Municipal de Salud Pública municipios Cerro y Arroyo Naranjo<br>Sardiñas-Baez, Nieves; Dirección Municipal de Salud Pública municipios Cerro y Arroyo Naranjo<br>Cristina, Carmona; Dirección Municipal de Salud Pública municipios Cerro y Arroyo Naranjo<br>Loinaz-Gonzalez, Maria; Dirección Municipal de Salud Pública municipios Cerro y Arroyo Naranjo<br>Verdecia-Pérez, Zunilda; Dirección Municipal de Salud Pública municipios Cerro y Arroyo Naranjo<br>Corona-Tamayo, Barbara; Dirección Municipal de Salud Pública municipios Cerro y Arroyo Naranjo<br>Betancourt-López, Maria; INHEM<br>Britton, John; UK Center for Tobacco and Alcohol Studies, Division of Epidemiology and Public Health<br>Fogarty, Andrew; University of Nottingham,
**Primary Subject Heading**:	Respiratory medicine
Secondary Subject Heading:	Global health, Paediatrics
Keywords:	Asthma < THORACIC MEDICINE, Cuba, PAEDIATRICS

SCHOLARONE™
Manuscripts

I, the Submitting Author has the right to grant and does grant on behalf of all authors of the Work (as defined in the below author licence), an exclusive licence and/or a non-exclusive licence for contributions from authors who are: i) UK Crown employees; ii) where BMJ has agreed a CC-BY licence shall apply, and/or iii) in accordance with the terms applicable for US Federal Government officers or employees acting as part of their official duties; on a worldwide, perpetual, irrevocable, royalty-free basis to BMJ Publishing Group Ltd ("BMJ") its licensees and where the relevant Journal is co-owned by BMJ to the co-owners of the Journal, to publish the Work in this journal and any other BMJ products and to exploit all rights, as set out in our licence.

The Submitting Author accepts and understands that any supply made under these terms is made by BMJ to the Submitting Author unless you are acting as an employee on behalf of your employer or a postgraduate student of an affiliated institution which is paying any applicable article publishing charge ("APC") for Open Access articles. Where the Submitting Author wishes to make the Work available on an Open Access basis (and intends to pay the relevant APC), the terms of reuse of such Open Access shall be governed by a Creative Commons licence – details of these licences and which Creative Commons licence will apply to this Work are set out in our licence referred to above.

Other than as permitted in any relevant BMJ Author's Self Archiving Policies, I confirm this Work has not been accepted for publication elsewhere, is not being considered for publication elsewhere and does not duplicate material already published. I confirm all authors consent to publication of this Work and authorise the granting of this licence.

For peer review only

Prevalence and risk factors for wheeze, decreased forced expiratory volume in one second and bronchoconstriction in young children living in Havana, Cuba: A population-based cohort study

* Ramon Suarez-Medina[1]
Silvia Josefina Venero-Fernández[1]
Vilma Alvarez-Valdés[2]
Nieves B Sardiñas-Baez[2]
Cristina Carmona[2]
María Luisa Loinaz-Gonzalez[2]
Zunilda Verdecia-Pérez[2]
Barbara Corona-Tamayo[2]
María Antonia Betancourt-López[1]
John Britton[3],
Andrew W Fogarty[3]
and the HINASIC (Historia Natural de la Sibilancia en Cuba/Natural History of Wheezing in Cuba) Study Group[++]

[1] Instituto Nacional de Higiene, Epidemiología y Microbiología, Infanta No 1158 e/ Llinás y Clavel, Código Postal 10300, La Habana, Cuba.
[2] Dirección Municipal de Salud Pública municipios Cerro y Arroyo Naranjo, La Habana, Cuba.
[3] Nottingham Biomedical Research Unit, Division of Epidemiology and Public Health, University of Nottingham, Clinical Sciences Building, City Hospital, Nottingham NG5 1PB, UK
[++]full list of collaborating researchers at the end of the manuscript

The corresponding author for the manuscript submission process is Dr Andrew Fogarty (Andrew.fogarty@nottingham.ac.uk) due to poor internet connections with Cuba.

(If accepted for publication) *Correspondence and requests for reprints to Dr. Ramón Suárez Medina:
Address: Infanta No 1158 e/ Llinás y Clavel, Código Postal 10300, La Habana, Cuba.
Office phone: (537) 878 8479
Email: ramonsm@inhem.sld.cu

Key words: Cuba, asthma, children, wheeze

Word count: 3138 words

For peer review only

Abstract (298 words)

Objectives; Asthma has not been extensively studied in low and middle-income countries, where risk factors and access to treatment may differ from more affluent countries. we aimed to identify the prevalence of asthma and local risk factors in Havana, Cuba.

Setting: Four municipalities in Havana, Cuba.

Participants: A population-based cohort study design of young children living in Havana, Cuba. Children were recruited from primary care centres at age 12-15 months.

Primary and secondary outcome measures: Data on wheeze in the past 12 months, asthma treatment and environmental exposures collected regularly until the age of six years, when Forced Expiratory Volume in one second (FEV_1) and reversibility to aerosolised salbutamol were also measured.

Results: 1106 children provided data at the age of six years old. The prevalence of wheeze in the previous 12 months was 422 (38%), and 294 (33%) of the study population had bronchodilatation of 12% or more in FEV_1 after administration of inhaled salbutamol. In the previous 12 months, 182 (16%) of the children had received inhaled corticosteroids, 416 (38%) salbutamol inhalers, and 283 (26%) a course of systemic steroids.

Wheeze in the first year and a family history of asthma were both positively associated with bronchodilatation to inhaled salbutamol (+1.94%; 95% confidence intervals CI: +0.81 to +3.08, and +1.85%; CI: +0.14 to +3.57 respectively), while paracetamol use in the first year was associated with wheeze at six years (OR 1.64, 95% CI: 1.14 to 2.35). There were large differences in FEV_1, bronchodilatation and risk of wheeze across different geographical areas.

Conclusions: Asthma is common in young children living in Havana, and the high prevalence of systemic steroids administrated is likely to reflect the underuse of regular inhaled corticosteroids. If replicated in other comparable low and middle-income countries, this represents an important global public health issue.

Keywords: infection, asthma, children, Cuba, lung function

For peer review only - http://bmjopen.bmj.com/site/about/guidelines.xhtml

STRENGTHS AND LIMITATIONS OF THIS STUDY

- There have been many epidemiological studies of asthma in children who live in developed countries, but few population-based studies that have used objective measures of asthma in developing countries.
- The prevalence of bronchoconstriction in young children living in Havana is high using both subjective and objective measures of asthma.
- These data are from one developing country and thus not generalisable to other nations with different economies and health-care systems.
- The high prevalence of administration of systemic steroids in this population-based sample is public health concern, as it is likely to be avoidable, and may increase the risk of side-effects in later life.

For peer review only

For peer review only - http://bmjopen.bmj.com/site/about/guidelines.xhtml

Introduction

Asthma is a global disease that affects approximately 11% of six year-old children [1], but with marked regional differences in prevalence [1]. The aetiology of asthma is complex, and involves a range of environmental exposures that are likely to have differential impacts at different ages over the human lifetime [2-7]. The early years of childhood is a particularly important period as this is when the lungs and immune system are developing rapidly, and lung function in children is a key determinant of health in adulthood [8].

This study was established as a consequence of concerns among public health specialists and clinicians that asthma was becoming a large problem in Cuba. It aimed to determine the prevalence of wheeze in young children living in Cuba, and to identify modifiable risk factors for wheezing, reduced lung function and reversible bronchoconstriction. The role of infection in early life in the development of allergic disease remains unclear [9]. The main hypothesis of interest was that infection with parasites [10], *Helicobacter pylori* [11], dengue [12], or systemic inflammation [13] may be associated with wheeze or bronchoconstriction. Exposure to environmental tobacco smoke and paracetamol had previously been observed to be positively associated with wheeze [14] or atopic dermatitis [15] symptoms respectively, and so the association of these exposures with wheeze and bronchoconstriction were also studied. Finally, as growth from *in utero* onwards may also be related to development of asthma and growth of the lungs [16], anthropometric measures from birth onwards were also considered. The study design is a prospective population-based study of an existing cohort of children followed from approximately one year of age for five years.

Methods

Study population and sample collection

The study population is a cohort of 1956 children aged 12 to 15 months who were randomly selected from the general population from four municipalities across Havana in 2010 and 2011 [14 15 17]. The response rate of those who were eligible to participate initially was 96% [14]. Data was collected by a standardised questionnaire that was administered by a member of the study team at baseline and subsequently at 2 years, 3 years and 5 years later. This included a number of health and lifestyle questions that were answered by the parent or guardian and particular attention was paid to parental/guardian reported wheeze in the past 12 months using the methodology developed for the ISAAC epidemiological studies of asthma [18], use of asthma medication in the past 12 months and exposure to environmental tobacco smoke. The child's weight, height and mid-arm circumference in both arms were collected at each study visit. Historical baseline data including birth weight and height were collected from the primary care centre records. At each

For peer review only

annual follow-up study the participants' guardians were asked if the child had received a medical diagnosis of dengue infection in the past year (in Cuba all positive diagnoses of dengue infection are clinically confirmed using a serological IgM assay) and a blood sample was collected from children to measure circulating eosinophil levels. This sample was stored at -20 degrees Centigrade and subsequently defrosted and analysed for dengue immunoglobulin G (IgG) serology to generate an antibody index [19], serum immunoglobulin E (IgE) [17], highly sensitive C-Reactive Protein (hsCRP, SpinReact, Spain [20]), toxoplasmosis immunoglobulin G antibodies (IgG) [21] and toxocariasis IgG antibodies (DRG Instruments, Germany). A faecal sample was also collected at each review and stored at minus 20°C, and later examined for *Helicobacter pylori* using the faecal antigen test (SpinReact, Spain) and intestinal parasites using the Kato-Katz test (Campiñas Medical COMI, Brazil).

Lung function

Forced expiratory volume in one second (FEV_1) and Forced Vital Capacity were measured in accordance with ATS/ERS criteria [22] using spirometers (CareFusion Micro I) calibrated each day to allow for local climatic change. The best value of FEV_1 within a threshold of repeatability of 200ml was used as the final value. Aerosolised salbutamol (300 μg) was then administered via a spacer and after 15 minutes lung function was measured again to quantify airway reversibility. In children who provided a post-bronchodilator FEV_1 that was less than the baseline value, they were considered as having no reversibility to bronchodilator as this was likely to be due to fatigue.

Allergen skin prick testing

Skin prick testing was used to determine allergy to mite, cat, grass, cockroach, fungus, mosquitos, wheat and soy (allergens from Diater, Argentina except mite allergen from Biocen, Cuba). For each test a drop of allergen solution was placed on the skin and a lancet used to break the skin. After 15 minutes, the skin wheal was measured at its maximum diameter, and also perpendicularly, and a mean value generated. The final skin prick test result was calculated by subtracting the saline result from the allergen. A value of ≥3mm was used to define a positive atopic result for each allergen, and atopy was defined as any positive skin prick test.

Statistical analysis

The main outcome variables were FEV_1, percent increase in FEV_1 after to inhaled salbutamol and wheeze in the past 12 months. The main exposure variables were grouped into three categories:

1. Prior exposures: wheeze in the first year of life, family history of asthma, nursery attendance, birth weight, birth height, duration of breastfeeding, blood IgE and eosinophils at one year old; faecal *Helicobacter pylori* antigen at two and three years old; blood hsCRP,

For peer review only - http://bmjopen.bmj.com/site/about/guidelines.xhtml

dengue IgG serology, eosinophils, toxoplasmosis serology, IgE at three years old, and any prior medical diagnosis of dengue infection.

2. Cross-section exposures: number of smokers living in the home, current weight, current height, mean arm circumference, municipality of residence.
3. Biomarkers of current infection and inflammation: Helicobacter pylori stool antigen, Toxoplasmosis IgG serology, dengue IgG serology, blood hsCRP, eosinophils, IgE, toxocariasis serology and atopy. Less than 2% of children has current gastro-intestinal parasite infection and these data were not analysed further.

Statistical analysis used linear and logistic regression adjusting for sex and age in months as *a priori* confounding factors, and also adjusted for clustering by municipality of residence. All analyses used Stata 14 statistical software (Texas, USA).

Patient and Public Involvement

The study was designed as a consequence of concerns from the Cuban public health and clinical communities about asthma morbidity. The patients were not involved in the design of the study and patients did not receive a copy of the results. We thank the patients and their families for their participation.

Ethics approval

The study was approved by Ethics Committees in the Instituto Nacional de Higiene, Epidemiología y Microbiología, Cuba and at the University of Nottingham, UK. This involved parental consent on the behalf of the child.

Results

Data were available for 1106 children, of whom 422 (38%) had reported wheeze in the past year (Figure 1). Wheeze in the first year of life was reported in 514 (46%) current participants, while there was a prevalence of wheeze in the first year of life of 42% (358 children) for those who did not participate in the study at the age of six years (p=0.055). Nine hundred and thirteen (83%) children provided lung function data, and of these 903 (99%) children had their reversibility to salbutamol measured. The mean FEV_1 was 1.13L (standard deviation 0.31), and 294 (33%) had an increase in FEV_1 of more than 12% after administration of aerosolised salbutamol (Figure 2). Two hundred and eighty-three (26%) of the current study population were reported to have received systemic steroids in the previous 12 months (Table 1).

For peer review only - http://bmjopen.bmj.com/site/about/guidelines.xhtml

Risk factors for wheeze in the past 12 months at six years old

The analysis of the association of exposures with wheeze in the past 12 months is presented in Table 2. Both wheeze (odds ratio OR 1.89; 95%CI: 1.65 to 2.16) and paracetamol use (OR 1.64, 95% CI: 1.14 to 2.35) in the first year of life along with a family history of asthma (OR 1.66; 95%CI: 1.40 to 1.97) were associated with wheeze at six years. A positive *Helicobacter pylori* faecal antigen test at age 2 years was negatively associated with wheeze in the past 12 months (OR 0.57; 95%CI: 0.40 to 0.82), but this association was not observed for *Helicobacter pylori* at the age of three years or six years. The number of smokers in the household was a strong risk factor for wheeze in the past 12 months ($p<0.001$ for trend), with homes with two or more smokers having an odds ratio of the child having wheeze in the past 12 months of 2.08 (95% CI: 1.71 to 2.54) compared to those homes with no smokers. The municipality of residence was associated with wheeze in the past 12 months ($p=0.04$, chi^2 test), with children living in Cerro municipality having the highest risk of wheeze (OR 1.72 compared to Arroyo Naranjo; 95%CI: +1.61 to 1.84). These differences were not substantially modified by adjusting for the number of smokers in the home.

Risk factors for decreased FEV_1 at six years old

The analysis of the association of a number of exposures with FEV_1 is presented in Table 3. A number of measures of somatic growth were positively associated with FEV_1 at six years. These were birth height (+14ml per cm height at birth; 95% confidence intervals CI: +6 to +23), current height (+8ml per cm; 95%CI: +2 to +14), current weight (+13ml per Kg; 95%CI: +8 to +17), and current mean arm circumference (+20ml per cm; 95%CI: +14 to +25). The number of smokers living in the home was not associated with lung function, but the municipality of residence was strongly associated with current FEV_1 ($p<0.001$, ANOVA test), with children living in La Lisa having the lowest lung function (-100ml compared to Arroyo Naranjo, 95%CI: -127 to -73). These differences were not substantially modified by adjusting for the number of smokers in the home. No measures of infection or inflammation were associated with lung function.

Risk factors for bronchodilatation after inhaled salbutamol at six years old

The association of exposures with change in FEV_1 from baseline after aerosolised salbutamol was administered is presented in Table 4. Any wheeze in the first year of life was positively associated with bronchodilatation (+1.94%; 95%CI: +0.81 to +3.08) as was a family history of asthma (+1.85; 95%CI: +0.14 to +3.57), but there was no relation with wheeze in the past 12 months (+3.61; 95%CI: -5.80 to 13.02). Children with a higher birth weight had a lower risk of bronchodilatation

For peer review only - http://bmjopen.bmj.com/site/about/guidelines.xhtml

(–2.67%, 95% CI: -4.49 to -0.84). IgE at the age of one year was positively associated with a higher risk of bronchodilatation (+1.68%; 95%CI: +0.54 to +2.82), but not IgE at age of three years or six years.

The numbers of smokers living in the child's home was not associated with bronchodilation, but the municipality of residence was again a strong determinant of current bronchodilatation (p=0.002, ANOVA test), with children living in La Lisa having the highest increase in FEV_1 after administration of inhaled salbutamol at six years old (+6.24% compared to Arroyo Naranjo; 95%CI: +5.56 to +6.91). These differences were not substantially modified by adjusting for the number of smokers in the home.

Discussion

The most striking observation from our cohort study of young children living in Havana is that the prevalence of wheeze in these children was high, with 38% reporting wheeze in the past 12 months. Similarly, there was a high prevalence of bronchoconstriction using the objective measure of lung function reversibility to inhaled salbutamol, with 33% of the study population having an increase in FEV_1 of 12% or more. Over a quarter of these six-year old children have received systemic steroids in the previous 12 months, suggesting that asthma control was suboptimal. The main consistent association is that municipality of residence is a strong risk factor for wheeze, low lung function and bronchoconstriction. A history of wheeze in early life, exposure to paracetamol in the first year of life and current environmental tobacco smoke were risk factors for wheeze, anthropometric measures were positively associated with higher FEV_1, and wheeze in the first year of life and lower birth weight were associated with untreated bronchconstriction.

Strengths and limitations of the data

This is the first cohort study of young children living in Cuba that has collected extensive life course data to evaluate risk factors for asthma using objective measures of lung function and reversibility along with laboratory measures of exposure to infection. The strengths of our data include the prospective nature of the data collected with exposures and outcome measures that were selected to permit exploration of risk factors for asthma specifically within an urban Cuban environment, where the lifestyle, environment and microbial exposures are very different to more temperate, developed countries. The measurement of lung function is a challenge in young children, yet measurements were obtained in 82% of eligible children. The availability of lung function measurements along with reversibility to inhaled salbutamol is a particular strength of

For peer review only - http://bmjopen.bmj.com/site/about/guidelines.xhtml
For peer review only

these data, as it provides an objective measure that may reflect different aspects of lung health compared to self-reported symptoms.

Our dataset does have some limitations. We initially recruited 1956 children to the cohort at the age of one year with a response rate of 96% eligible children, and approximately five years later were able to collect data on 1106 (57%) of these. This was mainly a consequence of the recent changes in Cuba that have made it easier for the population more mobile, and hence many children left the policlinic from where they were initially recruited when their family moved their residence. There was only a small difference in the prevalence of wheeze in the first year of life between those who provided data at the age of 6 years (46%) and those who did not (42%), so loss to follow-up is unlikely to constitute a major source of bias. Collecting data on parental or self-reported wheeze using questionnaires is challenging, and although we used the optimal methodology from the ISAAC international studies of allergic disease [18] it is possible that some of the cases of wheeze were not asthma. Similarly, parental reported asthma medication use may be prone to recall bias, although as acute asthma in children is an upsetting family event it is likely to be remembered accurately although the time period possibly less so. This included a number of health and lifestyle questions that were answered by the parent or guardian and particular attention was paid to parental/guardian reported wheeze in the past 12 months using the methodology developed for the ISAAC epidemiological studies of asthma [18], use of asthma medication in the past 12 months and exposure to environmental tobacco smoke. There are no validated lung function normal values in Cuban children aged 6 years old and as a consequence we were unable to generate reliable percent predicted values of the lung function in our study population.

Like many cohorts, our study began as a cross-sectional population-based that aimed to explore causes of the high prevalence of asthma in Habana, Cuba. Further funding permitted this to become a prospective study, with a particular emphasis on infections found in the tropics. As a consequence we have tested a variety of analyses and present the results in their entirety. While we recognise that it is possible to generate a number of papers analysing different hypotheses from these data, this approach has the advantage of presenting a complete body of work that hopefully can inform researchers who are interested in this area.

Risk factors for wheeze in the previous 12 months at six years old

Wheeze in the first year of life is associated with wheeze in later childhood [23], and may reflect the establishment of the asthmatic phenotype in susceptible children. The observation that paracetamol consumption in early life is associated with wheeze in later life [24] has been noted

For peer review only - http://bmjopen.bmj.com/site/about/guidelines.xhtml

before, and our data support these observations. However, they do not demonstrate a causality, as an alternative explanation is reverse causality with the administration of more paracetamol to children who are more susceptible to infections and wheeze [25].

The observation that *Helicobacter pylori* at the age of two years, but not three years old or six years old is associated with lower risk of wheeze at six years old is an interesting observation, that would be consistent with the hypothesis that age of exposure to infection is important. Data from other countries has demonstrated the *Helicobacter* is associated with lower rates of allergic disease but not wheeze in children [11 26], but not in adults [27]. It is striking however that our prevalence of Helicobacter pylori infection was very low at less than 7% in the first six years of life, suggesting that this may be less important in Cuba compared to other countries [11].

From a public health perspective, exposure to cigarette smoking and the municipality of residence are the most important exposures for wheeze at the age of six years. Second-hand cigarette smoke exposure is well recognised as a risk factor for wheeze in children [5], and it is a concern that 51% of the homes in our study population contained at least one smoker. The risk of wheeze was higher in the municipalities of Cerro and La Lisa, which suggest that there may be an environmental factor that predisposes to asthma symptoms such as higher levels of air pollution [28-30].

Risk factors for modified FEV_1 at six years old

The fact that children living in La Lisa municipality already have lower lung function at the age of six years is a major public health concern, and suggests that an exposure associated with living in this area may negatively impact on lung growth. La Lisa had by far the highest increase in FEV_1 after administration of aerosolised salbutamol, suggesting that bronchoconstriction is contributing to the low baseline FEV_1 and that a component of the apparent low lung function is reversible. La Lisa is located inland and while there is no obvious sources of excessive industrial pollution [30], and we speculate that this may be associated with higher levels of air pollution as a consequence of the relative absence of sea winds compared to the other municipalities. Weight, height and mean arm circumference were all positively associated with FEV_1 at six years of age which is likely to be simply because they are all measures of somatic growth.

Risk factors for bronchodilatation after inhaled salbutamol at six years old

Wheeze in the first year of life was associated with bronchodilatation after the administration of inhaled salbutamol at six years. This suggests that symptoms of asthma in early life are predictive of untreated bronchoconstriction five years later, and is consistent with the hypothesis that the

For peer review only - http://bmjopen.bmj.com/site/about/guidelines.xhtml

asthmatic phenotype can be observed to persist from early life onwards [23]. The observation that birth weight is inversely associated with bronchoconstriction provides objective evidence to support the earlier epidemiological evidence that adults with higher birth weights have a lower risk of having a diagnosis of asthma [16]. The observation that a family history asthma is associated with bronchoconstriction also wheeze is consistent with the knowledge that there is a genetic component to asthma [31].

Self-reported wheeze and bronchoconstriction

One interesting observation from our data is that the wheeze in the past 12 months is not associated with either FEV_1, or the change in FEV_1 after administration of inhaled bronchodilator. Although it is possible that children who have had wheeze subsequently received asthma treatment, this lack of association between asthma symptoms and objective measures of asthma has been described previously [32]. This supports that concept that wheeze and reversibility of FEV_1 after administration of aerosolised salbutamol may measure different aspects of lung development and health.

Public health implications of these data

The current study was designed in response to clinical concerns that asthma was becoming a public health problem in Havana. These data support that hypothesis, and in particular identify that living in certain municipalities may result in higher levels of currently unknown exposures that require public health intervention. It is possible that environmental air pollution may drive some of these geographical differences, and that studies of air pollution are urgently required in Havana. The prevalence of exposure to second-hand tobacco smoke in children living in Havana remains high, and this is an area where a combination of public health interventions including taxation, legislation that enforces bans of smoking in the workplace and public places, restrictions on tobacco product advertising, and helping individual smokers quit smoking are known to be effective. We are exploring these hypotheses further by doing further objective measurements of particulate air pollution within the study population.

Summary

Asthma is common in young children living in Havana, and the high prevalence of the use of systemic steroids probably reflects the underuse of regular inhaled corticosteroid prophylaxis treatments leading to the requirement for rescue treatment for wheezing. As societies urbanise, environmental air pollution may increase from a variety of sources. Cuba's economy has struggled as a consequence of a historical and political events [33], and it remains under an economic embargo from the USA that has negatively impacted on healthcare [34]. This makes providing

For peer review only - http://bmjopen.bmj.com/site/about/guidelines.xhtml

regular inhaled corticosteroids to all children who need them challenging. However, other low and middle-income countries also have difficult economic and environmental circumstances, and if these observations are replicated elsewhere, then this represents an important global public health issue.

For peer review only

For peer review only - http://bmjopen.bmj.com/site/about/guidelines.xhtml

Acknowledgements

We would like to thank the children and their parents and guardians for participating in this study. We would like to thank all of the staff at the municipalities and policlinics who helped in many ways with data collection and sample analysis.

Contributors: The study was conceived and designed by RSM, SVF, AWF and JB. Data were collected by RSM, SVF, VAV, NBSB, CC, MLLG, ZVP, BCT and MABL. Data analysis was conducted by RSM and AWF. RSM, SVF and AWF wrote the first draft of the manuscript. All authors critically reviewed the manuscript, provided intellectual content and approved the final version prior to submission for publication.

Funding: This study was funded by The Wellcome Trust, The University of Nottingham, Nottingham University Hospitals Charity, NIHR Nottingham Biomedical Research Centre and a BMA Award (James Trust).

Competing interests: None declared.

Data sharing statement: Cuban regulations do not allow dissemination of national datasets. However, statistical analyses can be performed upon reasonable requests to the corresponding author.

For peer review only

References

1. Pearce N, Ait-Khaled N, Beasley R, et al. Worldwide trends in the prevalence of asthma symptoms: phase III of the international study of asthma and allergies in childhood (ISAAC). *Thorax* 2007;62:758-66.
2. Jartti T, Gern J. Role of viral infections in the development and exacerbation of asthma in children. *J Allergy Clin Immunology* 2017;140:895-906.
3. Edwards M, Strong K, Cameron A, et al. Viral infections in allergy and immunology: How allergic inflammation influences viral infections and illness. *J Allergy Clin Immunology* 2017;140:909-20.
4. Prescott S. Effects of early cigarette smoke exposure on early immune development and respiratory disease. *Paediatric respiratory reviews* 2008;9:3-10.
5. Lewis S, Antoniak M, Venn A, et al. Secondhand smoke, dietary fruit intake, road traffic exposures, and the prevalence of asthma: A cross-sectional study in young children. *Am J Epidem* 2005;161:406-11.
6. Litonjua A, Gold D. Early-life exposures and later lung function. *Am J Resp Crit Care Med* 2016;193:110-11.
7. Schultz E, Hallberg J, Bellander T, et al. Early-life exposure to traffic-related air pollution and lung function in adolescence. *Am J Resp Crit Care Med* 2016;193:171-77.
8. Sly P, Bush A. From the cradle to the grave: The early-life origins of chronic obstructive pulmonary disease. *Am J Resp Crit Care Med* 2016;193
9. Postma D, Weiss S, den Berge M, et al. Revisiting the dutch hypothesis. *J Allergy Clin Immunology* 2015;136:521-29.
10. Feary J, Britton J, Leonardi-Bee J. Atopy and current intestinal parasite infection: a systematic review and meta-analysis. *Allergy* 2010;66:569-77.
11. Amberbir A, Medhin G, Abegaz W, et al. Exposure to Helicobacter pylori infection in early childhood and the risk of allergic disease and atopic sensitisation: a longitudinal birth cohort study. *Clin Exp Allergy* 2014;44:563-71.
12. Mabalirajan U, Kadhiravan T, Sharma S, et al. TH2 immune response in patients with dengue during defervescence: preliminary evidence. *Am J Trop Med Hygiene* 2005;72:783-85.
13. Fogarty A, Jones S, Britton J, et al. Systemic inflammation and decline in lung function in a general population: a prospective study. *Thorax* 2007;62:515-20.
14. Venero-Fernandez S, Suarez Medina R, Mora Faife E, et al. Risk factors for wheezing in infants born in Cuba. *Quarterly Journal Medicine* 2013;106:1023-29.
15. Suarez-Medina R, Venero-Fernandez S, Mora Faife E, et al. Risk factors for eczema in infants born in Cuba. *BMC Dermatology* 2014;14:6.
16. Shaheen S, Sterne J, Montgomery S, et al. Birth weight, body mass index and asthma in young adults. *Thorax* 1999;54:396-402.
17. Fundora Hernández H, Suarez-Medina R, Venero Fernandez S, et al. What are the main environmental exposures associated with elevated IgE in Cuban infants? A population-based study. *Tropical Medicine and International Health* 2014;19:545-54.
18. Committee TISoAaAiCIS. Worldwide variations in the prevalence of asthma symptoms: the International Study of Asthma and Allergies in Childhood (ISAAC) Steering Committee. *Eur Resp J* 1998;12:315-35.
19. Vircell dengue ELISA IgG. *http://wwwperamedcom/peramed/docs/G1018_ENpdf (accessed 14/4/2016)*
20. Venero-Fernandez S, Fundora-Hernández H, Batista-Gutierrez L, et al. The association of low birth weight with serum C reactive protein in three year old children living in Cuba: A population-based prospective study. *Am J Human Biol* 2016:DOI 10.1002/ajhb.22936.
21. Garcia F, Venero Fernandez S, Fundora Hernández H, et al. Seroprevalencia de anticuerpos IgG anti-Toxoplasma Gondii en infantes de La Habana. *Parasitaria* 2015;73:(in press).
22. Miller M, Hankinson J, Brusasco V, et al. Standardisation of spirometry. *Eur Resp J* 2005;26:319-38.
23. Martinez F, Wright A, Taussig L, et al. Asthma and wheezing in the first six years of life. *New Eng J Med* 1995;332:133-38.

For peer review only - http://bmjopen.bmj.com/site/about/guidelines.xhtml

24. Beasley R, Clayton T, Crane J, et al. Association between paracetamol use in infancy and childhood, and risk of asthma, rhinoconjunctivitis, and eczema in children aged 6-7 years: analysis from Phase Three of the ISAAC progranmme. *Lancet* 2008;372:1039-48.
25. Rusconi F; Gagliardi L; Galassi C; Forastiere F; Brunetti L; La Grutta S; Piffer S; Talassi F; SIDRIA-2 Collaborative Group. Paracetamol and antibiotics in childhood and subsequent development of wheezing/asthma: association or causation? *Int J Epidemiol* 2011;40:662-67.
26. Amberbir A, Medhin G, Erku W, et al. Effects of Helicobacter pylori, geohelminth infection and selected commensal bacteria on the risk of allergic disease and sensitization in 3 year old Ethiopian children. *Clinical Exp Allergy* 2011;41:1422-30.
27. Fullerton D, Britton J, Lewis S, et al. Helicobacter pylori and lung function, asthma, atopy and allergic disease - A population-based cross-sectional study. *Int J Epidemiol* 2009;38:419-26.
28. Wu W, Jin Y, Carlsten C. Inflammatory health effects of indoor and outdoor particular matter. *J Allergy Clin Immunology* 2018;141:833-44.
29. Gordon S, Bruce N, Grigg J, et al. Respiratory risks from household air pollution in low and middle income countries. *Lancet Respiratory Medicine* 2014;2:823-60.
30. Cuellar-Luna L, Puerto-Rodriguez A, Maldonado-Cantillo G, et al. Distribucion espacial de fuents fijas contaminantes y su impact en salud, provincia La Habana (Cuba). *Hig Sanid Ambient* 2013;13:968-74.
31. Shrine N, Portelli MA, John C, et al. Moderate-to-severe asthma in individuals of European ancestry: a genome-wide association study. *The Lancet Respiratory medicine* 2019;7(1):20-34. doi: 10.1016/S2213-2600(18)30389-8 [published Online First: 2018/12/16]
32. MacKenney J, Oyarzun M, Diaz P, et al. Prevalence of asthma, atopy and bronchial hyperresponsiveness and their interrelation in a semi-rural area of Chile. *Int J Tuberculosis & Lung Dis* 2005;9:1288-93.
33. Gott R. Cuba. New Haven, USA: Yale University Press 2010.
34. Barry M. Effect of the US embargo and economic decline on health in Cuba. *Ann Intern Med* 2000;132:151-54.

For peer review only

For peer review only - http://bmjopen.bmj.com/site/about/guidelines.xhtml

Figure 1. Flow diagram of participants and data collection

Figure 1 goes here

Figure 2. Histogram of percent increase in Forced Expiratory Volume in one second in study population (N=903 children)

Figure 2 goes here

For peer review only

For peer review only - http://bmjopen.bmj.com/site/about/guidelines.xhtml

Table 1. Description of study population

	Total (N=1106)	Provided FEV_1 (N=913)
Male sex (%)	575 (52)	473 (52)
Mean age, months (range)	74 (63 to 83)	74 (64 to 83)
Mean FEV_1, L, (sd)	-	1.13 (0.31)
Mean bronchodilation after salbutamol, % (sd)	-	13.4 (20.1) N=903
Wheeze in the past 12 months (%)	422 (38)	356 (39)
**Received inhaled steroids in the 12 months before (%):**		
Year 1	89 (8)	68 (7)
Year 2	176 (17) N=1051	153 (18) N=869
Year 3	203 (18)	163 (18)
Year 4	-	-
Year 5	182 (16)	150 (16)
**Received salbutamol inhaler in the previous 12 months (%):**		
Year 5	416 (38)	345 (38)
**Received IV or oral steroids in the 12 months before (%):**		
Year 1	295 (27)	244 (27)
Year 2	418 (40) N=1051	345 (40) N=869
Year 3	407 (37)	333 (36)
Year 4	-	-
Year 5	283 (26)	240 (26)

For peer review only

For peer review only - http://bmjopen.bmj.com/site/about/guidelines.xhtml

Table 2. Association of exposures with wheeze in past 12 months.

**Total number = 1106**	No (%, sd)	Odds ratio of wheeze (95% CI)
**Prior exposures**		
**Any wheeze in 1st year of life**	514 (46)	**1.89 (1.65 to 2.16)**
**Family history of asthma**	614 (56)	**1.66 (1.40 to 1.97)**
Attendance at nursery	159 (14)	0.84 (0.57 to 1.24)
**Paracetamol in 1st year of life**	256 (23)	**1.64 (1.14 to 2.35)**
Mean birth weight, N=, Kg (sd) N=1104	3.31 (0.51)	0.87 (0.75 to 1.01)
Mean birth height, cm (sd)	50.2 (2.4)	0.95 (0.89 to 1.00)
Breastfeeding ≥ 4months	608 (55)	0.83 (0.66 to 1.03)
Age = 1 year		
Mean log IgE, N=885	+3.38 (1.47)	1.06 (0.88 to 1.27)
Mean log eosinophils, N=856	-2.11(1.05)	0.93 (0.77 to 1.13)
Age = 2 years		
**Helicobacter stool +ve, N=1067**	40 (4)	0.57 (0.40 to 0.82)
Age = 3 years		
Mean log CRP, sd, N=986	-2.07 (2.70)	1.01 (0.95 to 1.07)
Log dengue IgG, N= 865	-0.44 (2.00)	0.99 (0.96 to 1.02)
Helicobacter stool +ve, N=951	58 (6)	1.01 (0.64 to 1.60)
**Mean log eosinophils, N=1039**	-1.77 (1.23)	0.91 (0.86 to 0.96)
Toxoplasmosis serology +ve, N=966	565 (58)	1.15 (0.82 to 1.64)
Mean log IgE, N=986	3.70 (1.53)	1.14 (0.98 to 1.31)
Medical diagnosis of dengue infection	93 (8)	1.40 (0.91 to 2.14)
**Current exposures**		
Male Sex	575 (52)	**1.35 (1.00 to 1.82)**
Age (months), (range)	74 (63 to 83)	**0.97 (0.96 to 0.99)**
Mean current weight, Kg, N=1062	23.3 (11 to 51)	1.01 (0.97 to 1.06)
Current height, cm, N=1057	119 (7)	1.00 (0.97 to 1.03)
Mean arm circumference, cm N=1062	18.3 (2.4)	1.02 (0.95 to 1.08)
Number of current smokers in house		
0	546 (49)	0
1	322 (29)	1.61 (1.22 to 2.12)
**≥ 2**	238 (22)	2.08 (1.71 to 2.54)
Municipality of residence		p_{TREND}<0.001
**Arroyo Naranjo**	455 (41)	**0**
**Cerro**	139 (13)	**1.72 (1.61 to 1.84)**
**Habana del Este**	307 (28)	**0.86 (0.84 to 0.88)**
**La Lisa**	205 (19)	**1.24 (1.21 to 1.27)**
		**p=0.04**
**Current infection and blood assays**		
Helicobacter stool antigen +ve, N=756	11 (1)	1.45 (0.63 to 3.33)
Toxoplasmosis +ve, N=759	487 (64)	0.97 (0.70 to 1.34)
Mean log dengue IgG serology, N=758	1.67 (1.53)	0.95 (0.90 to 1.01)
Log CRP, N=757	-0.83 (1.41)	0.99 (0.86 to 1.15)
Mean log eosinophils, N=868	-1.62 (1.45)	1.02 (0.90 to 1.15)
**Mean log IgE, N=759**	4.51 (0.97)	1.27 (1.15 to 1.41)
Toxocariasis +ve, N=673	64 (9)	0.96 (0.70 to 1.33)
**Any atopy*** (N=857)	330 (39)	0.88 (0.64 to 1.21)

For peer review only

For peer review only - http://bmjopen.bmj.com/site/about/guidelines.xhtml

Table 3. Association of exposures with Forced Expiratory Volume in one second.

Total number = 913	No (%, sd)	FEV_1 ml (95% CI)
**Prior exposures**		
Any wheeze in 1st year of life	423 (46)	-17 (-45 to +12)
Family history of asthma	517 (57)	-18 (-79 to +43)
Attendance at nursery	141 (15)	-49 (-155 to +57)
Paracetamol in 1st year of life	216 (24)	+14 (-25 to +52)
Mean birth weight, N=, Kg (sd) N=911	3.32 (0.53)	+60 (-12 to +131)
**Mean birth height, cm (sd)**	**50 (2)**	**+14 (+6 to +23)**
Breastfeeding ≥ 4months	508 (56)	+14 (-71 to +98)
Age = 1 year		
Mean log IgE, N=460	+3.44 (1.39)	-18 (-50 to +13)
Mean log eosinophils, N=454	-2.14 (1.04)	-14 (-52 to +25)
Age = 2 years		
Helicobacter stool +ve, N=593	29 (5)	-72 (-284 to +140)
Age = 3 years		
Mean log CRP, sd, N=622	-2.13 (2.71)	+1 (-12 to +14)
Log dengue IgG, N= 554	-0.43 (2.01)	+5 (-11 to +21)
Helicobacter stool +ve, N=784	52 (7)	-42 (-110 to +25)
Mean log eosinophils, N=659	-1.78 (1.26)	+21 (-3 to +45)
Toxoplasmosis serology +ve, N=614	366 (60)	+50 (-29 to +128)
Mean log IgE, N=622	+3.68 (1.56)	+6 (-21 to +34)
Medical diagnosis of dengue infection	78 (9)	-24 (-59 to +11)
**Current exposures**		
Male Sex	473 (52)	+47 (-11 to +106)
Age (months), (range)	74 (64 to 83)	+7 (-8 to +23)
Wheeze in past 12 months	356 (39)	-60 (-174 to +55)
**Mean weight, Kg, N=903 (range)**	**23.5 (11 to 51)**	**+13 (+8 to +17)**
**Current height, cm, N=903 (range)**	**119 (90 to 175)**	**+8 (+2 to +14)**
**Mean arm circumference, cm N=903**	**18.4 (2.4)**	**+20 (+14 to +25)**
Number of current smokers in house		
0	452 (50)	0
1	264 (29)	+5 (-11 to +22)
≥ 2	197 (22)	-32 (-109 to +46)
Municipality of residence		
**Arroyo Naranjo**	**375 (41)**	**0**
**Cerro**	**120 (13)**	**+61 (+23 to +99)**
**Habana del Este**	**251 (27)**	**+45 (+38 to +53)**
**La Lisa**	**167 (18)**	**-100 (-127 to -73)**
		**P<0.001**
**Current infection and blood assays**		
Helicobacter stool antigen +ve, N=691	10 (1)	-9 (-231 to +213)
Toxoplasmosis +ve, N=694	441 (64)	+3 (-52 to +58)
Mean log dengue IgG serology, N=693	+1.64 (1.55)	-4 (-21 to +14)
Log CRP, N=692	+1.48 (5.73)	-8 (-32 to +17)
Mean log eosinophils, N=791	-1.60 (1.47)	-7 (-43 to +29)
Mean log IgE, N=694	+4.52 (0.97)	-15 (-98 to +67)
Toxocariasis +ve, N=673	60 (9)	-3 (-128 to +121)
Any atopy* (N=826)	316 (38)	+8 (-59 to +76)

adjusted for sex, age in months and clustering by municipality
*defined as any allergen skin prick test > 3mm larger than saline control
FEV_1 = Forced Expiratory Volume in one second
CRP = C Reactive Protein, sd= standard deviation

For peer review only - http://bmjopen.bmj.com/site/about/guidelines.xhtml

Table 4. Association of exposures with bronchodilatation after inhaled salbutamol

Total number = 903	No (%, sd)	% change in FEV_1 (95% CI)
**Prior exposures**		
Any wheeze in 1st year of life	417 (46)	**+1.94 (+0.81 to +3.08)**
Family history of asthma	510 (56)	**+1.85 (+0.14 to +3.57)**
Attendance at nursery	138 (15)	-3.21 (-9.10 to +2.68)
Paracetamol in 1st year of life	213 (24)	-0.67 (-4.68 to +3.35)
Mean birth weight, Kg (sd) N=901	3.32 (0.53)	**-2.67 (-4.49 to -0.84)**
Mean birth height, cm	50 (2)	+0.06 (-0.56 to +0.69)
Breastfeeding ≥ 4months	503 (56)	+0.61 (-2.87 to +4.09)
Age = 1 year		
Mean log IgE, N=455	+3.42 (1.39)	**+1.68 (+0.54 to +2.82)**
Mean log eosinophils, N=449	-2.13 (1.04)	-0.16 (-2.54 to +2.21)
Age = 2 years		
Helicobacter stool +ve, N=586	28 (5)	-6.41 (-14.94 to +2.10)
Age = 3 years		
Mean log CRP, sd, N=615	-2.12 (2.71)	-0.28 (-0.88 to +0.32)
Log dengue IgG, N= 550	-0.43 (2.01)	-0.38 (-1.50 to +0.74)
Helicobacter stool +ve, N=776	51 (6)	-1.40 (-7.30 to +4.51)
Mean log eosinophils, N=653	-1.78 (1.26)	-0.39 (-4.45 to +3.68)
Toxoplasmosis serology +ve, N=607	362 (60)	-0.45 (-7.06 to +6.16)
Mean log IgE, N=615	+3.68 (1.57)	+0.23 (-1.81 to +2.27)
Medical diagnosis of dengue infection	78 (9)	-0.58 (-8.55 to +7.38)
**Current exposures**		
Male Sex	466 (52)	+2.22 (-0.28 to +4.71)
Age (months), (range)	74 (64 to 83)	-0.13 (-0.47 to +0.20)
Wheeze in past 12 months	353 (39)	+3.61 (-5.80 to +13.02)
Mean current weight, Kg, N=893 (range)	24 (11 to 51)	-0.23 (-0.54 to +0.08)
Current height, cm, N=893	119 (90 to 175)	-0.08 (-0.50 to +0.34)
Mean arm circumference, cm N=893	18.4 (2.4)	-0.14 (-0.81 to +0.54)
Number of current smokers in house		
0	446 (49)	0
1	263 (29)	-0.87 (-5.89 to +4.14)
≥ 2	194 (21)	+2.93 (-3.80 to +9.65)
Municipality of residence		
Arroyo Naranjo	368 (41)	0
Cerro	119 (13)	**-1.34 (-2.56 to -0.11)**
Habana del Este	249 (28)	**-0.07 (-0.46 to +0.31)**
La Lisa	167 (18)	**+6.24 (+5.56 to +6.91)**
		p=0.002
**Current infection and blood assays**		
Helicobacter stool antigen +ve, N=684	10 (1)	+8.89 (-9.17 to +26.96)
Toxoplasmosis +ve, N=687	438 (64)	-0.47 (-3.34 to +2.39)
Mean log dengue IgG serology, N=686	+1.64 (1.56)	+0.30 (-1.44 to +2.03)
Log CRP, N=685	-0.87 (1.40)	+0.37 (-1.64 to +2.38)
Mean log eosinophils, N=782	-161 1.47)	+0.99 (-0.48 to+2.46)
Mean log IgE, N=686	+4.52 (0.98)	+0.64 (-0.18 to +1.45)
Toxocariasis +ve, N=666	58 (9)	-1.03 (-5.29 to +3.23)
Any atopy* (N=816)	314 (38)	-0.47 (-8.61 to +7.68)

adjusted for sex, age in months and clustering by municipality
*defined as any allergen skin prick test > 3mm larger than saline control
FEV_1 = Forced Expiratory Volume in one second, CRP = C Reactive Protein
sd= standard deviation

For peer review only - http://bmjopen.bmj.com/site/about/guidelines.xhtml

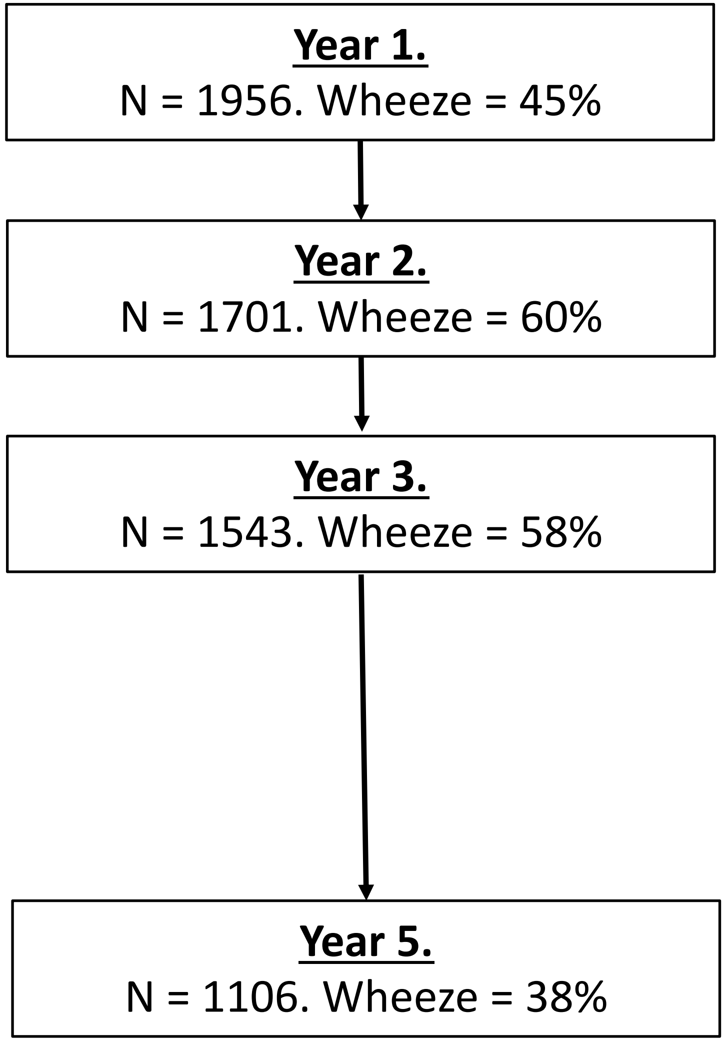
Year 1.
N = 1956. Wheeze = 45%
Year 2.
N = 1701. Wheeze = 60%
Year 3.
N = 1543. Wheeze = 58%
Year 5.
N = 1106. Wheeze = 38%

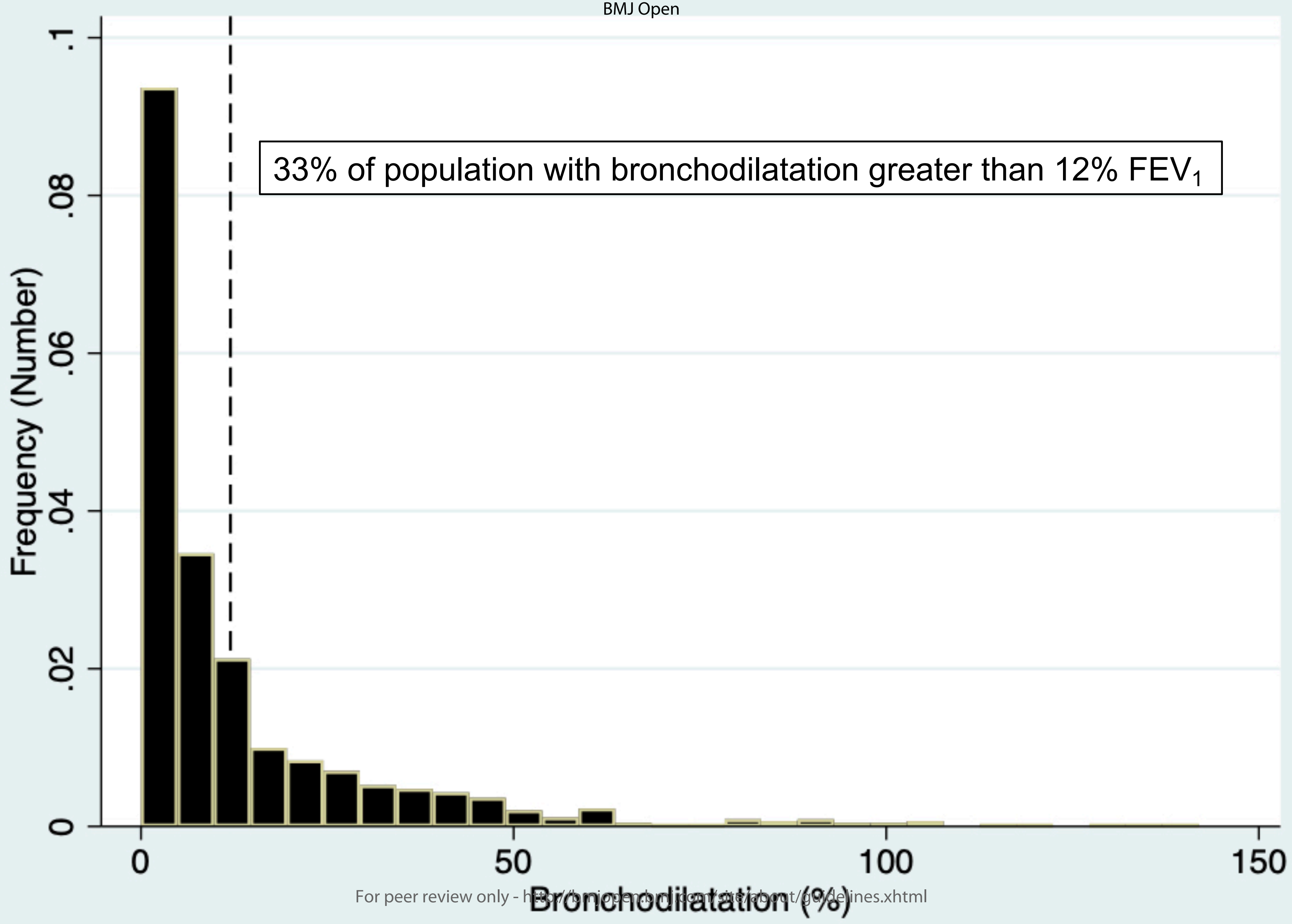
33% of population with bronchodilatation greater than 12% FEV1
Frequency (Number)
0
.02
.04
.06
.08
.1
0
50
100
150
Bronchodilatation (%)

STROBE Statement

	Item No	Recommendation
**P1/2 Title and abstract**	1	(*a*) Indicate the study's design with a commonly used term in the title or the abstract
		(*b*) Provide in the abstract an informative and balanced summary of what was done and what was found
**Introduction**		
P4 Background/rationale	2	Explain the scientific background and rationale for the investigation being reported
P4 Objectives	3	State specific objectives, including any prespecified hypotheses
**Methods**		
P4 Study design	4	Present key elements of study design early in the paper
P4 Setting	5	Describe the setting, locations, and relevant dates, including periods of recruitment, exposure, follow-up, and data collection
P4 Participants	6	(*a*) Give the eligibility criteria, and the sources and methods of selection of participants
P5 Variables	7	Clearly define all outcomes, exposures, predictors, potential confounders, and effect modifiers. Give diagnostic criteria, if applicable
P5 Data sources/ measurement	8*	For each variable of interest, give sources of data and details of methods of assessment (measurement). Describe comparability of assessment methods if there is more than one group
P6 Bias	9	Describe any efforts to address potential sources of bias
P4 Study size	10	Explain how the study size was arrived at
P5 Quantitative variables	11	Explain how quantitative variables were handled in the analyses. If applicable, describe which groupings were chosen and why
P5 Statistical methods	12	(*a*) Describe all statistical methods, including those used to control for confounding
		(*b*) Describe any methods used to examine subgroups and interactions
		(*c*) Explain how missing data were addressed
		(*d*) If applicable, describe analytical methods taking account of sampling strategy
		(*e*) Describe any sensitivity analyses
**Results**		
P16 Participants	13*	(a) Report numbers of individuals at each stage of study—eg numbers potentially eligible, examined for eligibility, confirmed eligible, included in the study, completing follow-up, and analysed
		(b) Give reasons for non-participation at each stage
		(c) Consider use of a flow diagram
P18 Descriptive data	14*	(a) Give characteristics of study participants (eg demographic, clinical, social) and information on exposures and potential confounders
		(b) Indicate number of participants with missing data for each variable of interest
P18-20 Outcome data	15*	Report numbers of outcome events or summary measures
P18-20 Main results	16	(*a*) Give unadjusted estimates and, if applicable, confounder-adjusted estimates and their precision (eg, 95% confidence interval). Make clear which confounders were adjusted for and why they were included
		(*b*) Report category boundaries when continuous variables were categorized
		(*c*) If relevant, consider translating estimates of relative risk into absolute risk for a meaningful time period

For peer review only

P18-20 Other analyses	17	Report other analyses done—eg analyses of subgroups and interactions, and sensitivity analyses
**Discussion**		
P8 Key results	18	Summarise key results with reference to study objectives
P8 Limitations	19	Discuss limitations of the study, taking into account sources of potential bias or imprecision. Discuss both direction and magnitude of any potential bias
P9 Interpretation	20	Give a cautious overall interpretation of results considering objectives, limitations, multiplicity of analyses, results from similar studies, and other relevant evidence
P10 Generalisability	21	Discuss the generalisability (external validity) of the study results
**Other information**		
P12 Funding	22	Give the source of funding and the role of the funders for the present study and, if applicable, for the original study on which the present article is based

*Give information separately for exposed and unexposed groups.

Note: An Explanation and Elaboration article discusses each checklist item and gives methodological background and published examples of transparent reporting. The STROBE checklist is best used in conjunction with this article (freely available on the Web sites of PLoS Medicine at http://www.plosmedicine.org/, Annals of Internal Medicine at http://www.annals.org/, and Epidemiology at http://www.epidem.com/). Information on the STROBE Initiative is available at www.strobe-statement.org.

For peer review only

BMJ Open

Prevalence and risk factors for wheeze, decreased forced expiratory volume in one second and bronchoconstriction in young children living in Havana, Cuba: A population-based cohort study

Journal:	*BMJ Open*
Manuscript ID	bmjopen-2019-034192.R2
Article Type:	Original research
Date Submitted by the Author:	31-Jan-2020
Complete List of Authors:	Suarez-Medina, Ramon; INHEM<br>Venero-Fernández, Silvia; INHEM<br>Alvarez-Valdés, Vilma; Dirección Municipal de Salud Pública municipios Cerro y Arroyo Naranjo<br>Sardiñas-Baez, Nieves; Dirección Municipal de Salud Pública municipios Cerro y Arroyo Naranjo<br>Cristina, Carmona; Dirección Municipal de Salud Pública municipios Cerro y Arroyo Naranjo<br>Loinaz-Gonzalez, Maria; Dirección Municipal de Salud Pública municipios Cerro y Arroyo Naranjo<br>Verdecia-Pérez, Zunilda; Dirección Municipal de Salud Pública municipios Cerro y Arroyo Naranjo<br>Corona-Tamayo, Barbara; Dirección Municipal de Salud Pública municipios Cerro y Arroyo Naranjo<br>Betancourt-López, Maria; INHEM<br>Britton, John; UK Center for Tobacco and Alcohol Studies, Division of Epidemiology and Public Health<br>Fogarty, Andrew; University of Nottingham,
**Primary Subject Heading**:	Respiratory medicine
Secondary Subject Heading:	Global health, Paediatrics
Keywords:	Asthma < THORACIC MEDICINE, Cuba, PAEDIATRICS

SCHOLARONE™
Manuscripts

I, the Submitting Author has the right to grant and does grant on behalf of all authors of the Work (as defined in the below author licence), an exclusive licence and/or a non-exclusive licence for contributions from authors who are: i) UK Crown employees; ii) where BMJ has agreed a CC-BY licence shall apply, and/or iii) in accordance with the terms applicable for US Federal Government officers or employees acting as part of their official duties; on a worldwide, perpetual, irrevocable, royalty-free basis to BMJ Publishing Group Ltd ("BMJ") its licensees and where the relevant Journal is co-owned by BMJ to the co-owners of the Journal, to publish the Work in this journal and any other BMJ products and to exploit all rights, as set out in our licence.

The Submitting Author accepts and understands that any supply made under these terms is made by BMJ to the Submitting Author unless you are acting as an employee on behalf of your employer or a postgraduate student of an affiliated institution which is paying any applicable article publishing charge ("APC") for Open Access articles. Where the Submitting Author wishes to make the Work available on an Open Access basis (and intends to pay the relevant APC), the terms of reuse of such Open Access shall be governed by a Creative Commons licence – details of these licences and which Creative Commons licence will apply to this Work are set out in our licence referred to above.

Other than as permitted in any relevant BMJ Author's Self Archiving Policies, I confirm this Work has not been accepted for publication elsewhere, is not being considered for publication elsewhere and does not duplicate material already published. I confirm all authors consent to publication of this Work and authorise the granting of this licence.

For peer review only

Prevalence and risk factors for wheeze, decreased forced expiratory volume in one second and bronchoconstriction in young children living in Havana, Cuba: A population-based cohort study

* Ramon Suarez-Medina[1]
Silvia Josefina Venero-Fernández[1]
Vilma Alvarez-Valdés[2]
Nieves B Sardiñas-Baez[2]
Cristina Carmona[2]
María Luisa Loinaz-Gonzalez[2]
Zunilda Verdecia-Pérez[2]
Barbara Corona-Tamayo[2]
María Antonia Betancourt-López[1]
John Britton[3],
Andrew W Fogarty[3]
and the HINASIC (Historia Natural de la Sibilancia en Cuba/Natural History of Wheezing in Cuba) Study Group[++]

[1] Instituto Nacional de Higiene, Epidemiología y Microbiología, Infanta No 1158 e/ Llinás y Clavel, Código Postal 10300, La Habana, Cuba.
[2] Dirección Municipal de Salud Pública municipios Cerro y Arroyo Naranjo, La Habana, Cuba.
[3] Nottingham NIHR Biomedical Research Unit, Division of Epidemiology and Public Health, University of Nottingham, Clinical Sciences Building, City Hospital, Nottingham NG5 1PB, UK
[++]full list of collaborating researchers at the end of the manuscript

The corresponding author for the manuscript submission process is Dr Andrew Fogarty (Andrew.fogarty@nottingham.ac.uk) due to poor internet connections with Cuba.

(If accepted for publication) *Correspondence and requests for reprints to Dr. Ramón Suárez Medina:
Address: Infanta No 1158 e/ Llinás y Clavel, Código Postal 10300, La Habana, Cuba.
Office phone: (537) 878 8479
Email: ramonsm@inhem.sld.cu

Key words: Cuba, asthma, children, wheeze

Word count: 3138 words

For peer review only

Abstract (298 words)

Objectives; Asthma has not been extensively studied in low and middle-income countries, where risk factors and access to treatment may differ from more affluent countries. we aimed to identify the prevalence of asthma and local risk factors in Havana, Cuba.

Setting: Four municipalities in Havana, Cuba.

Participants: A population-based cohort study design of young children living in Havana, Cuba. Children were recruited from primary care centres at age 12-15 months.

Primary and secondary outcome measures: Data on wheeze in the past 12 months, asthma treatment and environmental exposures collected regularly until the age of six years, when Forced Expiratory Volume in one second (FEV_1) and reversibility to aerosolised salbutamol were also measured.

Results: 1106 children provided data at the age of six years old. The prevalence of wheeze in the previous 12 months was 422 (38%), and 294 (33%) of the study population had bronchodilatation of 12% or more in FEV_1 after administration of inhaled salbutamol. In the previous 12 months, 182 (16%) of the children had received inhaled corticosteroids, 416 (38%) salbutamol inhalers, and 283 (26%) a course of systemic steroids.

Wheeze in the first year and a family history of asthma were both positively associated with bronchodilatation to inhaled salbutamol (+1.94%; 95% confidence intervals CI: +0.81 to +3.08, and +1.85%; CI: +0.14 to +3.57 respectively), while paracetamol use in the first year was associated with wheeze at six years (OR 1.64, 95% CI: 1.14 to 2.35). There were large differences in FEV_1, bronchodilatation and risk of wheeze across different geographical areas.

Conclusions: Asthma is common in young children living in Havana, and the high prevalence of systemic steroids administrated is likely to reflect the underuse of regular inhaled corticosteroids. If replicated in other comparable low and middle-income countries, this represents an important global public health issue.

Keywords: infection, asthma, children, Cuba, lung function

For peer review only

STRENGTHS AND LIMITATIONS OF THIS STUDY

- There have been many epidemiological studies of asthma in children who live in developed countries, but few population-based studies that have used objective measures of asthma in developing countries.
- The prevalence of bronchoconstriction in young children living in Havana is high using both subjective and objective measures of asthma.
- These data are from one developing country and thus not generalisable to other nations with different economies and health-care systems.
- The high prevalence of administration of systemic steroids in this population-based sample is public health concern, as it is likely to be avoidable, and may increase the risk of side-effects in later life.

For peer review only

Introduction

Asthma is a global disease that affects approximately 11% of six year-old children [1], but with marked regional differences in prevalence [1]. The aetiology of asthma is complex, and involves a range of environmental exposures that are likely to have differential impacts at different ages over the human lifetime [2-7]. The early years of childhood is a particularly important period as this is when the lungs and immune system are developing rapidly, and lung function in children is a key determinant of health in adulthood [8].

This study was established as a consequence of concerns among public health specialists and clinicians that asthma was becoming a large problem in Cuba. It aimed to determine the prevalence of wheeze in young children living in Cuba, and to identify modifiable risk factors for wheezing, reduced lung function and reversible bronchoconstriction. The role of infection in early life in the development of allergic disease remains unclear [9]. The main hypothesis of interest was that infection with parasites [10], *Helicobacter pylori* [11], dengue [12], or systemic inflammation [13] may be associated with wheeze or bronchoconstriction. Exposure to environmental tobacco smoke and paracetamol had previously been observed to be positively associated with wheeze [14] or atopic dermatitis [15] symptoms respectively, and so the association of these exposures with wheeze and bronchoconstriction were also studied. Finally, as growth from *in utero* onwards may also be related to development of asthma and growth of the lungs [16], anthropometric measures from birth onwards were also considered. The study design is a prospective population-based study of an existing cohort of children followed from approximately one year of age for five years.

Methods

Study population and sample collection

The study population is a cohort of 1956 children aged 12 to 15 months who were randomly selected from the general population from four municipalities across Havana in 2010 and 2011 [14 15 17]. The response rate of those who were eligible to participate initially was 96% [14]. Data was collected by a standardised questionnaire that was administered by a member of the study team at baseline and subsequently at 2 years, 3 years and 5 years later. This included a number of health and lifestyle questions that were answered by the parent or guardian and particular attention was paid to parental/guardian reported wheeze in the past 12 months using the methodology developed for the ISAAC epidemiological studies of asthma [18], use of asthma medication in the past 12 months and exposure to environmental tobacco smoke. The child's weight, height and mid-arm circumference in both arms were collected at each study visit. Historical baseline data including birth weight and height were collected from the primary care centre records. At each

For peer review only - http://bmjopen.bmj.com/site/about/guidelines.xhtml

annual follow-up study the participants' guardians were asked if the child had received a medical diagnosis of dengue infection in the past year (in Cuba all positive diagnoses of dengue infection are clinically confirmed using a serological IgM assay) and a blood sample was collected from children to measure circulating eosinophil levels. This sample was stored at -20 degrees Centigrade and subsequently defrosted and analysed for dengue immunoglobulin G (IgG) serology to generate an antibody index [19], serum immunoglobulin E (IgE) [17], highly sensitive C-Reactive Protein (hsCRP, SpinReact, Spain [20]), toxoplasmosis immunoglobulin G antibodies (IgG) [21] and toxocariasis IgG antibodies (DRG Instruments, Germany). A faecal sample was also collected at each review and stored at minus 20°C, and later examined for *Helicobacter pylori* using the faecal antigen test (SpinReact, Spain) and intestinal parasites using the Kato-Katz test (Campiñas Medical COMI, Brazil).

Lung function

Forced expiratory volume in one second (FEV_1) and Forced Vital Capacity were measured in accordance with ATS/ERS criteria [22] using spirometers (CareFusion Micro I) calibrated each day to allow for local climatic change. The best value of FEV_1 within a threshold of repeatability of 200ml was used as the final value. Aerosolised salbutamol (300 μg) was then administered via a spacer and after 15 minutes lung function was measured again to quantify airway reversibility. In children who provided a post-bronchodilator FEV_1 that was less than the baseline value, they were considered as having no reversibility to bronchodilator as this was likely to be due to fatigue.

Allergen skin prick testing

Skin prick testing was used to determine allergy to mite, cat, grass, cockroach, fungus, mosquitos, wheat and soy (allergens from Diater, Argentina except mite allergen from Biocen, Cuba). For each test a drop of allergen solution was placed on the skin and a lancet used to break the skin. After 15 minutes, the skin wheal was measured at its maximum diameter, and also perpendicularly, and a mean value generated. The final skin prick test result was calculated by subtracting the saline result from the allergen. A value of ≥3mm was used to define a positive atopic result for each allergen, and atopy was defined as any positive skin prick test.

Statistical analysis

The main outcome variables were FEV_1, percent increase in FEV_1 after to inhaled salbutamol and wheeze in the past 12 months. The main exposure variables were grouped into three categories:

1. Prior exposures: wheeze in the first year of life, family history of asthma, nursery attendance, birth weight, birth height, duration of breastfeeding, blood IgE and eosinophils at one year old; faecal *Helicobacter pylori* antigen at two and three years old; blood hsCRP,

For peer review only - http://bmjopen.bmj.com/site/about/guidelines.xhtml

dengue IgG serology, eosinophils, toxoplasmosis serology, IgE at three years old, and any prior medical diagnosis of dengue infection.

2. Cross-section exposures: number of smokers living in the home, current weight, current height, mean arm circumference, municipality of residence.
3. Biomarkers of current infection and inflammation: Helicobacter pylori stool antigen, Toxoplasmosis IgG serology, dengue IgG serology, blood hsCRP, eosinophils, IgE, toxocariasis serology and atopy. Less than 2% of children has current gastro-intestinal parasite infection and these data were not analysed further.

Statistical analysis used linear and logistic regression adjusting for sex and age in months as *a priori* confounding factors, and also adjusted for clustering by municipality of residence. As height was associated with FEV_1, all analyses of this outcome measure also adjusted for height to ensure that the analyses were not confounded by somatic growth. Chi-squared tests were used to explore differences in categorical exposures for binary outcome measures. All analyses used Stata 14 statistical software (Texas, USA).

Patient and Public Involvement

The study was designed as a consequence of concerns from the Cuban public health and clinical communities about asthma morbidity. The patients were not involved in the design of the study and patients did not receive a copy of the results. We thank the patients and their families for their participation.

Ethics approval

The study was approved by Ethics Committees in the Instituto Nacional de Higiene, Epidemiología y Microbiología, Cuba and at the University of Nottingham, UK. This involved parental consent on the behalf of the child.

Results

Data were available for 1106 children, of whom 422 (38%) had reported wheeze in the past year (Figure 1). Wheeze in the first year of life was reported in 514 (46%) current participants, while there was a prevalence of wheeze in the first year of life of 42% (358 children) for those who did not participate in the study at the age of six years (p=0.055). Nine hundred and thirteen (83%) children provided lung function data, and of these 903 (99%) children had their reversibility to salbutamol measured. The mean FEV_1 was 1.13L (standard deviation 0.31), and 294 (33%) had

For peer review only - http://bmjopen.bmj.com/site/about/guidelines.xhtml

an increase in FEV_1 of more than 12% after administration of aerosolised salbutamol (Figure 2). Two hundred and eighty-three (26%) of the current study population were reported to have received systemic steroids in the previous 12 months (Table 1).

Risk factors for wheeze in the past 12 months at six years old

The analysis of the association of exposures with wheeze in the past 12 months is presented in Table 2. Both wheeze (odds ratio OR 1.89; 95%CI: 1.65 to 2.16) and paracetamol use (OR 1.64, 95% CI: 1.14 to 2.35) in the first year of life along with a family history of asthma (OR 1.66; 95%CI: 1.40 to 1.97) were associated with wheeze at six years. A positive *Helicobacter pylori* faecal antigen test at age 2 years was negatively associated with wheeze in the past 12 months (OR 0.57; 95%CI: 0.40 to 0.82), but this association was not observed for *Helicobacter pylori* at the age of three years or six years. The number of smokers in the household was a strong risk factor for wheeze in the past 12 months ($p<0.001$ for trend), with homes with two or more smokers having an odds ratio of the child having wheeze in the past 12 months of 2.08 (95% CI: 1.71 to 2.54) compared to those homes with no smokers. The municipality of residence was associated with wheeze in the past 12 months ($p=0.04$, chi^2 test), with children living in Cerro municipality having the highest risk of wheeze (OR 1.72 compared to Arroyo Naranjo; 95%CI: +1.61 to 1.84). These differences were not substantially modified by adjusting for the number of smokers in the home.

Risk factors for decreased FEV_1 at six years old

The analysis of the association of a number of exposures with FEV_1 is presented in Table 3. A number of measures of somatic growth were positively associated with FEV_1 at six years. These were birth height (+14ml per cm height at birth; 95% confidence intervals CI: +6 to +23), current height (+11ml per cm; 95%CI: +5 to +18), current weight (+11ml per Kg; 95%CI: +3 to +18), and current mean arm circumference (+12ml per cm; 95%CI: +1 to +24). The number of smokers living in the home was not associated with lung function, but the municipality of residence was strongly associated with current FEV_1 ($p<0.001$, ANOVA test), with children living in La Lisa having the lowest lung function (-95ml compared to Arroyo Naranjo, 95%CI: -126 to -64). These differences were not substantially modified by adjusting for the number of smokers in the home. No measures of infection or inflammation were associated with lung function.

Risk factors for bronchodilatation after inhaled salbutamol at six years old

The association of exposures with change in FEV_1 from baseline after aerosolised salbutamol was administered is presented in Table 4. Any wheeze in the first year of life was positively associated with bronchodilatation (+1.94%; 95%CI: +0.81 to +3.08) as was a family history of asthma (+1.85;

For peer review only - http://bmjopen.bmj.com/site/about/guidelines.xhtml

95%CI: +0.14 to +3.57), but there was no relation with wheeze in the past 12 months (+3.61; 95%CI: -5.80 to 13.02). Children with a higher birth weight had a lower risk of bronchodilatation (–2.67%, 95% CI: -4.49 to -0.84). IgE at the age of one year was positively associated with a higher risk of bronchodilatation (+1.68%; 95%CI: +0.54 to +2.82), but not IgE at age of three years or six years.

The numbers of smokers living in the child's home was not associated with bronchodilation, but the municipality of residence was again a strong determinant of current bronchodilatation (p=0.002, ANOVA test), with children living in La Lisa having the highest increase in FEV_1 after administration of inhaled salbutamol at six years old (+6.24% compared to Arroyo Naranjo; 95%CI: +5.56 to +6.91). These differences were not substantially modified by adjusting for the number of smokers in the home.

Discussion

The most striking observation from our cohort study of young children living in Havana is that the prevalence of wheeze in these children was high, with 38% reporting wheeze in the past 12 months. Similarly, there was a high prevalence of bronchoconstriction using the objective measure of lung function reversibility to inhaled salbutamol, with 33% of the study population having an increase in FEV_1 of 12% or more. Over a quarter of these six-year old children have received systemic steroids in the previous 12 months, suggesting that asthma control was suboptimal. The main consistent association is that municipality of residence is a strong risk factor for wheeze, low lung function and bronchoconstriction. A history of wheeze in early life, exposure to paracetamol in the first year of life and current environmental tobacco smoke were risk factors for wheeze, anthropometric measures were positively associated with higher FEV_1, and wheeze in the first year of life and lower birth weight were associated with untreated bronchconstriction.

Strengths and limitations of the data

This is the first cohort study of young children living in Cuba that has collected extensive life course data to evaluate risk factors for asthma using objective measures of lung function and reversibility along with laboratory measures of exposure to infection. The strengths of our data include the prospective nature of the data collected with exposures and outcome measures that were selected to permit exploration of risk factors for asthma specifically within an urban Cuban environment, where the lifestyle, environment and microbial exposures are very different to more temperate, developed countries. The measurement of lung function is a challenge in young children, yet measurements were obtained in 82% of eligible children. The availability of lung

For peer review only - http://bmjopen.bmj.com/site/about/guidelines.xhtml
For peer review only

function measurements along with reversibility to inhaled salbutamol is a particular strength of these data, as it provides an objective measure that may reflect different aspects of lung health compared to self-reported symptoms.

Our dataset does have some limitations. We initially recruited 1956 children to the cohort at the age of one year with a response rate of 96% eligible children, and approximately five years later were able to collect data on 1106 (57%) of these. This was mainly a consequence of the recent changes in Cuba that have made it easier for the population more mobile, and hence many children left the policlinic from where they were initially recruited when their family moved their residence. There was only a small difference in the prevalence of wheeze in the first year of life between those who provided data at the age of 6 years (46%) and those who did not (42%), so loss to follow-up is unlikely to constitute a major source of bias. Collecting data on parental or self-reported wheeze using questionnaires is challenging, and although we used the optimal methodology from the ISAAC international studies of allergic disease [18] it is possible that some of the cases of wheeze were not asthma. Similarly, parental reported asthma medication use may be prone to recall bias, although as acute asthma in children is an upsetting family event it is likely to be remembered accurately although the time period possibly less so. This included a number of health and lifestyle questions that were answered by the parent or guardian and particular attention was paid to parental/guardian reported wheeze in the past 12 months using the methodology developed for the ISAAC epidemiological studies of asthma [18], use of asthma medication in the past 12 months and exposure to environmental tobacco smoke. There are no validated lung function normal values in Cuban children aged 6 years old and as a consequence we were unable to generate reliable percent predicted values of the lung function in our study population. Finally, we used the 200ml as the definition of repeatability of FEV_1, which is a value generally used in adults. As a consequence, our population provided FEV_1 measures that will have a higher measurement error than observed in clinical patients. Nonetheless, these data of the peak FEV_1 value records have an epidemiological value as the methodology of data collection was standardised across the whole population. This is supported by the observation of the association between FEV_1 and height, which are both different aspects of somatic growth.

Like many cohorts, our study began as a cross-sectional population-based that aimed to explore causes of the high prevalence of asthma in Habana, Cuba. Further funding permitted this to become a prospective study, with a particular emphasis on infections found in the tropics. As a consequence we have tested a variety of analyses and present the results in their entirety. While we recognise that it is possible to generate a number of papers analysing different hypotheses from these data, this approach has the advantage of presenting a complete body of work that

For peer review only - http://bmjopen.bmj.com/site/about/guidelines.xhtml

hopefully can inform researchers who are interested in this area. While we have considered that sex and age in months are *a priori* confounding factors and adjusted for them in all analyses, we have refrained for searching through all the data to look for other possible confounding factors and possible interactions. The one exception to this is to explore the hypothesis that cigarette smoking may explain the differences observed between municipalities for wheeze, FEV_1 and bronchodilatation as this is an obvious question that was driven by Cuban public health concerns about the hazards of exposure to second-hand tobacco smoke.

Risk factors for wheeze in the previous 12 months at six years old

Wheeze in the first year of life is associated with wheeze in later childhood [23], and may reflect the establishment of the asthmatic phenotype in susceptible children. The observation that paracetamol consumption in early life is associated with wheeze in later life [24] has been noted before, and our data support these observations. However, they do not demonstrate a causality, as an alternative explanation is reverse causality with the administration of more paracetamol to children who are more susceptible to infections and wheeze [25].

The observation that *Helicobacter pylori* at the age of two years, but not three years old or six years old is associated with lower risk of wheeze at six years old is an interesting observation, that would be consistent with the hypothesis that age of exposure to infection is important. Data from other countries has demonstrated the *Helicobacter* is associated with lower rates of allergic disease but not wheeze in children [11 26], but not in adults [27]. It is striking however that our prevalence of Helicobacter pylori infection was very low at less than 7% in the first six years of life, suggesting that this may be less important in Cuba compared to other countries [11].

From a public health perspective, exposure to cigarette smoking and the municipality of residence are the most important exposures for wheeze at the age of six years. Second-hand cigarette smoke exposure is well recognised as a risk factor for wheeze in children [5], and it is a concern that 51% of the homes in our study population contained at least one smoker. The risk of wheeze was higher in the municipalities of Cerro and La Lisa, which suggest that there may be an environmental factor that predisposes to asthma symptoms such as higher levels of air pollution [28-30].

Risk factors for modified FEV_1 at six years old

The fact that children living in La Lisa municipality already have lower lung function at the age of six years is a major public health concern, and suggests that an exposure associated with living in this area may negatively impact on lung growth. La Lisa had by far the highest increase in FEV_1

For peer review only - http://bmjopen.bmj.com/site/about/guidelines.xhtml

after administration of aerosolised salbutamol, suggesting that bronchoconstriction is contributing to the low baseline FEV_1 and that a component of the apparent low lung function is reversible. La Lisa is located inland and while there is no obvious sources of excessive industrial pollution [30], and we speculate that this may be associated with higher levels of air pollution as a consequence of the relative absence of sea winds compared to the other municipalities. Weight, height and mean arm circumference were all positively associated with FEV_1 at six years of age which is likely to be simply because they are all measures of somatic growth.

Risk factors for bronchodilatation after inhaled salbutamol at six years old

Wheeze in the first year of life was associated with bronchodilatation after the administration of inhaled salbutamol at six years. This suggests that symptoms of asthma in early life are predictive of untreated bronchoconstriction five years later, and is consistent with the hypothesis that the asthmatic phenotype can be observed to persist from early life onwards [23]. The observation that birth weight is inversely associated with bronchoconstriction provides objective evidence to support the earlier epidemiological evidence that adults with higher birth weights have a lower risk of having a diagnosis of asthma [16]. The observation that a family history asthma is associated with bronchoconstriction also wheeze is consistent with the knowledge that there is a genetic component to asthma [31].

Self-reported wheeze and bronchoconstriction

One interesting observation from our data is that the wheeze in the past 12 months is not associated with either FEV_1, or the change in FEV_1 after administration of inhaled bronchodilator. Although it is possible that children who have had wheeze subsequently received asthma treatment, this lack of association between asthma symptoms and objective measures of asthma has been described previously [32]. This supports that concept that wheeze and reversibility of FEV_1 after administration of aerosolised salbutamol may measure different aspects of lung development and health.

Public health implications of these data

The current study was designed in response to clinical concerns that asthma was becoming a public health problem in Havana. These data support that hypothesis, and in particular identify that living in certain municipalities may result in higher levels of currently unknown exposures that require public health intervention. It is possible that environmental air pollution may drive some of these geographical differences, and that studies of air pollution are urgently required in Havana. The prevalence of exposure to second-hand tobacco smoke in children living in Havana remains high, and this is an area where a combination of public health interventions including taxation,

For peer review only - http://bmjopen.bmj.com/site/about/guidelines.xhtml

legislation that enforces bans of smoking in the workplace and public places, restrictions on tobacco product advertising, and helping individual smokers quit smoking are known to be effective. We are exploring these hypotheses further by doing further objective measurements of particulate air pollution within the study population.

Summary

Asthma is common in young children living in Havana, and the high prevalence of the use of systemic steroids probably reflects the underuse of regular inhaled corticosteroid prophylaxis treatments leading to the requirement for rescue treatment for wheezing. As societies urbanise, environmental air pollution may increase from a variety of sources. Cuba's economy has struggled as a consequence of a historical and political events [33], and it remains under an economic embargo from the USA that has negatively impacted on healthcare [34]. This makes providing regular inhaled corticosteroids to all children who need them challenging. However, other low and middle-income countries also have difficult economic and environmental circumstances, and if these observations are replicated elsewhere, then this represents an important global public health issue.

For peer review only

For peer review only - http://bmjopen.bmj.com/site/about/guidelines.xhtml

Acknowledgements

We would like to thank the children and their parents and guardians for participating in this study. We would like to thank all of the staff at the municipalities and policlinics who helped in many ways with data collection and sample analysis.

Contributors: The study was conceived and designed by RSM, SVF, AWF and JB. Data were collected by RSM, SVF, VAV, NBSB, CC, MLLG, ZVP, BCT and MABL. Data analysis was conducted by RSM and AWF. RSM, SVF and AWF wrote the first draft of the manuscript. All authors critically reviewed the manuscript, provided intellectual content and approved the final version prior to submission for publication.

Funding: This study was funded by The Wellcome Trust, The University of Nottingham, Nottingham University Hospitals Charity, NIHR Nottingham Biomedical Research Centre and a BMA Award (James Trust).

Competing interests: None declared.

Data sharing statement: Cuban regulations do not allow dissemination of national datasets. However, statistical analyses can be performed upon reasonable requests to the corresponding author.

For peer review only

References

1. Pearce N, Ait-Khaled N, Beasley R, et al. Worldwide trends in the prevalence of asthma symptoms: phase III of the international study of asthma and allergies in childhood (ISAAC). *Thorax* 2007;62:758-66.
2. Jartti T, Gern J. Role of viral infections in the development and exacerbation of asthma in children. *J Allergy Clin Immunology* 2017;140:895-906.
3. Edwards M, Strong K, Cameron A, et al. Viral infections in allergy and immunology: How allergic inflammation influences viral infections and illness. *J Allergy Clin Immunology* 2017;140:909-20.
4. Prescott S. Effects of early cigarette smoke exposure on early immune development and respiratory disease. *Paediatric respiratory reviews* 2008;9:3-10.
5. Lewis S, Antoniak M, Venn A, et al. Secondhand smoke, dietary fruit intake, road traffic exposures, and the prevalence of asthma: A cross-sectional study in young children. *Am J Epidem* 2005;161:406-11.
6. Litonjua A, Gold D. Early-life exposures and later lung function. *Am J Resp Crit Care Med* 2016;193:110-11.
7. Schultz E, Hallberg J, Bellander T, et al. Early-life exposure to traffic-related air pollution and lung function in adolescence. *Am J Resp Crit Care Med* 2016;193:171-77.
8. Sly P, Bush A. From the cradle to the grave: The early-life origins of chronic obstructive pulmonary disease. *Am J Resp Crit Care Med* 2016;193
9. Postma D, Weiss S, den Berge M, et al. Revisiting the dutch hypothesis. *J Allergy Clin Immunology* 2015;136:521-29.
10. Feary J, Britton J, Leonardi-Bee J. Atopy and current intestinal parasite infection: a systematic review and meta-analysis. *Allergy* 2010;66:569-77.
11. Amberbir A, Medhin G, Abegaz W, et al. Exposure to Helicobacter pylori infection in early childhood and the risk of allergic disease and atopic sensitisation: a longitudinal birth cohort study. *Clin Exp Allergy* 2014;44:563-71.
12. Mabalirajan U, Kadhiravan T, Sharma S, et al. TH2 immune response in patients with dengue during defervescence: preliminary evidence. *Am J Trop Med Hygiene* 2005;72:783-85.
13. Fogarty A, Jones S, Britton J, et al. Systemic inflammation and decline in lung function in a general population: a prospective study. *Thorax* 2007;62:515-20.
14. Venero-Fernandez S, Suarez Medina R, Mora Faife E, et al. Risk factors for wheezing in infants born in Cuba. *Quarterly Journal Medicine* 2013;106:1023-29.
15. Suarez-Medina R, Venero-Fernandez S, Mora Faife E, et al. Risk factors for eczema in infants born in Cuba. *BMC Dermatology* 2014;14:6.
16. Shaheen S, Sterne J, Montgomery S, et al. Birth weight, body mass index and asthma in young adults. *Thorax* 1999;54:396-402.
17. Fundora Hernández H, Suarez-Medina R, Venero Fernandez S, et al. What are the main environmental exposures associated with elevated IgE in Cuban infants? A population-based study. *Tropical Medicine and International Health* 2014;19:545-54.
18. Committee TISoAaAiCIS. Worldwide variations in the prevalence of asthma symptoms: the International Study of Asthma and Allergies in Childhood (ISAAC) Steering Committee. *Eur Resp J* 1998;12:315-35.
19. Vircell dengue ELISA IgG. *http://wwwperamedcom/peramed/docs/G1018_ENpdf (accessed 14/4/2016)*
20. Venero-Fernandez S, Fundora-Hernández H, Batista-Gutierrez L, et al. The association of low birth weight with serum C reactive protein in three year old children living in Cuba: A population-based prospective study. *Am J Human Biol* 2016:DOI 10.1002/ajhb.22936.
21. Garcia F, Venero Fernandez S, Fundora Hernández H, et al. Seroprevalencia de anticuerpos IgG anti-Toxoplasma Gondii en infantes de La Habana. *Parasitaria* 2015;73:(in press).
22. Miller M, Hankinson J, Brusasco V, et al. Standardisation of spirometry. *Eur Resp J* 2005;26:319-38.
23. Martinez F, Wright A, Taussig L, et al. Asthma and wheezing in the first six years of life. *New Eng J Med* 1995;332:133-38.

For peer review only - http://bmjopen.bmj.com/site/about/guidelines.xhtml

24. Beasley R, Clayton T, Crane J, et al. Association between paracetamol use in infancy and childhood, and risk of asthma, rhinoconjunctivitis, and eczema in children aged 6-7 years: analysis from Phase Three of the ISAAC programme. *Lancet* 2008;372:1039-48.
25. Rusconi F; Gagliardi L; Galassi C; Forastiere F; Brunetti L; La Grutta S; Piffer S; Talassi F; SIDRIA-2 Collaborative Group. Paracetamol and antibiotics in childhood and subsequent development of wheezing/asthma: association or causation? *Int J Epidemiol* 2011;40:662-67.
26. Amberbir A, Medhin G, Erku W, et al. Effects of Helicobacter pylori, geohelminth infection and selected commensal bacteria on the risk of allergic disease and sensitization in 3 year old Ethiopian children. *Clinical Exp Allergy* 2011;41:1422-30.
27. Fullerton D, Britton J, Lewis S, et al. Helicobacter pylori and lung function, asthma, atopy and allergic disease - A population-based cross-sectional study. *Int J Epidemiol* 2009;38:419-26.
28. Wu W, Jin Y, Carlsten C. Inflammatory health effects of indoor and outdoor particular matter. *J Allergy Clin Immunology* 2018;141:833-44.
29. Gordon S, Bruce N, Grigg J, et al. Respiratory risks from household air pollution in low and middle income countries. *Lancet Respiratory Medicine* 2014;2:823-60.
30. Cuellar-Luna L, Puerto-Rodriguez A, Maldonado-Cantillo G, et al. Distribucion espacial de fuents fijas contaminantes y su impact en salud, provincia La Habana (Cuba). *Hig Sanid Ambient* 2013;13:968-74.
31. Shrine N, Portelli MA, John C, et al. Moderate-to-severe asthma in individuals of European ancestry: a genome-wide association study. *The Lancet Respiratory medicine* 2019;7(1):20-34. doi: 10.1016/S2213-2600(18)30389-8 [published Online First: 2018/12/16]
32. MacKenney J, Oyarzun M, Diaz P, et al. Prevalence of asthma, atopy and bronchial hyperresponsiveness and their interrelation in a semi-rural area of Chile. *Int J Tuberculosis & Lung Dis* 2005;9:1288-93.
33. Gott R. Cuba. New Haven, USA: Yale University Press 2010.
34. Barry M. Effect of the US embargo and economic decline on health in Cuba. *Ann Intern Med* 2000;132:151-54.

For peer review only

Figure 1. Flow diagram of participants and data collection

Figure 1 goes here

For peer review only

Figure 2. Histogram of percent increase in Forced Expiratory Volume in one second in study population (N=903 children)

Figure 2 goes here

For peer review only - http://bmjopen.bmj.com/site/about/guidelines.xhtml

Table 1. Description of study population

	Total (N=1106)	Provided FEV_1 (N=913)
Male sex (%)	575 (52)	473 (52)
Mean age, months (range)	74 (63 to 83)	74 (64 to 83)
Mean FEV_1, L, (sd)	-	1.13 (0.31)
Mean bronchodilation after salbutamol, % (sd)	-	13.4 (20.1) N=903
Wheeze in the past 12 months (%)	422 (38)	356 (39)
**Received inhaled steroids in the 12 months before (%):**		
Year 1	89 (8)	68 (7)
Year 2	176 (17) N=1051	153 (18) N=869
Year 3	203 (18)	163 (18)
Year 4	-	-
Year 5	182 (16)	150 (16)
**Received salbutamol inhaler in the previous 12 months (%):**		
Year 5	416 (38)	345 (38)
**Received IV or oral steroids in the 12 months before (%):**		
Year 1	295 (27)	244 (27)
Year 2	418 (40) N=1051	345 (40) N=869
Year 3	407 (37)	333 (36)
Year 4	-	-
Year 5	283 (26)	240 (26)

For peer review only

Table 2. Association of exposures with wheeze in past 12 months.

**Total number = 1106**	No (%, sd)	Odds ratio of wheeze (95% CI)
**Prior exposures**		
**Any wheeze in 1st year of life**	514 (46)	**1.89 (1.65 to 2.16)**
**Family history of asthma**	614 (56)	**1.66 (1.40 to 1.97)**
Attendance at nursery	159 (14)	0.84 (0.57 to 1.24)
**Paracetamol in 1st year of life**	256 (23)	**1.64 (1.14 to 2.35)**
Mean birth weight, N=, Kg (sd) N=1104	3.31 (0.51)	0.87 (0.75 to 1.01)
Mean birth height, cm (sd)	50.2 (2.4)	0.95 (0.89 to 1.00)
Breastfeeding ≥ 4months	608 (55)	0.83 (0.66 to 1.03)
Age = 1 year		
Mean log IgE, N=885	+3.38 (1.47)	1.06 (0.88 to 1.27)
Mean log eosinophils, N=856	-2.11(1.05)	0.93 (0.77 to 1.13)
Age = 2 years		
**Helicobacter stool +ve, N=1067**	40 (4)	0.57 (0.40 to 0.82)
Age = 3 years		
Mean log CRP, sd, N=986	-2.07 (2.70)	1.01 (0.95 to 1.07)
Log dengue IgG, N= 865	-0.44 (2.00)	0.99 (0.96 to 1.02)
Helicobacter stool +ve, N=951	58 (6)	1.01 (0.64 to 1.60)
**Mean log eosinophils, N=1039**	-1.77 (1.23)	0.91 (0.86 to 0.96)
Toxoplasmosis serology +ve, N=966	565 (58)	1.15 (0.82 to 1.64)
Mean log IgE, N=986	3.70 (1.53)	1.14 (0.98 to 1.31)
Medical diagnosis of dengue infection	93 (8)	1.40 (0.91 to 2.14)
**Current exposures**		
Male Sex	575 (52)	**1.35 (1.00 to 1.82)**
Age (months), (range)	74 (63 to 83)	**0.97 (0.96 to 0.99)**
Mean current weight, Kg, N=1062	23.3 (11 to 51)	1.01 (0.97 to 1.06)
Current height, cm, N=1057	119 (7)	1.00 (0.97 to 1.03)
Mean arm circumference, cm N=1062	18.3 (2.4)	1.02 (0.95 to 1.08)
Number of current smokers in house		
0	546 (49)	0
1	322 (29)	1.61 (1.22 to 2.12)
≥ 2	238 (22)	2.08 (1.71 to 2.54)
Municipality of residence		p_{TREND}<0.001
**Arroyo Naranjo**	455 (41)	**0**
**Cerro**	139 (13)	**1.72 (1.61 to 1.84)**
**Habana del Este**	307 (28)	**0.86 (0.84 to 0.88)**
**La Lisa**	205 (19)	**1.24 (1.21 to 1.27)**
		**p=0.04**
**Current infection and blood assays**		
Helicobacter stool antigen +ve, N=756	11 (1)	1.45 (0.63 to 3.33)
Toxoplasmosis +ve, N=759	487 (64)	0.97 (0.70 to 1.34)
Mean log dengue IgG serology, N=758	1.67 (1.53)	0.95 (0.90 to 1.01)
Log CRP, N=757	-0.83 (1.41)	0.99 (0.86 to 1.15)
Mean log eosinophils, N=868	-1.62 (1.45)	1.02 (0.90 to 1.15)
**Mean log IgE, N=759**	4.51 (0.97)	1.27 (1.15 to 1.41)
Toxocariasis +ve, N=673	64 (9)	0.96 (0.70 to 1.33)
**Any atopy*** (N=857)	52 (6)	0.81 (0.43 to 1.53)

For peer review only

For peer review only - http://bmjopen.bmj.com/site/about/guidelines.xhtml

Table 3. Association of exposures with Forced Expiratory Volume in one second.

**Total number = 903**	No (%, sd)	FEV_1 ml (95% CI)
**Prior exposures**		
Any wheeze in 1st year of life	416 (46)	-16 (-50 to +18)
Family history of asthma	510 (56)	-23 (-89 to +43)
Attendance at nursery	141 (16)	-48 (-149 to +53)
Paracetamol in 1st year of life	215 (24)	+8 (-36 to +51)
Mean birth weight, N=, Kg (sd) N=901	3.32 (0.53)	+43 (-19 to +104)
**Mean birth height, cm (sd)**	**50 (2)**	**+11 (+5 to +18)**
Breastfeeding ≥ 4months	503 (56)	+18 (-65 to +101)
Age = 1 year		
Mean log IgE, N=456	+3.44 (1.39)	-19 (-50 to +12)
Mean log eosinophils, N=451	-2.14 (1.04)	-13 (-56 to +30)
Age = 2 years		
Helicobacter stool +ve, N=589	29 (5)	-78 (-270 to +115)
Age = 3 years		
Mean log CRP, sd, N=614	-2.16 (2.71)	+2 (-9 to +12)
Log dengue IgG, N= 545	-0.43 (2.00)	+7 (-10 to +25)
Helicobacter stool +ve, N=775	51 (7)	-39 (-132 to +54)
Mean log eosinophils, N=652	-1.78 (1.26)	+22 (-5 to +49)
Toxoplasmosis serology +ve, N=606	362 (60)	+48 (-46 to +143)
Mean log IgE, N=614	+3.68 (1.57)	+8 (-21 to +37)
Medical diagnosis of dengue infection	78 (9)	-27 (-59 to +5)
**Current exposures**		
Male Sex	468 (52)	+36 (-23 to +96)
Mean age (months), (range)	74 (64 to 83)	+4 (-12 to +21)
Wheeze in past 12 months	353 (39)	-62 (-160 to +35)
**Mean weight, Kg, N=902 (range)**	**23.5 (11 to 51)**	**+11 (+3 to +18)**
**Current height, cm, N=903 (range)**	**119 (90 to 175)**	**+8 (+2 to +14)**
**Mean arm circumference, cm N=901**	**18.4 (2.4)**	**+12 (+1 to +24)**
Number of current smokers in house		
0	447 (49)	0
1	261 (29)	0 (-20 to +19)
≥ 2	195 (22)	-39 (-108 to +30)
Municipality of residence		
**Arroyo Naranjo**	**371 (41)**	**0**
**Cerro**	**119 (13)**	**+74 (+31 to 117)**
**Habana del Este**	**247 (27)**	**+48 (+39 to +57)**
**La Lisa**	**166 (18)**	**-95 (-126 to -64) p<0.001**
**Current infection and blood assays**		
Helicobacter stool antigen +ve, N=685	10 (1)	-10 (-228 to +207)
Toxoplasmosis +ve, N=688	437 (64)	+8 (-49 to +65)
Mean log dengue IgG serology, N=687	+1.63 (1.56)	-5 (-24 to +14)
Log CRP, N=686	-0.87 (1.38)	-9 (-37 to +20)
Mean log eosinophils, N=785	-1.61 (1.47)	-5 (-41 to +31)
Mean log IgE, N=688	+4.52 (0.97)	-12 (-87 to +63)
Toxocariasis +ve, N=667	57 (9)	-15 (-125 to +95)
Any atopy* (N=818)	52 (6)	-54 (-182 to +74)

adjusted for sex, age in months, current height and clustering by municipality
*defined as any allergen skin prick test > 3mm larger than saline control
FEV_1 = Forced Expiratory Volume in one second
CRP = C Reactive Protein, sd= standard deviation

For peer review only - http://bmjopen.bmj.com/site/about/guidelines.xhtml

Table 4. Association of exposures with bronchodilatation after inhaled salbutamol

**Total number = 903**	No (%, sd)	% change in FEV_1 (95% CI)
**Prior exposures**		
Any wheeze in 1st year of life	417 (46)	**+1.94 (+0.81 to +3.08)**
Family history of asthma	510 (56)	**+1.85 (+0.14 to +3.57)**
Attendance at nursery	138 (15)	-3.21 (-9.10 to +2.68)
Paracetamol in 1st year of life	213 (24)	-0.67 (-4.68 to +3.35)
Mean birth weight, Kg (sd) N=901	3.32 (0.53)	**-2.67 (-4.49 to -0.84)**
Mean birth height, cm	50 (2)	+0.06 (-0.56 to +0.69)
Breastfeeding ≥ 4months	503 (56)	+0.61 (-2.87 to +4.09)
Age = 1 year		
Mean log IgE, N=455	+3.42 (1.39)	**+1.68 (+0.54 to +2.82)**
Mean log eosinophils, N=449	-2.13 (1.04)	-0.16 (-2.54 to +2.21)
Age = 2 years		
Helicobacter stool +ve, N=586	28 (5)	-6.41 (-14.94 to +2.10)
Age = 3 years		
Mean log CRP, sd, N=615	-2.12 (2.71)	-0.28 (-0.88 to +0.32)
Log dengue IgG, N= 550	-0.43 (2.01)	-0.38 (-1.50 to +0.74)
Helicobacter stool +ve, N=776	51 (6)	-1.40 (-7.30 to +4.51)
Mean log eosinophils, N=653	-1.78 (1.26)	-0.39 (-4.45 to +3.68)
Toxoplasmosis serology +ve, N=607	362 (60)	-0.45 (-7.06 to +6.16)
Mean log IgE, N=615	+3.68 (1.57)	+0.23 (-1.81 to +2.27)
Medical diagnosis of dengue infection	78 (9)	-0.58 (-8.55 to +7.38)
**Current exposures**		
Male Sex	466 (52)	+2.22 (-0.28 to +4.71)
Age (months), (range)	74 (64 to 83)	-0.13 (-0.47 to +0.20)
Wheeze in past 12 months	353 (39)	+3.61 (-5.80 to +13.02)
Mean current weight, Kg, N=893 (range)	24 (11 to 51)	-0.23 (-0.54 to +0.08)
Current height, cm, N=893	119 (90 to 175)	-0.08 (-0.50 to +0.34)
Mean arm circumference, cm N=893	18.4 (2.4)	-0.14 (-0.81 to +0.54)
Number of current smokers in house		
0	446 (49)	0
1	263 (29)	-0.87 (-5.89 to +4.14)
≥ 2	194 (21)	+2.93 (-3.80 to +9.65)
Municipality of residence		
Arroyo Naranjo	368 (41)	0
Cerro	119 (13)	**-1.34 (-2.56 to -0.11)**
Habana del Este	249 (28)	**-0.07 (-0.46 to +0.31)**
La Lisa	167 (18)	**+6.24 (+5.56 to +6.91)**
		p=0.002
**Current infection and blood assays**		
Helicobacter stool antigen +ve, N=684	10 (1)	+8.89 (-9.17 to +26.96)
Toxoplasmosis +ve, N=687	438 (64)	-0.47 (-3.34 to +2.39)
Mean log dengue IgG serology, N=686	+1.64 (1.56)	+0.30 (-1.44 to +2.03)
Log CRP, N=685	-0.87 (1.40)	+0.37 (-1.64 to +2.38)
Mean log eosinophils, N=782	-161 1.47)	+0.99 (-0.48 to+2.46)
Mean log IgE, N=686	+4.52 (0.98)	+0.64 (-0.18 to +1.45)
Toxocariasis +ve, N=666	58 (9)	-1.03 (-5.29 to +3.23)
Any atopy* (N=816)	52 (6)	-2.75 (-10.15 to +4.60)

adjusted for sex, age in months and clustering by municipality
*defined as any allergen skin prick test > 3mm larger than saline control
FEV_1 = Forced Expiratory Volume in one second, CRP = C Reactive Protein
sd= standard deviation

For peer review only - http://bmjopen.bmj.com/site/about/guidelines.xhtml

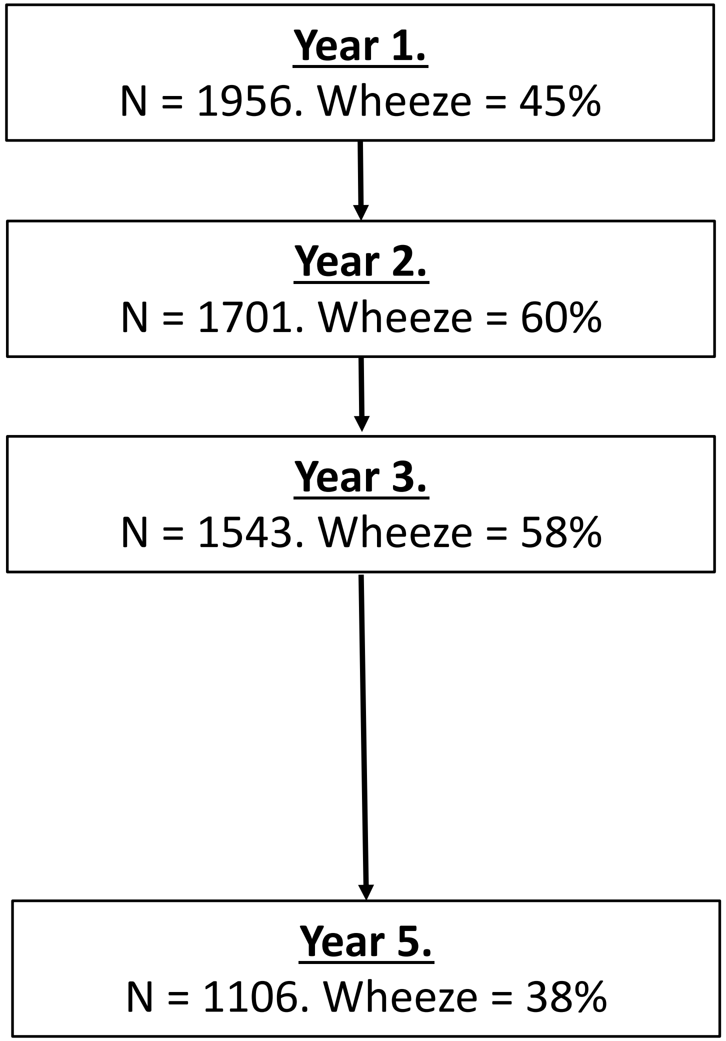
Year 1.
N = 1956. Wheeze = 45%
Year 2.
N = 1701. Wheeze = 60%
Year 3.
N = 1543. Wheeze = 58%
Year 5.
N = 1106. Wheeze = 38%

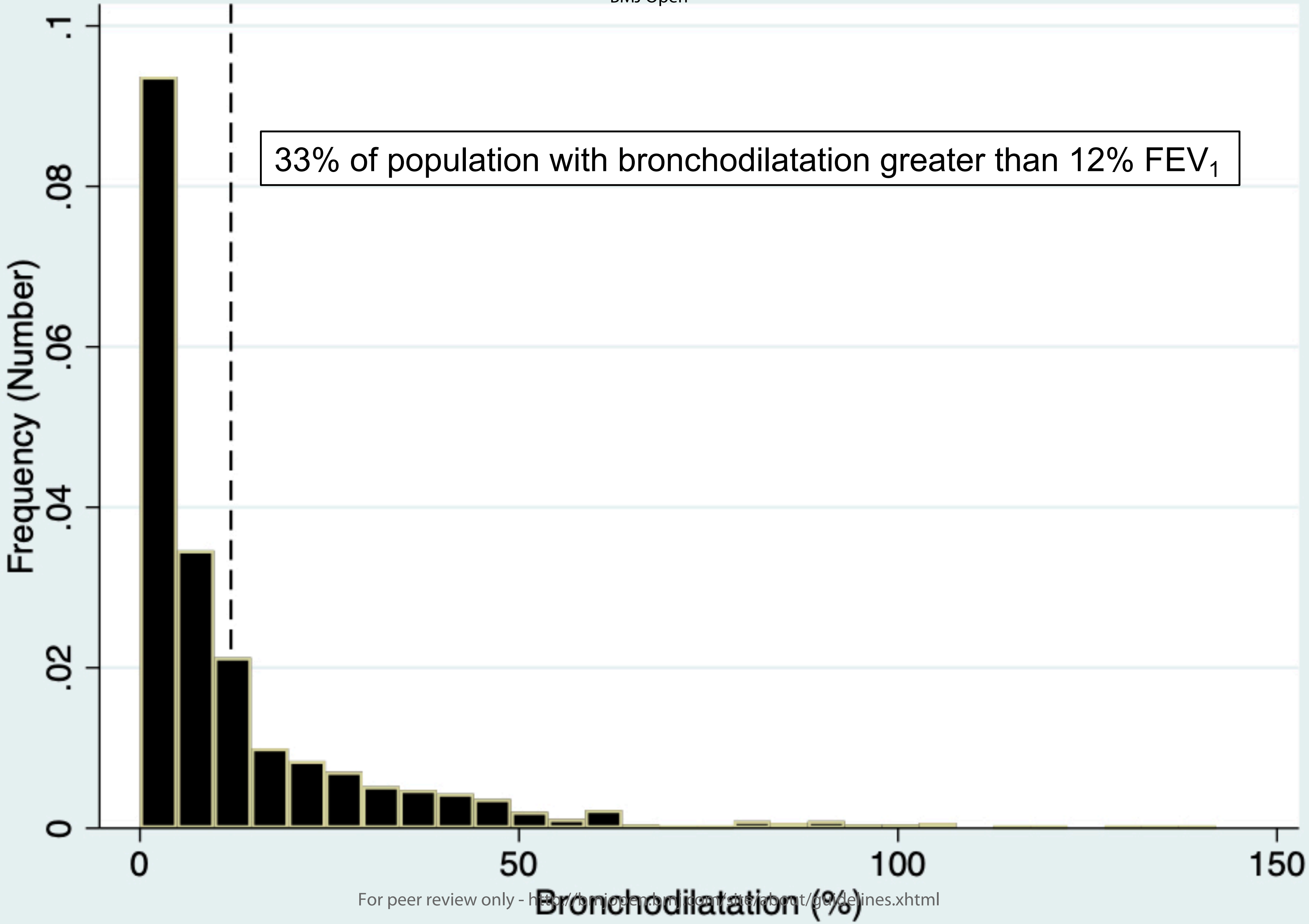
33% of population with bronchodilatation greater than 12% FEV$_1$
Frequency (Number)
0
.02
.04
.06
.08
.1
0
50
100
150
Bronchodilatation (%)

STROBE Statement

	Item No	Recommendation
**P1/2 Title and abstract**	1	(*a*) Indicate the study's design with a commonly used term in the title or the abstract
		(*b*) Provide in the abstract an informative and balanced summary of what was done and what was found
**Introduction**		
P4 Background/rationale	2	Explain the scientific background and rationale for the investigation being reported
P4 Objectives	3	State specific objectives, including any prespecified hypotheses
**Methods**		
P4 Study design	4	Present key elements of study design early in the paper
P4 Setting	5	Describe the setting, locations, and relevant dates, including periods of recruitment, exposure, follow-up, and data collection
P4 Participants	6	(*a*) Give the eligibility criteria, and the sources and methods of selection of participants
P5 Variables	7	Clearly define all outcomes, exposures, predictors, potential confounders, and effect modifiers. Give diagnostic criteria, if applicable
P5 Data sources/ measurement	8*	For each variable of interest, give sources of data and details of methods of assessment (measurement). Describe comparability of assessment methods if there is more than one group
P6 Bias	9	Describe any efforts to address potential sources of bias
P4 Study size	10	Explain how the study size was arrived at
P5 Quantitative variables	11	Explain how quantitative variables were handled in the analyses. If applicable, describe which groupings were chosen and why
P5 Statistical methods	12	(*a*) Describe all statistical methods, including those used to control for confounding
		(*b*) Describe any methods used to examine subgroups and interactions
		(*c*) Explain how missing data were addressed
		(*d*) If applicable, describe analytical methods taking account of sampling strategy
		(*e*) Describe any sensitivity analyses
**Results**		
P16 Participants	13*	(a) Report numbers of individuals at each stage of study—eg numbers potentially eligible, examined for eligibility, confirmed eligible, included in the study, completing follow-up, and analysed
		(b) Give reasons for non-participation at each stage
		(c) Consider use of a flow diagram
P18 Descriptive data	14*	(a) Give characteristics of study participants (eg demographic, clinical, social) and information on exposures and potential confounders
		(b) Indicate number of participants with missing data for each variable of interest
P18-20 Outcome data	15*	Report numbers of outcome events or summary measures
P18-20 Main results	16	(*a*) Give unadjusted estimates and, if applicable, confounder-adjusted estimates and their precision (eg, 95% confidence interval). Make clear which confounders were adjusted for and why they were included
		(*b*) Report category boundaries when continuous variables were categorized
		(*c*) If relevant, consider translating estimates of relative risk into absolute risk for a meaningful time period

For peer review only - http://bmjopen.bmj.com/site/about/guidelines.xhtml

P18-20 Other analyses	17	Report other analyses done—eg analyses of subgroups and interactions, and sensitivity analyses
**Discussion**		
P8 Key results	18	Summarise key results with reference to study objectives
P8 Limitations	19	Discuss limitations of the study, taking into account sources of potential bias or imprecision. Discuss both direction and magnitude of any potential bias
P9 Interpretation	20	Give a cautious overall interpretation of results considering objectives, limitations, multiplicity of analyses, results from similar studies, and other relevant evidence
P10 Generalisability	21	Discuss the generalisability (external validity) of the study results
**Other information**		
P12 Funding	22	Give the source of funding and the role of the funders for the present study and, if applicable, for the original study on which the present article is based

*Give information separately for exposed and unexposed groups.

Note: An Explanation and Elaboration article discusses each checklist item and gives methodological background and published examples of transparent reporting. The STROBE checklist is best used in conjunction with this article (freely available on the Web sites of PLoS Medicine at http://www.plosmedicine.org/, Annals of Internal Medicine at http://www.annals.org/, and Epidemiology at http://www.epidem.com/). Information on the STROBE Initiative is available at www.strobe-statement.org.

For peer review only

Prevalence and risk factors for wheeze, decreased forced expiratory volume in one second and bronchoconstriction in young children living in Havana, Cuba: A population-based cohort study

Journal:	*BMJ Open*
Manuscript ID	bmjopen-2019-034192.R3
Article Type:	Original research
Date Submitted by the Author:	12-Feb-2020
Complete List of Authors:	Suarez-Medina, Ramon; INHEM<br>Venero-Fernández, Silvia; INHEM<br>Alvarez-Valdés, Vilma; Dirección Municipal de Salud Pública municipios Cerro y Arroyo Naranjo<br>Sardiñas-Baez, Nieves; Dirección Municipal de Salud Pública municipios Cerro y Arroyo Naranjo<br>Cristina, Carmona; Dirección Municipal de Salud Pública municipios Cerro y Arroyo Naranjo<br>Loinaz-Gonzalez, Maria; Dirección Municipal de Salud Pública municipios Cerro y Arroyo Naranjo<br>Verdecia-Pérez, Zunilda; Dirección Municipal de Salud Pública municipios Cerro y Arroyo Naranjo<br>Corona-Tamayo, Barbara; Dirección Municipal de Salud Pública municipios Cerro y Arroyo Naranjo<br>Betancourt-López, Maria; INHEM<br>Britton, John; UK Center for Tobacco and Alcohol Studies, Division of Epidemiology and Public Health<br>Fogarty, Andrew; University of Nottingham,
**Primary Subject Heading**:	Respiratory medicine
Secondary Subject Heading:	Global health, Paediatrics
Keywords:	Asthma < THORACIC MEDICINE, Cuba, PAEDIATRICS

SCHOLARONE™ Manuscripts

BMJ

I, the Submitting Author has the right to grant and does grant on behalf of all authors of the Work (as defined in the below author licence), an exclusive licence and/or a non-exclusive licence for contributions from authors who are: i) UK Crown employees; ii) where BMJ has agreed a CC-BY licence shall apply, and/or iii) in accordance with the terms applicable for US Federal Government officers or employees acting as part of their official duties; on a worldwide, perpetual, irrevocable, royalty-free basis to BMJ Publishing Group Ltd ("BMJ") its licensees and where the relevant Journal is co-owned by BMJ to the co-owners of the Journal, to publish the Work in this journal and any other BMJ products and to exploit all rights, as set out in our licence.

The Submitting Author accepts and understands that any supply made under these terms is made by BMJ to the Submitting Author unless you are acting as an employee on behalf of your employer or a postgraduate student of an affiliated institution which is paying any applicable article publishing charge ("APC") for Open Access articles. Where the Submitting Author wishes to make the Work available on an Open Access basis (and intends to pay the relevant APC), the terms of reuse of such Open Access shall be governed by a Creative Commons licence – details of these licences and which Creative Commons licence will apply to this Work are set out in our licence referred to above.

Other than as permitted in any relevant BMJ Author's Self Archiving Policies, I confirm this Work has not been accepted for publication elsewhere, is not being considered for publication elsewhere and does not duplicate material already published. I confirm all authors consent to publication of this Work and authorise the granting of this licence.

For peer review only

Prevalence and risk factors for wheeze, decreased forced expiratory volume in one second and bronchoconstriction in young children living in Havana, Cuba: A population-based cohort study

* Ramon Suarez-Medina[1]
Silvia Josefina Venero-Fernández[1]
Vilma Alvarez-Valdés[2]
Nieves B Sardiñas-Baez[2]
Cristina Carmona[2]
María Luisa Loinaz-Gonzalez[2]
Zunilda Verdecia-Pérez[2]
Barbara Corona-Tamayo[2]
María Antonia Betancourt-López[1]
John Britton[3],
Andrew W Fogarty[3]
and the HINASIC (Historia Natural de la Sibilancia en Cuba/Natural History of Wheezing in Cuba) Study Group[++]

[1] Instituto Nacional de Higiene, Epidemiología y Microbiología, Infanta No 1158 e/ Llinás y Clavel, Código Postal 10300, La Habana, Cuba.
[2] Dirección Municipal de Salud Pública municipios Cerro y Arroyo Naranjo, La Habana, Cuba.
[3] Nottingham NIHR Biomedical Research Unit, Division of Epidemiology and Public Health, University of Nottingham, Clinical Sciences Building, City Hospital, Nottingham NG5 1PB, UK
[++]full list of collaborating researchers at the end of the manuscript

The corresponding author for the manuscript submission process is Dr Andrew Fogarty (Andrew.fogarty@nottingham.ac.uk) due to poor internet connections with Cuba.

(If accepted for publication) *Correspondence and requests for reprints to Dr. Ramón Suárez Medina:
Address: Infanta No 1158 e/ Llinás y Clavel, Código Postal 10300, La Habana, Cuba.
Office phone: (537) 878 8479
Email: ramonsm@inhem.sld.cu

Key words: Cuba, asthma, children, wheeze

Word count: 3138 words

For peer review only

Abstract (298 words)

Objectives; Asthma has not been extensively studied in low and middle-income countries, where risk factors and access to treatment may differ from more affluent countries. We aimed to identify the prevalence of asthma and local risk factors in Havana, Cuba.

Setting: Four municipalities in Havana, Cuba.

Participants: A population-based cohort study design of young children living in Havana, Cuba. Children were recruited from primary care centres at age 12-15 months.

Primary and secondary outcome measures: Data on wheeze in the past 12 months, asthma treatment and environmental exposures collected regularly until the age of six years, when Forced Expiratory Volume in one second (FEV_1) and reversibility to aerosolised salbutamol were also measured.

Results: 1106 children provided data at the age of six years old. The prevalence of wheeze in the previous 12 months was 422 (38%), and 294 (33%) of the study population had bronchodilatation of 12% or more in FEV_1 after administration of inhaled salbutamol. In the previous 12 months, 182 (16%) of the children had received inhaled corticosteroids, 416 (38%) salbutamol inhalers, and 283 (26%) a course of systemic steroids.

Wheeze in the first year and a family history of asthma were both positively associated with bronchodilatation to inhaled salbutamol (+1.94%; 95% confidence intervals CI: +0.81 to +3.08, and +1.85%; CI: +0.14 to +3.57 respectively), while paracetamol use in the first year was associated with wheeze at six years (OR 1.64, 95% CI: 1.14 to 2.35). There were large differences in FEV_1, bronchodilatation and risk of wheeze across different geographical areas.

Conclusions: Asthma is common in young children living in Havana, and the high prevalence of systemic steroids administrated is likely to reflect the underuse of regular inhaled corticosteroids. If replicated in other comparable low and middle-income countries, this represents an important global public health issue.

Keywords: infection, asthma, children, Cuba, lung function

For peer review only

STRENGTHS AND LIMITATIONS OF THIS STUDY

- There have been many epidemiological studies of asthma in children who live in developed countries, but few population-based studies that have used objective measures of asthma in developing countries.
- The prevalence of bronchoconstriction in young children living in Havana was measured using both subjective and objective measures of asthma.
- Life course exposure data were available from birth onwards.
- Data on lung development were objectively measured using spirometry.
- These data are from one developing country and thus not generalisable to other nations with different economies and health-care systems.

For peer review only

Introduction

Asthma is a global disease that affects approximately 11% of six year-old children [1], but with marked regional differences in prevalence [1]. The aetiology of asthma is complex, and involves a range of environmental exposures that are likely to have differential impacts at different ages over the human lifetime [2-7]. The early years of childhood is a particularly important period as this is when the lungs and immune system are developing rapidly, and lung function in children is a key determinant of health in adulthood [8].

This study was established as a consequence of concerns among public health specialists and clinicians that asthma was becoming a large problem in Cuba. It aimed to determine the prevalence of wheeze in young children living in Cuba, and to identify modifiable risk factors for wheezing, reduced lung function and reversible bronchoconstriction. The role of infection in early life in the development of allergic disease remains unclear [9]. The main hypothesis of interest was that infection with parasites [10], *Helicobacter pylori* [11], dengue [12], or systemic inflammation [13] may be associated with wheeze or bronchoconstriction. Exposure to environmental tobacco smoke and paracetamol had previously been observed to be positively associated with wheeze [14] or atopic dermatitis [15] symptoms respectively, and so the association of these exposures with wheeze and bronchoconstriction were also studied. Finally, as growth from *in utero* onwards may also be related to development of asthma and growth of the lungs [16], anthropometric measures from birth onwards were also considered. The study design is a prospective population-based study of an existing cohort of children followed from approximately one year of age for five years.

Methods

Study population and sample collection

The study population is a cohort of 1956 children aged 12 to 15 months who were randomly selected from the general population from four municipalities across Havana in 2010 and 2011 [14 15 17]. The response rate of those who were eligible to participate initially was 96% [14]. Data was collected by a standardised questionnaire that was administered by a member of the study team at baseline and subsequently at 2 years, 3 years and 5 years later. This included a number of health and lifestyle questions that were answered by the parent or guardian and particular attention was paid to parental/guardian reported wheeze in the past 12 months using the methodology developed for the ISAAC epidemiological studies of asthma [18], use of asthma medication in the past 12 months and exposure to environmental tobacco smoke. The child's weight, height and mid-arm circumference in both arms were collected at each study visit. Historical baseline data including birth weight and height were collected from the primary care centre records. At each

For peer review only

annual follow-up study the participants' guardians were asked if the child had received a medical diagnosis of dengue infection in the past year (in Cuba all positive diagnoses of dengue infection are clinically confirmed using a serological IgM assay) and a blood sample was collected from children to measure circulating eosinophil levels. This sample was stored at -20 degrees Centigrade and subsequently defrosted and analysed for dengue immunoglobulin G (IgG) serology to generate an antibody index [19], serum immunoglobulin E (IgE) [17], highly sensitive C-Reactive Protein (hsCRP, SpinReact, Spain [20]), toxoplasmosis immunoglobulin G antibodies (IgG) [21] and toxocariasis IgG antibodies (DRG Instruments, Germany). A faecal sample was also collected at each review and stored at minus 20°C, and later examined for *Helicobacter pylori* using the faecal antigen test (SpinReact, Spain) and intestinal parasites using the Kato-Katz test (Campiñas Medical COMI, Brazil).

Lung function

Forced expiratory volume in one second (FEV_1) and Forced Vital Capacity were measured in accordance with ATS/ERS criteria [22] using spirometers (CareFusion Micro I) calibrated each day to allow for local climatic change. The best value of FEV_1 within a threshold of repeatability of 200ml was used as the final value. Aerosolised salbutamol (300 μg) was then administered via a spacer and after 15 minutes lung function was measured again to quantify airway reversibility. In children who provided a post-bronchodilator FEV_1 that was less than the baseline value, they were considered as having no reversibility to bronchodilator as this was likely to be due to fatigue.

Allergen skin prick testing

Skin prick testing was used to determine allergy to mite, cat, grass, cockroach, fungus, mosquitos, wheat and soy (allergens from Diater, Argentina except mite allergen from Biocen, Cuba). For each test a drop of allergen solution was placed on the skin and a lancet used to break the skin. After 15 minutes, the skin wheal was measured at its maximum diameter, and also perpendicularly, and a mean value generated. The final skin prick test result was calculated by subtracting the saline result from the allergen. A value of ≥3mm was used to define a positive atopic result for each allergen, and atopy was defined as any positive skin prick test.

Statistical analysis

The main outcome variables were FEV_1, percent increase in FEV_1 after to inhaled salbutamol and wheeze in the past 12 months. The main exposure variables were grouped into three categories:

1. Prior exposures: wheeze in the first year of life, family history of asthma, nursery attendance, birth weight, birth height, duration of breastfeeding, blood IgE and eosinophils at one year old; faecal *Helicobacter pylori* antigen at two and three years old; blood hsCRP,

For peer review only - http://bmjopen.bmj.com/site/about/guidelines.xhtml

dengue IgG serology, eosinophils, toxoplasmosis serology, IgE at three years old, and any prior medical diagnosis of dengue infection.

2. Cross-section exposures: number of smokers living in the home, current weight, current height, mean arm circumference, municipality of residence.
3. Biomarkers of current infection and inflammation: Helicobacter pylori stool antigen, Toxoplasmosis IgG serology, dengue IgG serology, blood hsCRP, eosinophils, IgE, toxocariasis serology and atopy. Less than 2% of children has current gastro-intestinal parasite infection and these data were not analysed further.

Statistical analysis used linear and logistic regression adjusting for sex and age in months as *a priori* confounding factors, and also adjusted for clustering by municipality of residence. As height was associated with FEV_1, all analyses of this outcome measure also adjusted for height to ensure that the analyses were not confounded by somatic growth. Chi-squared tests were used to explore differences in categorical exposures for binary outcome measures. All analyses used Stata 14 statistical software (Texas, USA).

Patient and Public Involvement

The study was designed as a consequence of concerns from the Cuban public health and clinical communities about asthma morbidity. The patients were not involved in the design of the study and patients did not receive a copy of the results. We thank the patients and their families for their participation.

Ethics approval

The study was approved by Ethics Committees in the Instituto Nacional de Higiene, Epidemiología y Microbiología, Cuba and at the University of Nottingham, UK. Consent on the behalf of the child was provided by the attending parent or guardian.

Results

Data were available for 1106 children, of whom 422 (38%) had reported wheeze in the past year (Figure 1). Wheeze in the first year of life was reported in 514 (46%) current participants, while there was a prevalence of wheeze in the first year of life of 42% (358 children) for those who did not participate in the study at the age of six years (p=0.055). Nine hundred and thirteen (83%) children provided lung function data, and of these 903 (99%) children had their reversibility to salbutamol measured. The mean FEV_1 was 1.13L (standard deviation 0.31), and 294 (33%) had

For peer review only - http://bmjopen.bmj.com/site/about/guidelines.xhtml

an increase in FEV_1 of more than 12% after administration of aerosolised salbutamol (Figure 2). Two hundred and eighty-three (26%) of the current study population were reported to have received systemic steroids in the previous 12 months (Table 1).

Risk factors for wheeze in the past 12 months at six years old

The analysis of the association of exposures with wheeze in the past 12 months is presented in Table 2. Both wheeze (odds ratio OR 1.89; 95%CI: 1.65 to 2.16) and paracetamol use (OR 1.64, 95% CI: 1.14 to 2.35) in the first year of life along with a family history of asthma (OR 1.66; 95%CI: 1.40 to 1.97) were associated with wheeze at six years. A positive *Helicobacter pylori* faecal antigen test at age 2 years was negatively associated with wheeze in the past 12 months (OR 0.57; 95%CI: 0.40 to 0.82), but this association was not observed for *Helicobacter pylori* at the age of three years or six years. The number of smokers in the household was a strong risk factor for wheeze in the past 12 months ($p<0.001$ for trend), with homes with two or more smokers having an odds ratio of the child having wheeze in the past 12 months of 2.08 (95% CI: 1.71 to 2.54) compared to those homes with no smokers. The municipality of residence was associated with wheeze in the past 12 months ($p=0.04$, chi^2 test), with children living in Cerro municipality having the highest risk of wheeze (OR 1.72 compared to Arroyo Naranjo; 95%CI: +1.61 to 1.84). These differences were not substantially modified by adjusting for the number of smokers in the home.

Risk factors for decreased FEV_1 at six years old

The analysis of the association of a number of exposures with FEV_1 is presented in Table 3. A number of measures of somatic growth were positively associated with FEV_1 at six years. These were birth height (+14ml per cm height at birth; 95% confidence intervals CI: +6 to +23), current height (+11ml per cm; 95%CI: +5 to +18), current weight (+11ml per Kg; 95%CI: +3 to +18), and current mean arm circumference (+12ml per cm; 95%CI: +1 to +24). The number of smokers living in the home was not associated with lung function, but the municipality of residence was strongly associated with current FEV_1 ($p<0.001$, ANOVA test), with children living in La Lisa having the lowest lung function (-95ml compared to Arroyo Naranjo, 95%CI: -126 to -64). These differences were not substantially modified by adjusting for the number of smokers in the home. No measures of infection or inflammation were associated with lung function.

Risk factors for bronchodilatation after inhaled salbutamol at six years old

The association of exposures with change in FEV_1 from baseline after aerosolised salbutamol was administered is presented in Table 4. Any wheeze in the first year of life was positively associated with bronchodilatation (+1.94%; 95%CI: +0.81 to +3.08) as was a family history of asthma (+1.85;

For peer review only - http://bmjopen.bmj.com/site/about/guidelines.xhtml

95%CI: +0.14 to +3.57), but there was no relation with wheeze in the past 12 months (+3.61; 95%CI: -5.80 to 13.02). Children with a higher birth weight had a lower risk of bronchodilatation (–2.67%, 95% CI: -4.49 to -0.84). IgE at the age of one year was positively associated with a higher risk of bronchodilatation (+1.68%; 95%CI: +0.54 to +2.82), but not IgE at age of three years or six years.

The numbers of smokers living in the child's home was not associated with bronchodilation, but the municipality of residence was again a strong determinant of current bronchodilatation (p=0.002, ANOVA test), with children living in La Lisa having the highest increase in FEV_1 after administration of inhaled salbutamol at six years old (+6.24% compared to Arroyo Naranjo; 95%CI: +5.56 to +6.91). These differences were not substantially modified by adjusting for the number of smokers in the home.

Discussion

The most striking observation from our cohort study of young children living in Havana is that the prevalence of wheeze in these children was high, with 38% reporting wheeze in the past 12 months. Similarly, there was a high prevalence of bronchoconstriction using the objective measure of lung function reversibility to inhaled salbutamol, with 33% of the study population having an increase in FEV_1 of 12% or more. Over a quarter of these six-year old children have received systemic steroids in the previous 12 months, suggesting that asthma control was suboptimal. The main consistent association is that municipality of residence is a strong risk factor for wheeze, low lung function and bronchoconstriction. A history of wheeze in early life, exposure to paracetamol in the first year of life and current environmental tobacco smoke were risk factors for wheeze, anthropometric measures were positively associated with higher FEV_1, and wheeze in the first year of life and lower birth weight were associated with untreated bronchconstriction.

Strengths and limitations of the data

This is the first cohort study of young children living in Cuba that has collected extensive life course data to evaluate risk factors for asthma using objective measures of lung function and reversibility along with laboratory measures of exposure to infection. The strengths of our data include the prospective nature of the data collected with exposures and outcome measures that were selected to permit exploration of risk factors for asthma specifically within an urban Cuban environment, where the lifestyle, environment and microbial exposures are very different to more temperate, developed countries. The measurement of lung function is a challenge in young children, yet measurements were obtained in 82% of eligible children. The availability of lung

For peer review only - http://bmjopen.bmj.com/site/about/guidelines.xhtml

function measurements along with reversibility to inhaled salbutamol is a particular strength of these data, as it provides an objective measure that may reflect different aspects of lung health compared to self-reported symptoms.

Our dataset does have some limitations. We initially recruited 1956 children to the cohort at the age of one year with a response rate of 96% eligible children, and approximately five years later were able to collect data on 1106 (57%) of these. This was mainly a consequence of the recent changes in Cuba that have made it easier for the population more mobile, and hence many children left the policlinic from where they were initially recruited when their family moved their residence. There was only a small difference in the prevalence of wheeze in the first year of life between those who provided data at the age of 6 years (46%) and those who did not (42%), so loss to follow-up is unlikely to constitute a major source of bias. Collecting data on parental or self-reported wheeze using questionnaires is challenging, and although we used the optimal methodology from the ISAAC international studies of allergic disease [18] it is possible that some of the cases of wheeze were not asthma. Similarly, parental reported asthma medication use may be prone to recall bias, although as acute asthma in children is an upsetting family event it is likely to be remembered accurately although the time period possibly less so. This included a number of health and lifestyle questions that were answered by the parent or guardian and particular attention was paid to parental/guardian reported wheeze in the past 12 months using the methodology developed for the ISAAC epidemiological studies of asthma [18], use of asthma medication in the past 12 months and exposure to environmental tobacco smoke. There are no validated lung function normal values in Cuban children aged 6 years old and as a consequence we were unable to generate reliable percent predicted values of the lung function in our study population. Finally, we used the 200ml as the definition of repeatability of FEV_1, which is a value generally used in adults. As a consequence, our population provided FEV_1 measures that will have a higher measurement error than observed in clinical patients. Nonetheless, these data of the peak FEV_1 value records have an epidemiological value as the methodology of data collection was standardised across the whole population. This is supported by the observation of the association between FEV_1 and height, which are both different aspects of somatic growth.

Like many cohorts, our study began as a cross-sectional population-based that aimed to explore causes of the high prevalence of asthma in Habana, Cuba. Further funding permitted this to become a prospective study, with a particular emphasis on infections found in the tropics. As a consequence we have tested a variety of analyses and present the results in their entirety. While we recognise that it is possible to generate a number of papers analysing different hypotheses from these data, this approach has the advantage of presenting a complete body of work that

For peer review only - http://bmjopen.bmj.com/site/about/guidelines.xhtml

hopefully can inform researchers who are interested in this area. While we have considered that sex and age in months are *a priori* confounding factors and adjusted for them in all analyses, we have refrained for searching through all the data to look for other possible confounding factors and possible interactions. The one exception to this is to explore the hypothesis that cigarette smoking may explain the differences observed between municipalities for wheeze, FEV_1 and bronchodilatation as this is an obvious question that was driven by Cuban public health concerns about the hazards of exposure to second-hand tobacco smoke.

Risk factors for wheeze in the previous 12 months at six years old

Wheeze in the first year of life is associated with wheeze in later childhood [23], and may reflect the establishment of the asthmatic phenotype in susceptible children. The observation that paracetamol consumption in early life is associated with wheeze in later life [24] has been noted before, and our data support these observations. However, they do not demonstrate a causality, as an alternative explanation is reverse causality with the administration of more paracetamol to children who are more susceptible to infections and wheeze [25].

The observation that *Helicobacter pylori* at the age of two years, but not three years old or six years old is associated with lower risk of wheeze at six years old is an interesting observation, that would be consistent with the hypothesis that age of exposure to infection is important. Data from other countries has demonstrated the *Helicobacter* is associated with lower rates of allergic disease but not wheeze in children [11 26], but not in adults [27]. It is striking however that our prevalence of Helicobacter pylori infection was very low at less than 7% in the first six years of life, suggesting that this may be less important in Cuba compared to other countries [11].

From a public health perspective, exposure to cigarette smoking and the municipality of residence are the most important exposures for wheeze at the age of six years. Second-hand cigarette smoke exposure is well recognised as a risk factor for wheeze in children [5], and it is a concern that 51% of the homes in our study population contained at least one smoker. The risk of wheeze was higher in the municipalities of Cerro and La Lisa, which suggest that there may be an environmental factor that predisposes to asthma symptoms such as higher levels of air pollution [28-30].

Risk factors for modified FEV_1 at six years old

The fact that children living in La Lisa municipality already have lower lung function at the age of six years is a major public health concern, and suggests that an exposure associated with living in this area may negatively impact on lung growth. La Lisa had by far the highest increase in FEV_1

For peer review only - http://bmjopen.bmj.com/site/about/guidelines.xhtml

after administration of aerosolised salbutamol, suggesting that bronchoconstriction is contributing to the low baseline FEV_1 and that a component of the apparent low lung function is reversible. La Lisa is located inland and while there is no obvious sources of excessive industrial pollution [30], and we speculate that this may be associated with higher levels of air pollution as a consequence of the relative absence of sea winds compared to the other municipalities. Weight, height and mean arm circumference were all positively associated with FEV_1 at six years of age which is likely to be simply because they are all measures of somatic growth.

Risk factors for bronchodilatation after inhaled salbutamol at six years old

Wheeze in the first year of life was associated with bronchodilatation after the administration of inhaled salbutamol at six years. This suggests that symptoms of asthma in early life are predictive of untreated bronchoconstriction five years later, and is consistent with the hypothesis that the asthmatic phenotype can be observed to persist from early life onwards [23]. The observation that birth weight is inversely associated with bronchoconstriction provides objective evidence to support the earlier epidemiological evidence that adults with higher birth weights have a lower risk of having a diagnosis of asthma [16]. The observation that a family history asthma is associated with bronchoconstriction also wheeze is consistent with the knowledge that there is a genetic component to asthma [31].

Self-reported wheeze and bronchoconstriction

One interesting observation from our data is that the wheeze in the past 12 months is not associated with either FEV_1, or the change in FEV_1 after administration of inhaled bronchodilator. Although it is possible that children who have had wheeze subsequently received asthma treatment, this lack of association between asthma symptoms and objective measures of asthma has been described previously [32]. This supports that concept that wheeze and reversibility of FEV_1 after administration of aerosolised salbutamol may measure different aspects of lung development and health.

Public health implications of these data

The current study was designed in response to clinical concerns that asthma was becoming a public health problem in Havana. These data support that hypothesis, and in particular identify that living in certain municipalities may result in higher levels of currently unknown exposures that require public health intervention. It is possible that environmental air pollution may drive some of these geographical differences, and that studies of air pollution are urgently required in Havana. The prevalence of exposure to second-hand tobacco smoke in children living in Havana remains high, and this is an area where a combination of public health interventions including taxation,

For peer review only - http://bmjopen.bmj.com/site/about/guidelines.xhtml

legislation that enforces bans of smoking in the workplace and public places, restrictions on tobacco product advertising, and helping individual smokers quit smoking are known to be effective. We are exploring these hypotheses further by doing further objective measurements of particulate air pollution within the study population.

Summary

Asthma is common in young children living in Havana, and the high prevalence of the use of systemic steroids probably reflects the underuse of regular inhaled corticosteroid prophylaxis treatments leading to the requirement for rescue treatment for wheezing. As societies urbanise, environmental air pollution may increase from a variety of sources. Cuba's economy has been inversely affected due to historical and political events [33], and it remains under an economic embargo from the USA that has negatively impacted on healthcare [34]. This makes providing regular inhaled corticosteroids to all children who need them challenging. However, other low and middle-income countries also have difficult economic and environmental circumstances, and if these observations are replicated elsewhere, then this represents an important global public health issue.

For peer review only - http://bmjopen.bmj.com/site/about/guidelines.xhtml

Acknowledgements

We would like to thank the children and their parents and guardians for participating in this study. We would like to thank all of the staff at the municipalities and policlinics who helped in many ways with data collection and sample analysis.

Contributors: The study was conceived and designed by RSM, SVF, AWF and JB. Data were collected by RSM, SVF, VAV, NBSB, CC, MLLG, ZVP, BCT and MABL. Data analysis was conducted by RSM and AWF. RSM, SVF and AWF wrote the first draft of the manuscript. All authors critically reviewed the manuscript, provided intellectual content and approved the final version prior to submission for publication.

Funding: This study was funded by The Wellcome Trust, The University of Nottingham, Nottingham University Hospitals Charity, NIHR Nottingham Biomedical Research Centre and a BMA Award (James Trust).

Competing interests: None declared.

Data sharing statement: Cuban regulations do not allow dissemination of national datasets. However, statistical analyses can be performed upon reasonable requests to the corresponding author.

For peer review only

References

1. Pearce N, Ait-Khaled N, Beasley R, et al. Worldwide trends in the prevalence of asthma symptoms: phase III of the international study of asthma and allergies in childhood (ISAAC). *Thorax* 2007;62:758-66.
2. Jartti T, Gern J. Role of viral infections in the development and exacerbation of asthma in children. *J Allergy Clin Immunology* 2017;140:895-906.
3. Edwards M, Strong K, Cameron A, et al. Viral infections in allergy and immunology: How allergic inflammation influences viral infections and illness. *J Allergy Clin Immunology* 2017;140:909-20.
4. Prescott S. Effects of early cigarette smoke exposure on early immune development and respiratory disease. *Paediatric respiratory reviews* 2008;9:3-10.
5. Lewis S, Antoniak M, Venn A, et al. Secondhand smoke, dietary fruit intake, road traffic exposures, and the prevalence of asthma: A cross-sectional study in young children. *Am J Epidem* 2005;161:406-11.
6. Litonjua A, Gold D. Early-life exposures and later lung function. *Am J Resp Crit Care Med* 2016;193:110-11.
7. Schultz E, Hallberg J, Bellander T, et al. Early-life exposure to traffic-related air pollution and lung function in adolescence. *Am J Resp Crit Care Med* 2016;193:171-77.
8. Sly P, Bush A. From the cradle to the grave: The early-life origins of chronic obstructive pulmonary disease. *Am J Resp Crit Care Med* 2016;193
9. Postma D, Weiss S, den Berge M, et al. Revisiting the dutch hypothesis. *J Allergy Clin Immunology* 2015;136:521-29.
10. Feary J, Britton J, Leonardi-Bee J. Atopy and current intestinal parasite infection: a systematic review and meta-analysis. *Allergy* 2010;66:569-77.
11. Amberbir A, Medhin G, Abegaz W, et al. Exposure to Helicobacter pylori infection in early childhood and the risk of allergic disease and atopic sensitisation: a longitudinal birth cohort study. *Clin Exp Allergy* 2014;44:563-71.
12. Mabalirajan U, Kadhiravan T, Sharma S, et al. TH2 immune response in patients with dengue during defervescence: preliminary evidence. *Am J Trop Med Hygiene* 2005;72:783-85.
13. Fogarty A, Jones S, Britton J, et al. Systemic inflammation and decline in lung function in a general population: a prospective study. *Thorax* 2007;62:515-20.
14. Venero-Fernandez S, Suarez Medina R, Mora Faife E, et al. Risk factors for wheezing in infants born in Cuba. *Quarterly Journal Medicine* 2013;106:1023-29.
15. Suarez-Medina R, Venero-Fernandez S, Mora Faife E, et al. Risk factors for eczema in infants born in Cuba. *BMC Dermatology* 2014;14:6.
16. Shaheen S, Sterne J, Montgomery S, et al. Birth weight, body mass index and asthma in young adults. *Thorax* 1999;54:396-402.
17. Fundora Hernández H, Suarez-Medina R, Venero Fernandez S, et al. What are the main environmental exposures associated with elevated IgE in Cuban infants? A population-based study. *Tropical Medicine and International Health* 2014;19:545-54.
18. Committee TISoAaAiCIS. Worldwide variations in the prevalence of asthma symptoms: the International Study of Asthma and Allergies in Childhood (ISAAC) Steering Committee. *Eur Resp J* 1998;12:315-35.
19. Vircell dengue ELISA IgG. *http://wwwperamedcom/peramed/docs/G1018_ENpdf (accessed 14/4/2016)*
20. Venero-Fernandez S, Fundora-Hernández H, Batista-Gutierrez L, et al. The association of low birth weight with serum C reactive protein in three year old children living in Cuba: A population-based prospective study. *Am J Human Biol* 2016:DOI 10.1002/ajhb.22936.
21. Garcia F, Venero Fernandez S, Fundora Hernández H, et al. Seroprevalencia de anticuerpos IgG anti-Toxoplasma Gondii en infantes de La Habana. *Parasitaria* 2015;73:(in press).
22. Miller M, Hankinson J, Brusasco V, et al. Standardisation of spirometry. *Eur Resp J* 2005;26:319-38.
23. Martinez F, Wright A, Taussig L, et al. Asthma and wheezing in the first six years of life. *New Eng J Med* 1995;332:133-38.

For peer review only - http://bmjopen.bmj.com/site/about/guidelines.xhtml

24. Beasley R, Clayton T, Crane J, et al. Association between paracetamol use in infancy and childhood, and risk of asthma, rhinoconjunctivitis, and eczema in children aged 6-7 years: analysis from Phase Three of the ISAAC programme. *Lancet* 2008;372:1039-48.
25. Rusconi F; Gagliardi L; Galassi C; Forastiere F; Brunetti L; La Grutta S; Piffer S; Talassi F; SIDRIA-2 Collaborative Group. Paracetamol and antibiotics in childhood and subsequent development of wheezing/asthma: association or causation? *Int J Epidemiol* 2011;40:662-67.
26. Amberbir A, Medhin G, Erku W, et al. Effects of Helicobacter pylori, geohelminth infection and selected commensal bacteria on the risk of allergic disease and sensitization in 3 year old Ethiopian children. *Clinical Exp Allergy* 2011;41:1422-30.
27. Fullerton D, Britton J, Lewis S, et al. Helicobacter pylori and lung function, asthma, atopy and allergic disease - A population-based cross-sectional study. *Int J Epidemiol* 2009;38:419-26.
28. Wu W, Jin Y, Carlsten C. Inflammatory health effects of indoor and outdoor particular matter. *J Allergy Clin Immunology* 2018;141:833-44.
29. Gordon S, Bruce N, Grigg J, et al. Respiratory risks from household air pollution in low and middle income countries. *Lancet Respiratory Medicine* 2014;2:823-60.
30. Cuellar-Luna L, Puerto-Rodriguez A, Maldonado-Cantillo G, et al. Distribucion espacial de fuents fijas contaminantes y su impact en salud, provincia La Habana (Cuba). *Hig Sanid Ambient* 2013;13:968-74.
31. Shrine N, Portelli MA, John C, et al. Moderate-to-severe asthma in individuals of European ancestry: a genome-wide association study. *The Lancet Respiratory medicine* 2019;7(1):20-34. doi: 10.1016/S2213-2600(18)30389-8 [published Online First: 2018/12/16]
32. MacKenney J, Oyarzun M, Diaz P, et al. Prevalence of asthma, atopy and bronchial hyperresponsiveness and their interrelation in a semi-rural area of Chile. *Int J Tuberculosis & Lung Dis* 2005;9:1288-93.
33. Gott R. Cuba. New Haven, USA: Yale University Press 2010.
34. Barry M. Effect of the US embargo and economic decline on health in Cuba. *Ann Intern Med* 2000;132:151-54.

For peer review only

For peer review only - http://bmjopen.bmj.com/site/about/guidelines.xhtml

Figure 1. Flow diagram of participants and data collection

Figure 1 goes here

Figure 2. Histogram of percent increase in Forced Expiratory Volume in one second in study population (N=903 children)

Figure 2 goes here

For peer review only

Table 1. Description of study population

	Total (N=1106)	Provided FEV_1 (N=913)
Male sex (%)	575 (52)	473 (52)
Mean age, months (range)	74 (63 to 83)	74 (64 to 83)
Mean FEV_1, L, (sd)	-	1.13 (0.31)
Mean bronchodilation after salbutamol, % (sd)	-	13.4 (20.1) N=903
Wheeze in the past 12 months (%)	422 (38)	356 (39)
**Received inhaled steroids in the 12 months before (%):**		
Year 1	89 (8)	68 (7)
Year 2	176 (17) N=1051	153 (18) N=869
Year 3	203 (18)	163 (18)
Year 4	-	-
Year 5	182 (16)	150 (16)
**Received salbutamol inhaler in the previous 12 months (%):**		
Year 5	416 (38)	345 (38)
**Received IV or oral steroids in the 12 months before (%):**		
Year 1	295 (27)	244 (27)
Year 2	418 (40) N=1051	345 (40) N=869
Year 3	407 (37)	333 (36)
Year 4	-	-
Year 5	283 (26)	240 (26)

For peer review only

Table 2. Association of exposures with wheeze in past 12 months.

**Total number = 1106**	No (%, sd)	Odds ratio of wheeze (95% CI)
**Prior exposures**		
**Any wheeze in 1st year of life**	514 (46)	**1.89 (1.65 to 2.16)**
**Family history of asthma**	614 (56)	**1.66 (1.40 to 1.97)**
Attendance at nursery	159 (14)	0.84 (0.57 to 1.24)
**Paracetamol in 1st year of life**	256 (23)	**1.64 (1.14 to 2.35)**
Mean birth weight, N=, Kg (sd) N=1104	3.31 (0.51)	0.87 (0.75 to 1.01)
Mean birth height, cm (sd)	50.2 (2.4)	0.95 (0.89 to 1.00)
Breastfeeding ≥ 4months	608 (55)	0.83 (0.66 to 1.03)
Age = 1 year		
Mean log IgE, N=885	+3.38 (1.47)	1.06 (0.88 to 1.27)
Mean log eosinophils, N=856	-2.11(1.05)	0.93 (0.77 to 1.13)
Age = 2 years		
**Helicobacter stool +ve, N=1067**	40 (4)	0.57 (0.40 to 0.82)
Age = 3 years		
Mean log CRP, sd, N=986	-2.07 (2.70)	1.01 (0.95 to 1.07)
Log dengue IgG, N= 865	-0.44 (2.00)	0.99 (0.96 to 1.02)
Helicobacter stool +ve, N=951	58 (6)	1.01 (0.64 to 1.60)
**Mean log eosinophils, N=1039**	-1.77 (1.23)	0.91 (0.86 to 0.96)
Toxoplasmosis serology +ve, N=966	565 (58)	1.15 (0.82 to 1.64)
Mean log IgE, N=986	3.70 (1.53)	1.14 (0.98 to 1.31)
Medical diagnosis of dengue infection	93 (8)	1.40 (0.91 to 2.14)
**Current exposures**		
Male Sex	575 (52)	**1.35 (1.00 to 1.82)**
Age (months), (range)	74 (63 to 83)	**0.97 (0.96 to 0.99)**
Mean current weight, Kg, N=1062	23.3 (11 to 51)	1.01 (0.97 to 1.06)
Current height, cm, N=1057	119 (7)	1.00 (0.97 to 1.03)
Mean arm circumference, cm N=1062	18.3 (2.4)	1.02 (0.95 to 1.08)
Number of current smokers in house		
0	546 (49)	0
1	322 (29)	1.61 (1.22 to 2.12)
≥ 2	238 (22)	2.08 (1.71 to 2.54)
Municipality of residence		p_{TREND}<0.001
**Arroyo Naranjo**	455 (41)	**0**
**Cerro**	139 (13)	**1.72 (1.61 to 1.84)**
**Habana del Este**	307 (28)	**0.86 (0.84 to 0.88)**
**La Lisa**	205 (19)	**1.24 (1.21 to 1.27)**
		**p=0.04**
**Current infection and blood assays**		
Helicobacter stool antigen +ve, N=756	11 (1)	1.45 (0.63 to 3.33)
Toxoplasmosis +ve, N=759	487 (64)	0.97 (0.70 to 1.34)
Mean log dengue IgG serology, N=758	1.67 (1.53)	0.95 (0.90 to 1.01)
Log CRP, N=757	-0.83 (1.41)	0.99 (0.86 to 1.15)
Mean log eosinophils, N=868	-1.62 (1.45)	1.02 (0.90 to 1.15)
**Mean log IgE, N=759**	4.51 (0.97)	1.27 (1.15 to 1.41)
Toxocariasis +ve, N=673	64 (9)	0.96 (0.70 to 1.33)
**Any atopy*** (N=857)	52 (6)	0.81 (0.43 to 1.53)

For peer review only - http://bmjopen.bmj.com/site/about/guidelines.xhtml

Table 3. Association of exposures with Forced Expiratory Volume in one second.

Total number = 903	No (%, sd)	FEV_1, ml (95% CI)
**Prior exposures**		
Any wheeze in 1st year of life	416 (46)	-16 (-50 to +18)
Family history of asthma	510 (56)	-23 (-89 to +43)
Attendance at nursery	141 (16)	-48 (-149 to +53)
Paracetamol in 1st year of life	215 (24)	+8 (-36 to +51)
Mean birth weight, N=, Kg (sd) N=901	3.32 (0.53)	+43 (-19 to +104)
**Mean birth height, cm (sd)**	**50 (2)**	**+11 (+5 to +18)**
Breastfeeding ≥ 4months	503 (56)	+18 (-65 to +101)
Age = 1 year		
Mean log IgE, N=456	+3.44 (1.39)	-19 (-50 to +12)
Mean log eosinophils, N=451	-2.14 (1.04)	-13 (-56 to +30)
Age = 2 years		
Helicobacter stool +ve, N=589	29 (5)	-78 (-270 to +115)
Age = 3 years		
Mean log CRP, sd, N=614	-2.16 (2.71)	+2 (-9 to +12)
Log dengue IgG, N= 545	-0.43 (2.00)	+7 (-10 to +25)
Helicobacter stool +ve, N=775	51 (7)	-39 (-132 to +54)
Mean log eosinophils, N=652	-1.78 (1.26)	+22 (-5 to +49)
Toxoplasmosis serology +ve, N=606	362 (60)	+48 (-46 to +143)
Mean log IgE, N=614	+3.68 (1.57)	+8 (-21 to +37)
Medical diagnosis of dengue infection	78 (9)	-27 (-59 to +5)
**Current exposures**		
Male Sex	468 (52)	+36 (-23 to +96)
Mean age (months), (range)	74 (64 to 83)	+4 (-12 to +21)
Wheeze in past 12 months	353 (39)	-62 (-160 to +35)
**Mean weight, Kg, N=902 (range)**	**23.5 (11 to 51)**	**+11 (+3 to +18)**
**Current height, cm, N=903 (range)**	**119 (90 to 175)**	**+8 (+2 to +14)**
**Mean arm circumference, cm N=901**	**18.4 (2.4)**	**+12 (+1 to +24)**
Number of current smokers in house		
0	447 (49)	0
1	261 (29)	0 (-20 to +19)
≥ 2	195 (22)	-39 (-108 to +30)
Municipality of residence		
**Arroyo Naranjo**	**371 (41)**	**0**
**Cerro**	**119 (13)**	**+74 (+31 to 117)**
**Habana del Este**	**247 (27)**	**+48 (+39 to +57)**
**La Lisa**	**166 (18)**	**-95 (-126 to -64)**
		**p<0.001**
**Current infection and blood assays**		
Helicobacter stool antigen +ve, N=685	10 (1)	-10 (-228 to +207)
Toxoplasmosis +ve, N=688	437 (64)	+8 (-49 to +65)
Mean log dengue IgG serology, N=687	+1.63 (1.56)	-5 (-24 to +14)
Log CRP, N=686	-0.87 (1.38)	-9 (-37 to +20)
Mean log eosinophils, N=785	-1.61 (1.47)	-5 (-41 to +31)
Mean log IgE, N=688	+4.52 (0.97)	-12 (-87 to +63)
Toxocariasis +ve, N=667	57 (9)	-15 (-125 to +95)
Any atopy* (N=818)	52 (6)	-54 (-182 to +74)

adjusted for sex, age in months, current height and clustering by municipality
*defined as any allergen skin prick test > 3mm larger than saline control
FEV_1 = Forced Expiratory Volume in one second
CRP = C Reactive Protein, sd= standard deviation

For peer review only - http://bmjopen.bmj.com/site/about/guidelines.xhtml

For peer review only

Table 4. Association of exposures with bronchodilatation after inhaled salbutamol

Total number = 903	No (%, sd)	% change in FEV_1 (95% CI)
**Prior exposures**		
Any wheeze in 1st year of life	417 (46)	**+1.94 (+0.81 to +3.08)**
Family history of asthma	510 (56)	**+1.85 (+0.14 to +3.57)**
Attendance at nursery	138 (15)	-3.21 (-9.10 to +2.68)
Paracetamol in 1st year of life	213 (24)	-0.67 (-4.68 to +3.35)
Mean birth weight, Kg (sd) N=901	3.32 (0.53)	**-2.67 (-4.49 to -0.84)**
Mean birth height, cm	50 (2)	+0.06 (-0.56 to +0.69)
Breastfeeding ≥ 4months	503 (56)	+0.61 (-2.87 to +4.09)
Age = 1 year		
Mean log IgE, N=455	+3.42 (1.39)	**+1.68 (+0.54 to +2.82)**
Mean log eosinophils, N=449	-2.13 (1.04)	-0.16 (-2.54 to +2.21)
Age = 2 years		
Helicobacter stool +ve, N=586	28 (5)	-6.41 (-14.94 to +2.10)
Age = 3 years		
Mean log CRP, sd, N=615	-2.12 (2.71)	-0.28 (-0.88 to +0.32)
Log dengue IgG, N= 550	-0.43 (2.01)	-0.38 (-1.50 to +0.74)
Helicobacter stool +ve, N=776	51 (6)	-1.40 (-7.30 to +4.51)
Mean log eosinophils, N=653	-1.78 (1.26)	-0.39 (-4.45 to +3.68)
Toxoplasmosis serology +ve, N=607	362 (60)	-0.45 (-7.06 to +6.16)
Mean log IgE, N=615	+3.68 (1.57)	+0.23 (-1.81 to +2.27)
Medical diagnosis of dengue infection	78 (9)	-0.58 (-8.55 to +7.38)
**Current exposures**		
Male Sex	466 (52)	+2.22 (-0.28 to +4.71)
Age (months), (range)	74 (64 to 83)	-0.13 (-0.47 to +0.20)
Wheeze in past 12 months	353 (39)	+3.61 (-5.80 to +13.02)
Mean current weight, Kg, N=893 (range)	24 (11 to 51)	-0.23 (-0.54 to +0.08)
Current height, cm, N=893	119 (90 to 175)	-0.08 (-0.50 to +0.34)
Mean arm circumference, cm N=893	18.4 (2.4)	-0.14 (-0.81 to +0.54)
Number of current smokers in house		
0	446 (49)	0
1	263 (29)	-0.87 (-5.89 to +4.14)
≥ 2	194 (21)	+2.93 (-3.80 to +9.65)
Municipality of residence		
Arroyo Naranjo	368 (41)	0
Cerro	119 (13)	**-1.34 (-2.56 to -0.11)**
Habana del Este	249 (28)	**-0.07 (-0.46 to +0.31)**
La Lisa	167 (18)	**+6.24 (+5.56 to +6.91)**
		p=0.002
**Current infection and blood assays**		
Helicobacter stool antigen +ve, N=684	10 (1)	+8.89 (-9.17 to +26.96)
Toxoplasmosis +ve, N=687	438 (64)	-0.47 (-3.34 to +2.39)
Mean log dengue IgG serology, N=686	+1.64 (1.56)	+0.30 (-1.44 to +2.03)
Log CRP, N=685	-0.87 (1.40)	+0.37 (-1.64 to +2.38)
Mean log eosinophils, N=782	-161 1.47)	+0.99 (-0.48 to+2.46)
Mean log IgE, N=686	+4.52 (0.98)	+0.64 (-0.18 to +1.45)
Toxocariasis +ve, N=666	58 (9)	-1.03 (-5.29 to +3.23)
Any atopy* (N=816)	52 (6)	-2.75 (-10.15 to +4.60)

adjusted for sex, age in months and clustering by municipality
*defined as any allergen skin prick test > 3mm larger than saline control
FEV_1 = Forced Expiratory Volume in one second, CRP = C Reactive Protein
sd= standard deviation

For peer review only - http://bmjopen.bmj.com/site/about/guidelines.xhtml

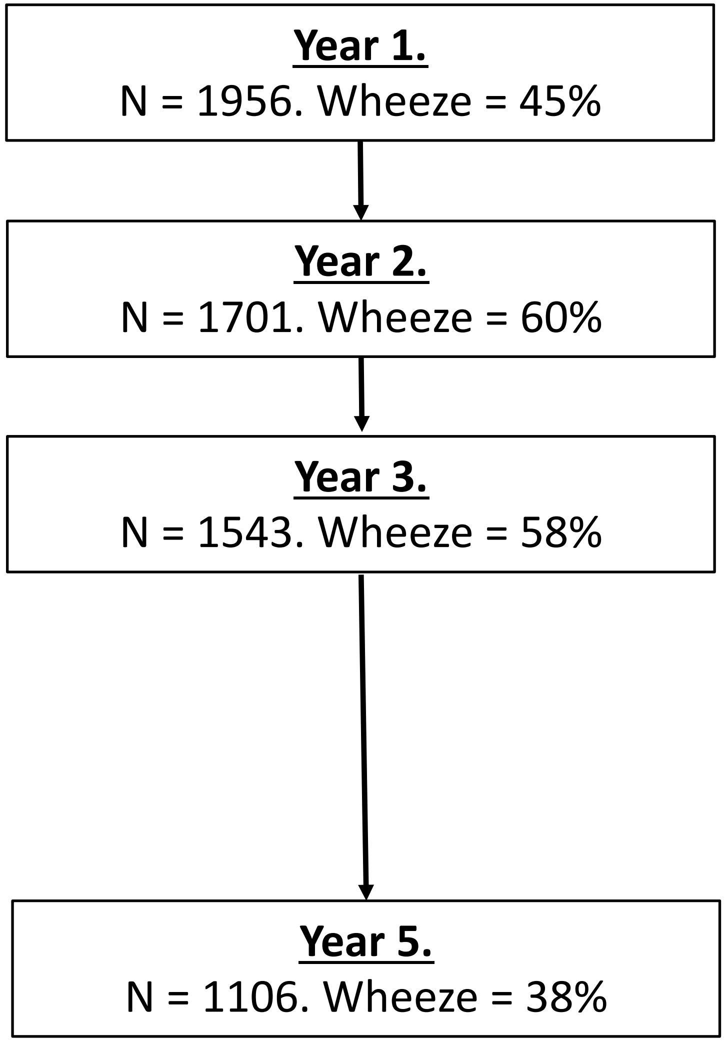
Year 1.
N = 1956. Wheeze = 45%
Year 2.
N = 1701. Wheeze = 60%
Year 3.
N = 1543. Wheeze = 58%
Year 5.
N = 1106. Wheeze = 38%

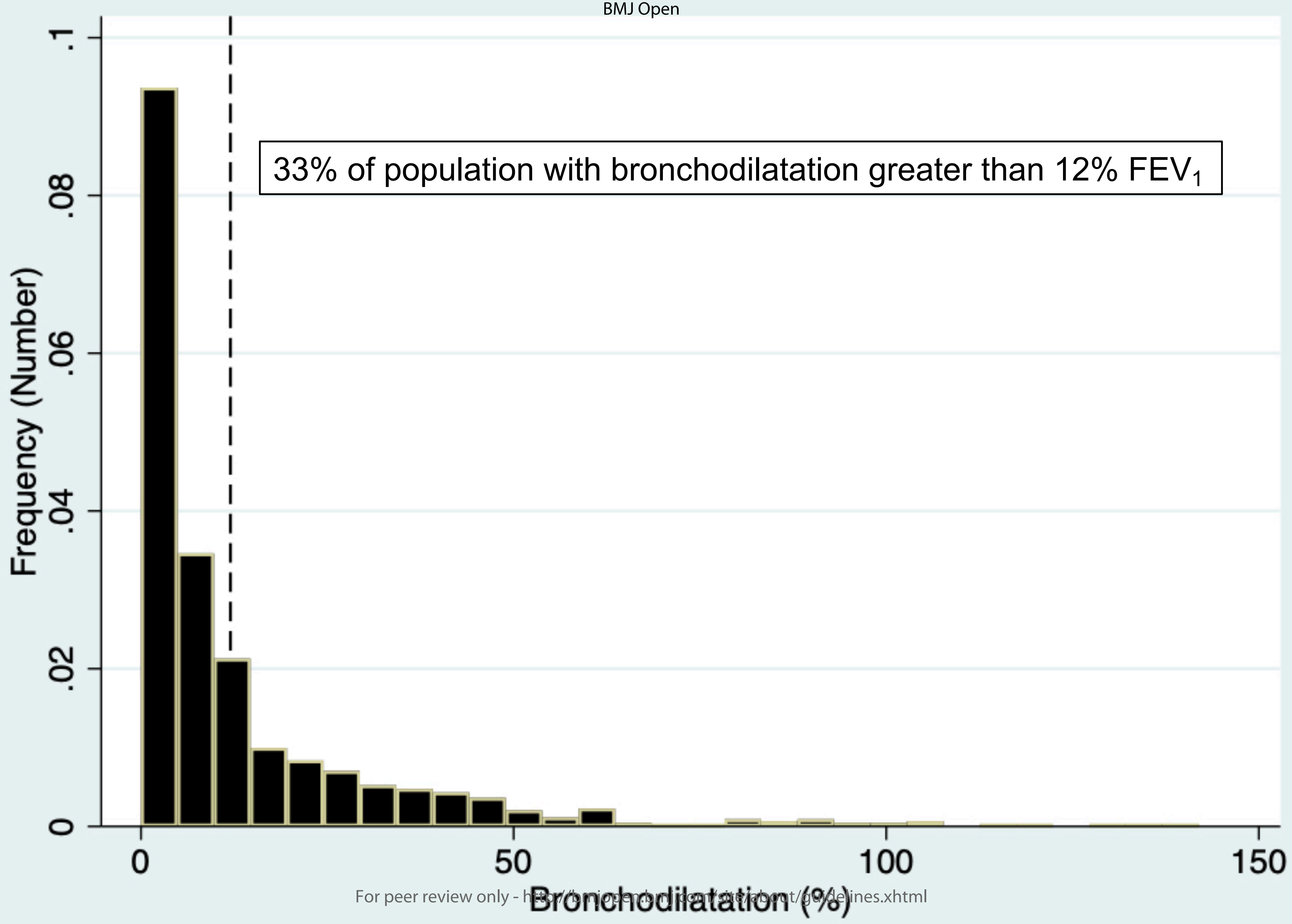
33% of population with bronchodilatation greater than 12% FEV1
Frequency (Number)
0
.02
.04
.06
.08
.1
0
50
100
150
Bronchodilatation (%)

STROBE Statement

	Item No	Recommendation
**P1/2 Title and abstract**	1	(*a*) Indicate the study's design with a commonly used term in the title or the abstract
		(*b*) Provide in the abstract an informative and balanced summary of what was done and what was found
**Introduction**		
P4 Background/rationale	2	Explain the scientific background and rationale for the investigation being reported
P4 Objectives	3	State specific objectives, including any prespecified hypotheses
**Methods**		
P4 Study design	4	Present key elements of study design early in the paper
P4 Setting	5	Describe the setting, locations, and relevant dates, including periods of recruitment, exposure, follow-up, and data collection
P4 Participants	6	(*a*) Give the eligibility criteria, and the sources and methods of selection of participants
P5 Variables	7	Clearly define all outcomes, exposures, predictors, potential confounders, and effect modifiers. Give diagnostic criteria, if applicable
P5 Data sources/ measurement	8*	For each variable of interest, give sources of data and details of methods of assessment (measurement). Describe comparability of assessment methods if there is more than one group
P6 Bias	9	Describe any efforts to address potential sources of bias
P4 Study size	10	Explain how the study size was arrived at
P5 Quantitative variables	11	Explain how quantitative variables were handled in the analyses. If applicable, describe which groupings were chosen and why
P5 Statistical methods	12	(*a*) Describe all statistical methods, including those used to control for confounding
		(*b*) Describe any methods used to examine subgroups and interactions
		(*c*) Explain how missing data were addressed
		(*d*) If applicable, describe analytical methods taking account of sampling strategy
		(*e*) Describe any sensitivity analyses
**Results**		
P16 Participants	13*	(a) Report numbers of individuals at each stage of study—eg numbers potentially eligible, examined for eligibility, confirmed eligible, included in the study, completing follow-up, and analysed
		(b) Give reasons for non-participation at each stage
		(c) Consider use of a flow diagram
P18 Descriptive data	14*	(a) Give characteristics of study participants (eg demographic, clinical, social) and information on exposures and potential confounders
		(b) Indicate number of participants with missing data for each variable of interest
P18-20 Outcome data	15*	Report numbers of outcome events or summary measures
P18-20 Main results	16	(*a*) Give unadjusted estimates and, if applicable, confounder-adjusted estimates and their precision (eg, 95% confidence interval). Make clear which confounders were adjusted for and why they were included
		(*b*) Report category boundaries when continuous variables were categorized
		(*c*) If relevant, consider translating estimates of relative risk into absolute risk for a meaningful time period

For peer review only - http://bmjopen.bmj.com/site/about/guidelines.xhtml

P18-20 Other analyses	17	Report other analyses done—eg analyses of subgroups and interactions, and sensitivity analyses
**Discussion**		
P8 Key results	18	Summarise key results with reference to study objectives
P8 Limitations	19	Discuss limitations of the study, taking into account sources of potential bias or imprecision. Discuss both direction and magnitude of any potential bias
P9 Interpretation	20	Give a cautious overall interpretation of results considering objectives, limitations, multiplicity of analyses, results from similar studies, and other relevant evidence
P10 Generalisability	21	Discuss the generalisability (external validity) of the study results
**Other information**		
P12 Funding	22	Give the source of funding and the role of the funders for the present study and, if applicable, for the original study on which the present article is based

*Give information separately for exposed and unexposed groups.

Note: An Explanation and Elaboration article discusses each checklist item and gives methodological background and published examples of transparent reporting. The STROBE checklist is best used in conjunction with this article (freely available on the Web sites of PLoS Medicine at http://www.plosmedicine.org/, Annals of Internal Medicine at http://www.annals.org/, and Epidemiology at http://www.epidem.com/). Information on the STROBE Initiative is available at www.strobe-statement.org.

For peer review only
